# Alpha radioimmunotherapy using ^225^Ac-proteus-DOTA for solid tumors - safety at curative doses

**DOI:** 10.7150/thno.48810

**Published:** 2020-09-14

**Authors:** Sarah M. Cheal, Michael R. McDevitt, Brian H. Santich, Mitesh Patel, Guangbin Yang, Edward K. Fung, Darren R. Veach, Meghan Bell, Afruja Ahad, Daniela Burnes Vargas, Blesida Punzalan, Naga Vara Kishore Pillarsetty, Hong Xu, Hong-fen Guo, Sébastien Monette, Adam O. Michel, Alessandra Piersigilli, David A. Scheinberg, Ouathek Ouerfelli, Nai-Kong V. Cheung, Steven M. Larson

**Affiliations:** 1 Molecular Pharmacology Program, Memorial Sloan Kettering Cancer Center, New York, NY.; 2 Department of Radiology, Memorial Sloan Kettering Cancer Center, New York, NY.; 3 Department of Pediatrics, Memorial Sloan Kettering Cancer Center, New York, NY.; 4 Louis V. Gerstner Jr. Graduate School of Biomedical Sciences, Memorial Sloan Kettering Cancer Center, New York, NY.; 5 Organic Synthesis Core Facility, Memorial Sloan Kettering Cancer Center, New York, NY.; 6 Department of Medical Physics, Memorial Sloan Kettering Cancer Center, New York, NY.; 7 Laboratory of Comparative Pathology, Memorial Sloan Kettering Cancer Center, Weill Cornell Medicine, and The Rockefeller University, New York, NY.; 8 Immunology Program, Weill Cornell Medicine, New York, NY.; 9 Department of Radiology, Weill Cornell Medicine, New York, NY

**Keywords:** Ac-225, pretargeted radioimmunotherapy, pretargeting, radioimmunotherapy, TAT

## Abstract

This is the initial report of an α-based pre-targeted radioimmunotherapy (PRIT) using ^225^Ac and its theranostic pair, ^111^In. We call our novel tumor-targeting DOTA-hapten PRIT system “proteus-DOTA” or “Pr.” Herein we report the first results of radiochemistry development, radiopharmacology, and stoichiometry of tumor antigen binding, including the role of specific activity, anti-tumor efficacy, and normal tissue toxicity with the Pr-PRIT approach (as α-DOTA-PRIT). A series of α-DOTA-PRIT therapy studies were performed in three solid human cancer xenograft models of colorectal cancer (GPA33), breast cancer (HER2), and neuroblastoma (GD2), including evaluation of chronic toxicity at ~20 weeks of select survivors.

**Methods:** Preliminary biodistribution experiments in SW1222 tumor-bearing mice revealed that ^225^Ac could not be efficiently pretargeted with current DOTA-Bn hapten utilized for ^177^Lu or ^90^Y, leading to poor tumor uptake *in vivo*. Therefore, we synthesized Pr consisting of an empty DOTA-chelate for ^225^Ac, tethered via a short polyethylene glycol linker to a lutetium-complexed DOTA for picomolar anti-DOTA chelate single-chain variable fragment (scFv) binding. Pr was radiolabeled with ^225^Ac and its imaging surrogate, ^111^In. *In vitro* studies verified anti-DOTA scFv recognition of [^225^Ac]Pr, and *in vivo* biodistribution and clearance studies were performed to evaluate hapten suitability and *in vivo* targeting efficiency.

**Results:** Intravenously (i.v.) administered ^225^Ac- or ^111^In-radiolabeled Pr in mice showed rapid renal clearance and minimal normal tissue retention. *In vivo* pretargeting studies show high tumor accumulation of Pr (16.71 ± 5.11 %IA/g or 13.19 ± 3.88 %IA/g at 24 h p.i. for [^225^Ac]Pr and [^111^In]Pr, respectively) and relatively low uptake in normal tissues (all average ≤ 1.4 %IA/g at 24 h p.i.). Maximum tolerated dose (MTD) was not reached for either [^225^Ac]Pr alone or pretargeted [^225^Ac]Pr at administered activities up to 296 kBq/mouse. Single-cycle treatment consisting of α-DOTA-PRIT with either huA33-C825 bispecific anti-tumor/anti-DOTA-hapten antibody (BsAb), anti-HER2-C825 BsAb, or hu3F8-C825 BsAb for targeting GPA33, HER2, or GD2, respectively, was highly effective. In the GPA33 model, no complete responses (CRs) were observed but prolonged overall survival of treated animals was 42 d for α-DOTA-PRIT vs. 25 d for [^225^Ac]Pr only (*P* < 0.0001); for GD2, CRs (7/7, 100%) and histologic cures (4/7, 57%); and for HER2, CRs (7/19, 37%) and histologic cures (10/19, 56%) with no acute or chronic toxicity.

**Conclusions:** [^225^Ac]Pr and its imaging biomarker [^111^In]Pr demonstrate optimal radiopharmacologic behavior for theranostic applications of α-DOTA-PRIT. For this initial evaluation of efficacy and toxicity, single-cycle treatment regimens were performed in all three systems. Histologic toxicity was not observed, so MTD was not observed. Prolonged overall survival, CRs, and histologic cures were observed in treated animals. In comparison to RIT with anti-tumor IgG antibodies, [^225^Ac]Pr has a much improved safety profile. Ultimately, these data will be used to guide clinical development of toxicity and efficacy studies of [^225^Ac]Pr, with the goal of delivering massive lethal doses of radiation to achieve a high probability of cure without toxicity.

## Introduction

Pretargeted radioimmunotherapy (PRIT) combines the desirable tumor-targeting features of antibodies 1 with the ideal phamacokinetics of small-molecule radiohaptens 2 for high-therapeutic index (TI) radioimmunotherapy (RIT) 3. PRIT has been investigated for decades as an alternative to conventional RIT with radiolabeled-IgG; popular approaches include the strept(avidin)-biotin system, bispecific anti-tumor/anti-hapten antibodies (BsAb) and radiohaptens, antibody-oligonucleotide conjugates and radiolabeled complementary oligonucleotides, and, more recently, bioorthogonal approaches (e.g., inverse-electron-demand Diels-Alder reaction and adamantane/cucurbituril system) 4-11.

Because curative therapy is a major need in solid human tumors of adults and children, we have focused our efforts on PRIT for common solid tumors, using clinically applicable antibody-based radiotargeting systems 3. We call this development “DOTA-PRIT.” DOTA-PRIT utilizes anti-tumor antigen/anti-1,4,7,10-tetraazacyclododecane-N, N', N'', N'''-tetraacetic acid (DOTA) hapten BsAbs (IgG-single-chain variable fragment (scFv) format 12) based on the pioneering PRIT “2D12.5” anti-metal chelate antibody for nanomolar binding to DOTA chelates of all lanthanides 13, 14. We utilize an affinity maturated anti-DOTA antibody scFv “C825” for picomolar binding to DOTA-radiohaptens for improved *in vivo* radioactive bound-lifetimes of tumor-targeted radioisotopes during PRIT 15. In this technique, the non-radioactive BsAb formulation is infused and, owing to long circulation times, it takes a couple of days to reach high a tumor-to-background ratio 12. Once the clearing agent (CA) 16 has reduced unbound circulating anti-tumor antibody, the radiohapten in the form of radiometals carried by a DOTA-hapten 2 (molecular weight (MW) ~1 kDa) is infused and rapidly taken up by the pretargeted scFv on the BsAb. Any non-tumor-bound radiometal-DOTA complex is efficiently and rapidly cleared through the kidney, resulting in exceptionally high TI.

We have successfully applied DOTA-PRIT to a variety of solid tumor-associated antigens for high-TI targeting of β-emitting isotopes, including GD2 17, GPA33 18, 19, and HER2 20 (β-DOTA-PRIT). In our hands, β-DOTA-PRIT now reliably leads to cures in animal tumors with minimal radiotoxicity. Also, for hematological cancers, β-DOTA-PRIT with ^90^Y has also been shown to be curative and safe in preclinical models 21, 22.

One important limitation of DOTA-PRIT is that the methodology is confined to the β-emitting radiolanthanides, ^177^Lu, and ^90^Y, due to the hapten-binding scFv C825 antibody specificity 15. In the present manuscript, we report a novel set of radiohaptens that permits the use of other forms of therapeutic radionuclides - in particular, α-emitters for histologic cure.

^225^Ac is a long-lived (*t*_1/2_ = 9.92 d) α-particle emitter that has been extensively investigated as an ^225^Ac-radiolabelled anti-tumor antigen IgG (^225^Ac-IgG) drug platform for α-radioimmunotherapy (α-RIT; 3, 23-27). However, for solid tumors, with the long serum half-life of IgG in blood and tissues, α-RIT with ^225^Ac-IgG has a limited TI, a common characteristic of large carriers with long biologic half-lives, such as IgG. In fact, ^225^Ac-IgG has been shown to be quite radiotoxic to kidneys of mice 28. To resolve this problem, we reasoned that a radiohapten—with its short serum half-life and complete renal excretion, without retention—would reduce *in vivo* radiation exposure and be the ideal carrier for the ^225^Ac as part of DOTA-PRIT (α-DOTA-PRIT) 2.

We evaluated the hypothesis that a novel radiohapten precursor that we call “proteus-DOTA” (Pr) is a suitable chelator for the α-emitter ^225^Ac, with pharmacodynamics that mimic our “gold standard” β-radiohapten [^177^Lu]LuDOTA-Bn. We also determined if Pr was suitable with ^111^In, a radioisotope that has been used as an imaging biomarker for ^225^Ac-IgG 29. We tested both the activity and toxicity of α-DOTA-PRIT in mouse xenografts of human colorectal cancer (GPA33-expressing SW1222), breast cancer (HER2-expressing BT-474), and neuroblastoma (GD2-expressing IMR-32) as models for α-therapy of human solid tumors.

## Methods

Please see Supplemental Information (SI) for additional details regarding: animal models, Pr synthesis, cell culture, *in vitro* binding assays with [^225^Ac]Pr and BsAb, *in vivo* studies [^225^Ac]Pr-BsAb complex, single-photon emission computed tomography/computed tomography (SPECT/CT) image analysis, macroscopic post-mortem examination and tissue sample collection, and histopathology (microscopic evaluation).

### Animal care and models

Female athymic nude mice (strain: Hsd:Athymic Nude-Foxn^1nu^, Envigo, aged 6-8 weeks, average weight for six-week-old and eight-week-old animals: 18.6 and 21.0 g, respectively) were used for all experiments. The subcutaneous BT-474 tumor model 20, SW1222 tumor model 18, 19, and IMR32 or luciferase gene reporter transfected IMR32 (IMR-32/luc) tumor model 17 (~50-900 mm^3^ by caliper measurement, assuming ellipsoid geometry for calculation of tumor volume) was used for targeting of HER2, GPA33, or GD2 antigens, respectively, with minor modifications (described in SI). All animal experiments were done in accordance with protocols approved by the Institutional Animal Care and Use Committee of Memorial Sloan Kettering Cancer Center following National Institutes of Health guidelines for animal welfare.

### DOTA-PRIT reagents and protocols

For all DOTA-PRIT studies, mice were given three separate intravenous (i.v.) injections: (1) 250 µg (1.19 nmol) of anti-tumor/C825 BsAb (anti-GPA33: huA33-C825 18, 19; anti-HER2: anti-HER2-C825 20; anti-GD2: hu3F8-C825 17) 28 h prior to radiohapten administration; and (2) CA (dextran-CA: 62.5 µg, 0.125 nmol dextran, 7.625 nmol (Y)DOTA (unless otherwise noted) *or,* alternatively, dendrimer-CA: 25 µg, 2.76 nmol (Y)DOTA 30) 4 h prior to administration of (3) radiohapten (as described). Note: the dendrimer-CA was recently developed by our group as an alternative to the dextran-CA in anticipation of clinical translation, and was found to have a similar impact on *in vivo* pretargeting of [^177^Lu]LuDOTA-Bn 30. All reagents were formulated for injection in 200-250 µL normal sterile isotonic saline solution (NSS). For all injections, mice were gently warmed with a heat lamp and placed on a restrainer. Their tails were sterilized with alcohol pads, and single-bolus injections were placed into the lateral tail vein.

### Biodistribution experiments

For biodistribution assay following radiotracer injection, mice were euthanized by CO_2_ (g) asphyxiation and tumor and selected organs were harvested, rinsed with water and allowed to air dry, weighed, and radioassayed by gamma scintillation counting (Perkin Elmer Wallac Wizard 3”). Count rates were background- and decay-corrected, converted to activities using a system calibration factor specific for the isotope, normalized to the administered activity, and expressed as percent injected activity per gram of tissue (%IA/g). For ^225^Ac, each sample was counted for up to 10 min (24 h after collection when secular equilibrium was reached) using a 150 to 600 keV energy window.

### Preliminary testing of DOTA-PRIT with [^225^Ac]DOTA-Bn

For initial pretargeting of [^225^Ac]DOTA-Bn, the GD2 system was used as a model. The GD2 model was used for preliminary testing of DOTA-PRIT with [^225^Ac]DOTA-Bn since we determined for [^177^Lu]LuDOTA-Bn, the TI for tumor-to-blood to be highest in that model (TI = 142) 17 in comparison to HER2 (TI = 28) 20 and GPA33 (TI = 73) 19. Ten IMR-32 tumor-bearing mice were injected with BsAb, dextran-CA (100 µg, 0.2 nmol dextran, 29 nmol of (Y)DOTA), and then an equimolar amount of either [^225^Ac]DOTA-Bn 31 or, for comparison, [^177^Lu]LuDOTA-Bn 17 (1.85 MBq and 3.7 MBq of ^177^Lu and ^225^Ac, respectively, 8-10 pmol; *n* = 5/radiohapten). Mice were sacrificed at 24 h post-injection (p.i.) of radiotracer for biodistribution assay. *Note:* While these different administered radioactivity doses potentially result in different anti-tumor efficacies, within 24 h post-injection we did not anticipate any effects on tumor uptake and retention of the respective radiohaptens at equimolar doses in the established tumors.

### Pr synthesis

Pr consists of a three-arm DOTA radiometal-chelating agent (DO3A) separated by a tetraethylene glycol (PEG_4_) linker to a ^175^Lu (natural) lutetium complex of 2-benzyl-DOTA. Pr was synthesized by linking two bifunctional chelators: commercial 2,2',2''-(10-(17-amino-2-oxo-6,9,12,15-tetraoxa-3-azaheptadecyl)-1,4,7,10-tetraazacyclododecane-1,4,7-triyl) triacetic acid (NH_2_-PEG_4_-DO3A) and non-radioactive lutetium 2-(4-isothiocyanatobenzyl)-1,4,7,10-tetraazacyclododecane-tetraacetic acid (*p*-SCN-Bn-DOTA·Lu^3+^ complex) prepared from commercial *p*-SCN-Bn-DOTA and ^175^LuCl_3_·6 H_2_O. The final product was purified using semi-preparative reverse-phase (RP) C-18 high-pressure liquid chromatography (HPLC) and characterized by liquid-chromatography/mass spectrometry (LC/MS) and nuclear magnetic resonance (NMR).

### Pr radiochemistry and assay of C825 binding of [^225^Ac]Pr

The ^225^Ac used in this research was supplied by the United States Department of Energy Office of Science by the Isotope Program in the Office of Nuclear Physics. Carrier-free ^225^Ac (2.146 E6 GBq/g [5.80 E4 Ci/g]) was obtained from Oak Ridge National Laboratory as a dried nitrate residue. The [^225^Ac]AcNO_3_ was dissolved in 0.2 M Optima grade hydrochloric acid for subsequent radiochemistry. ^225^Ac-activity measurements were made at secular equilibrium using a CRC-15R radioisotope calibrator (Capintec, Inc.) set at 775 and multiplied the displayed activity value by 5; samples were positioned at the bottom and center of the well for measurement. No-carrier-added [^111^In]InCl_3_ sterile solution was obtained from Nuclear Diagnostic Products, Inc.^ 111^In-radioactivity measurements were made using a CRC-15R radioisotope calibrator (Capintec, Inc.) with the manufacturer's recommended settings for the isotope. Water and buffers were rendered metal-free and sterile by passing them through a column of Chelex-100 resin, followed by filtration through a sterile-filter device (0.22- or 0.45-µM). Initially, Pr was suspended in chelexed water at 10 mg/mL and immediately transferred to a 1.8-mL Nunc vial, and any unused stock was promptly stored at -20°C.

A typical synthesis of [^225^Ac]Pr is described, with a summary of [^225^Ac]Pr preparations provided in Table S1. To prepare [^225^Ac]Pr, 20 µL of [^225^Ac]AcNO_3_ (2.442 MBq) in 0.2M HCl was mixed with 100 µL of 10 mg/mL Pr (1 mg; 0.74 µmoles) in a 1.8-mL Nunc vial. Next, 15 µL of L-ascorbic acid solution (150 g/L) and 100 µL of 3M NH_4_OAc solution was added. The pH of the solution was verified to be ~5.5 by spotting 1 µL of the reaction mixture onto pHydrion pH paper (range, 5.0-9.0). The reaction was incubated at 60°C for 30 min, and then purified using a Sephadex C-25 column pre-equilibrated with 6 mL of NSS. The reaction mixture was added to the column and eluted with 4 mL of NSS to recover all removable activity ([^225^Ac]Pr; note: the % activity that washed off the resin was the % ^225^Ac that was complexed by Pr). The activity of [^225^Ac]Pr was determined to be 2.294 MBq, giving a radiochemical yield (RCY) of 94%. The final molar activity was 3108 GBq/mol. In summary, for [^225^Ac]Pr, the RCY ranged from 91-100% and was prepared to a final molar activity of 3.11E3-1.69E5 GBq/mol.

Using similar radiochemical methods, [^111^In]Pr was prepared from [^111^In]InCl_3_ (153-249 MBq) and 1-150 µL of 10 mg/mL Pr (10 µg-1.5 mg; 7.42 nmol-1.11 µmol). Instead of Sephadex C-25 chromatography, the crude reaction was purified by Strata-X 33 µm polymeric RP solid phase extraction column (Phenomenex). The RCY was calculated based on the eluted product activity, and ranged from 44->98%. The final molar activity of [^111^In]Pr ranged from 2.28E5-9.67E6 GBq/mol.

Radiochemical purity (RCP) of [^225^Ac]Pr and [^111^In]Pr was evaluated by radio-HPLC using a Shimadzu Prominence HPLC system controlled by LC Solution v1.25 software and comprising an LC-20AB dual-pump module, DGU-20A3R degasser, SIL-20ACHT autosampler, SPD-20A UV-Vis detector, and Bioscan Flow-Count B-FC-1000 with PMT/NaI radioactivity detector in-line. Separations were run on an analytical 4.6 × 250 mm Gemini-NX C18 or Fusion RP C18 HPLC column (Phenomenex, Inc. Torrance, CA). HPLC conditions were as follows: solvent A - 10 mM pH 5 NH_4_OAc, B - CH_3_CN, 1.0 mL/min flow rate, λ = 220 nm, injection volume 5-20 µL, and gradient: 0% B to 40% B over 10 min. Samples were diluted 1:5 in 1 mM ethylenediaminetetraacetic acid (EDTA) prior to analysis. The radiochemical purity of [^225^Ac]Pr and [^111^In]Pr was ≥91% by radio-HPLC (Figure S1).

*In vitro* binding assays of [^225^Ac]Pr were performed with anti-HER2 BsAb to evaluate C825 binding of [^225^Ac]Pr, followed by *in vivo* evaluation in BT-474 tumor-bearing mice to assay HER2 binding of the [^225^Ac]Pr-BsAb complex. We used the HER2 model because of the three antibody-antigen systems currently available to us for DOTA-PRIT, radiolabeled forms of trastuzumab have been studied most extensively in preclinical and clinical studies 3, 24, 32, 33.

### Pr blood half-life and whole-body clearance

Five tumor-free mice were administered [^111^In]Pr (740 kBq/3.38 nmol) and blood was collected via the tail vein at *t* = 5, 15, 30, 60, 120, and 240 min p.i. of tracer, followed by a biodistribution study at the final time point (240 min or 4 h). For comparison of the *in vivo* behavior of [^225^Ac]Pr and [^111^In]Pr, groups of tumor-free mice (*n* = 3/tracer) were given [^225^Ac]Pr (1.85 kBq, 198 pmol) or [^111^In]Pr (740 kBq, 200 pmol) and sacrificed 60 min p.i. for the biodistribution assay. To evaluate non-specific tumor uptake of Pr, two BT-474 tumor-bearing mice were administered [^111^In]Pr (10.2 MBq and 9.50 MBq; ~300 pmol) and imaged with a dedicated small-animal SPECT/CT (NanoSPECT/CT, Bioscan, Washington, DC) at 60 min and 6 h p.i. of tracer according to previous methods 18. In addition, serial whole-body activity measurements were collected immediately following imaging and a biodistribution assay was performed at 24 h p.i. Whole-body activity and blood activity clearance parameters were determined by fitting the data to exponential model curves using MATLAB (Mathworks, Inc.).

### DOTA-PRIT with [^225^Ac]Pr or [^111^In]Pr

For initial pretargeting of [^225^Ac]Pr or [^111^In]Pr, the GPA33 system was used as a model. We used the GPA33 model for preliminary testing of DOTA-PRIT with radiolabeled forms of Pr since we have conducted the most *in vivo* experiments with this antibody-antigen system and tumor model in comparison with GD2/IMR32 and HER2/BT-474, and thus consider it to be a reliable test system for the development of novel radiohaptens. Three SW1222 tumor-bearing mice were given BsAb, dextran-CA, and [^225^Ac]Pr (1.85 kBq, 182 pmol) and sacrificed at 24 h p.i. for the biodistribution assay. For comparison, pretargeting of [^111^In]Pr was also studied. Five SW1222 tumor-bearing mice were given BsAb, dendrimer-CA, and [^111^In]Pr (*n* = 4, 1.67 MBq, 172 pmol *or n* = 1, 7.66 MBq, 790 pmol) for the biodistribution studies (all at 24 h p.i.), and SPECT/CT imaging (at 20 h p.i.), respectively.

Furthermore, biodistribution experiments were conducted at 24 h p.i. as described above with BsAb, CA, and a radiohapten ([^225^Ac]Pr, [^111^In]Pr, and [^177^Lu]LuDOTA-Bn) dose spanning two orders of magnitude (~170-26900 pmol) in order to determine the upper limit of tumor uptake. From the biodistribution data, a plot of tumor uptake at 24 h p.i. *versus* administered pmol of hapten was prepared, and nonlinear regression analysis (one site, specific binding) was performed using Prism 7 (GraphPad, Inc.) to estimate the Bmax.

### Toxicity and therapy studies

An initial dose escalation toxicity study was performed with varying dose levels of [^225^Ac]Pr (0, 9.25, 18.5, 37, 74, 148, or 296 kBq/mouse; 910 pmol-29.1 nmol) in groups (*n* = 5) of tumor-free mice to determine maximum tolerated dose (MTD). This dose range was based on a previous MTD study in BALB/c mice with the PRIT reagent [^225^Ac]DOTA-Biotin, where lethal toxicity was observed at 110 d after treatment with 740 kBq/mouse 34. Treated mice were monitored daily and weighed up to twice weekly for evidence of treatment-induced toxicity for 145 d (~20 w) p.i. All survivors were evaluated for radiation-induced histologic organ damage by board-certified veterinary pathologists.

All α-DOTA-PRIT studies were performed with single-cycle treatment regimens. Immediately prior to the start of treatment, tumor-bearing mice were randomized into treatment groups based on tumor volume in order to create groups with approximately equal average tumor volumes. Weight loss of 20% or a tumor diameter exceeding 10 mm (BT-474 or IMR-32/luc) or 15 mm (SW1222) was set as an endpoint. For BT-474 and IMR-32/luc, histological analysis of the site of tumor inoculation was carried out in survivors at study endpoints (for BT-474: ~130 d post-treatment; for IMR-32/luc: ~125 d post-treatment); for SW1222, no survivors were analyzed at study endpoint (~60 d post-inoculation/47 d post-treatment), as all (*n* = 6) had significant tumor that was apparently progressing (tumor volumes: 434 mm^3^, 683 mm^3^, 1023 mm^3^, 1122 mm^3^, 1172 mm^3^, and 1227 mm^3^). In order to establish benchmarks for toxicity and efficacy, 296 kBq (26.9 nmol) activity of pretargeted [^225^Ac]Pr or [^225^Ac]Pr alone (i.e., without BsAb and CA injections) was injected in groups of SW1222 tumor-bearing mice (*n* = 5-13) or groups of BT-474 tumor-bearing mice (*n* = 10-19). In addition, BT-474 tumor-bearing mice (*n* = 5) were treated with BsAb only to control for anti-tumor effects of the anti-HER2 portion of the BsAb 20. Note: we previously demonstrated that the anti-GPA33 BsAb 19 and anti-GD2 BsAb 17 are ineffective to control tumor growth in those respective models; therefore, those treatment controls were omitted during this study. For treatment of IMR-32, 37 kBq (2.2 nmol or 2.4 nmol) activity pretargeted [^225^Ac]Pr or [^225^Ac]Pr alone injected in groups (*n* = 6-7) of IMR-32/luc tumor-bearing mice. A lower administered activity of [^225^Ac]Pr was used during this treatment study since these xenografted mouse tumors were shown to be most radiosensitive during GD2 β-DOTA-PRIT compared to the SW1222- and BT-474-tumor models 17, although we note that the relative biologic effectiveness (RBE) of radiation of different linear energy transfers (LETs) (i.e., treatment with ^225^Ac versus ^177^Lu) likely varies among the different tumor models*.* At 110-130 d post-tumor inoculation/68-88 d post-treatment, the presence of viable IMR-32/luc cells at the site of xenograft inoculation was recorded by measuring luciferase activity using bioluminescence imaging (BLI) as described previously 17.

### Hematologic toxicity and chronic tissue histopathology

For hematology of select therapy studies, blood was collected via retroorbital blood draws in tubes containing EDTA just prior to treatment start, as well as at weekly intervals up to 5 w (*n* = 4-5 mice/point). Automated analysis was performed on a Drew Scientific HemaVet 950FS (blood parameters analyzed: white blood cell (WBC), neutrophils (NE)#, NE%, lymphocytes (LY)#, LY%, monocytes (MO)#, MO%, eosinophils (EO)#, EO%, basophils (BA)#, BA%, red blood cell (RBC), hemoglobin (HGB), hematocrit (HCT), mean corpuscular volume (MCV), mean corpuscular hemoglobin (MCH), mean corpuscular hemoglobin concentration (MCHC), red cell distribution width (RDW), platelets (PLT), and mean platelet volume (MPV)). To examine chronic toxicity, a total of 19 mice were examined from the HER2 α-DOTA-PRIT and GD2 α-DOTA-PRIT therapy studies. From the HER2 α-DOTA-PRIT study, 10 mice were evaluated at 150 d p.i. of either: anti-HER2 α-DOTA-PRIT (296 kBq) (*n* = 6) or control treatments (*n* = 4; consisting of: no treatment, injection of BsAb only, or injection of [^225^Ac]Pr (296 kBq) only). From the GD2 α-DOTA-PRIT study, a total of 9 mice were evaluated at 141-241 d p.i. of either: anti-GD2 α-DOTA-PRIT (37 kBq) (*n* = 4) or tumor-free age-matched littermate controls (*n* = 5).

### Statistical analysis

Differences in tissue uptake between cohorts were statistically analyzed with the student t test for unpaired data using Excel. Two-sided significance levels were calculated and a P value of <0.05 was considered statistically significant. Survival analysis was performed using Prism 7 (GraphPad, Inc.). Kaplan-Meier survival curves were analyzed with Mantel-Cox test. Data is presented as mean and standard deviation (SD).

## Results

### Preliminary DOTA-PRIT with [^225^Ac]DOTA-Bn, Pr synthesis and radiochemistry, and verification of C825 recognition of Pr

**Figure 1:**
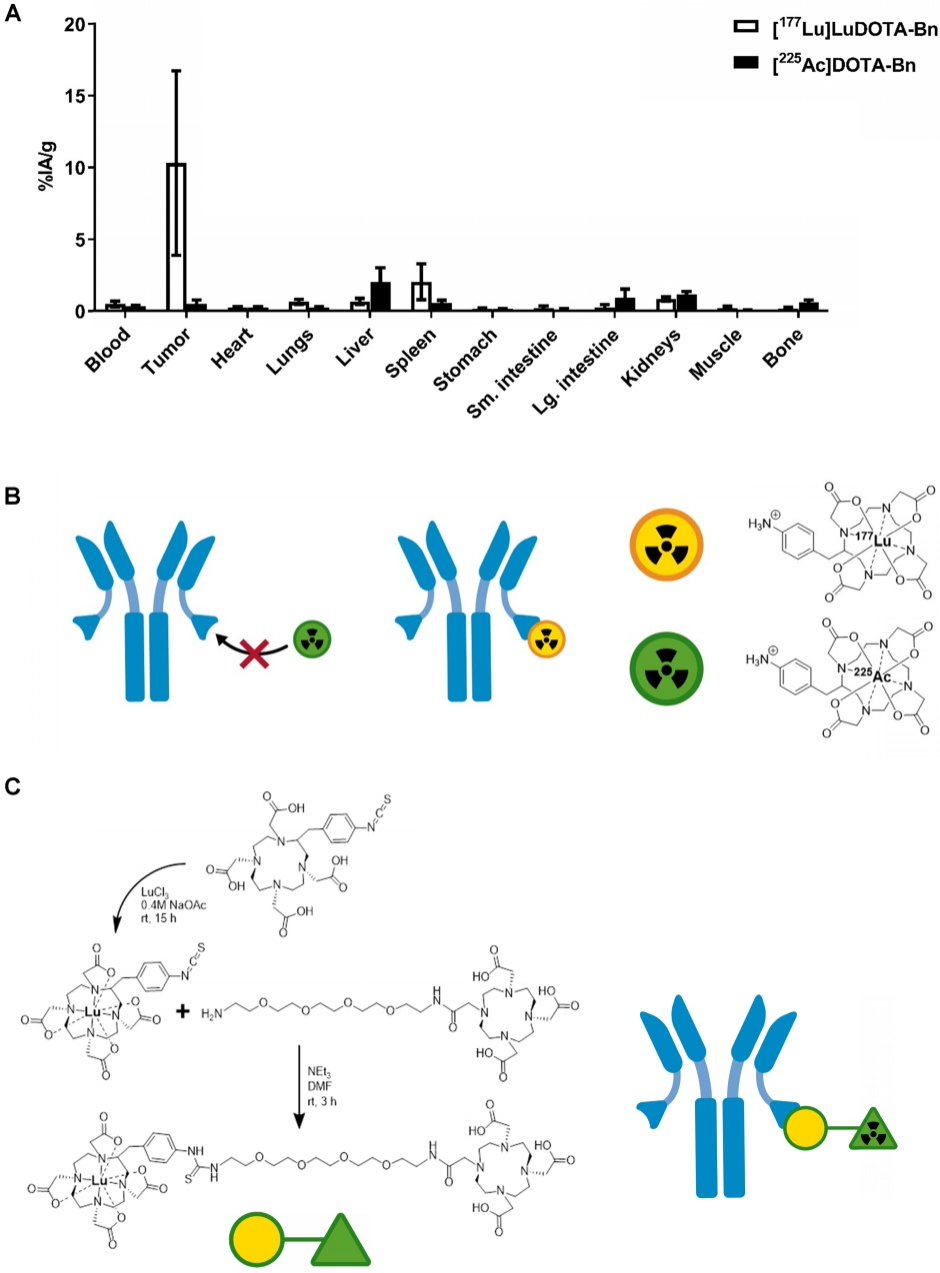
** Preliminary *in vivo* pretargeting studies with [^225^Ac]DOTA-Bn and synthesis of Pr. A.** As revealed by biodistribution studies in IMR32 tumor-bearing mice (n = 5/tracer), initial efforts to use [^225^Ac]DOTA-Bn for GD2 α-DOTA-PRIT were unsuccessful, leading to low tumor uptake at 24 h p.i. Data is presented as mean ± SD. **B.** Our preliminary *in vivo* pretargeting studies with [^225^Ac]DOTA-Bn indicate that the C825 scFv of the anti-tumor/anti-DOTA BsAb does not bind [^225^Ac]DOTA-Bn (green radioactive circle) with ultra-high affinity, but does bind [^177^Lu]LuDOTA-Bn (yellow radioactive circle).** C.** Schematic of synthesis of proteus-DOTA (Pr), precursor to the radioligands [^225^Ac]Pr (therapy) and [^111^In]Pr (diagnosis). Initially, *p*-SCN-Bn-DOTA is loaded with non-radioactive Lutetium to yield *p*-SCN-Bn-DOTA· Lu^3+^ complex, which is then coupled with NH_2_-PEG-4-DOTA under basic conditions to yield proteus-DOTA (Pr) following purification by RP C-18 HPLC. The ^175^Lu-DOTA-benzene portion of the molecule (yellow circle) is recognized with picomolar affinity by the anti-DOTA C825 scFv domains of the DOTA-PRIT BsAb. The DO3A (green triangle) can be efficiently loaded with ^225^Ac or ^111^In.

As shown in Figure 1A (also see Table S2), initial efforts to pretarget [^225^Ac]DOTA-Bn in IMR-32 tumor-bearing mice led to very low tumor uptake at 24 h p.i. in comparison with [^177^Lu]LuDOTA-Bn at 24 h p.i. (for [^225^Ac]DOTA-Bn and [^177^Lu]LuDOTA-Bn, respectively: 0.49 ± 0.28 %IA/g vs. 10.30 ± 6.42 %IA/g; *P* = 0.0054), prompting the development of Pr. C825 has been shown to have metal specificity 15; e.g., with picomolar binding for DOTA complexes of Lutetium and Yttrium and nanomolar affinity for Indium and Gallium chelates, leading to significant differences in tumor uptake of corresponding radiohapten during *in vivo* pretargeting 16. Therefore, we concluded that the C825 binding of [^225^Ac]DOTA-Bn was unsuitable for efficient *in vivo* α-DOTA-PRIT (Figure 1B).

The chemical synthesis route of Pr is described in Figure 1C. Pr was prepared in very high purity (>98%) and with an overall yield of 34% (please see SI for LC/MS and NMR data). Furthermore, Pr was successfully radiolabeled with ^225^Ac or ^111^In, suggesting that the ^175^Lu-DOTA-benzene moiety of Pr does not interfere with efficient ^225^Ac- or ^111^In-radiometal complexation by the DO3A.

*In vitro* binding experiments with tracer [^225^Ac]Pr and excess BsAb confirmed efficient and stable scFv C825-mediated binding of [^225^Ac]Pr (Figure S2). Efficient tumor targeting *in vivo* was demonstrated using pre-formed BsAb complexed with [^225^Ac]Pr, suggesting that radiolabeled forms of Pr did not interfere with the BsAb anti-tumor antibody domain-tumor antigen interaction (Figure S3).

### *In vivo* studies with radiolabeled Pr

**Figure 2:**
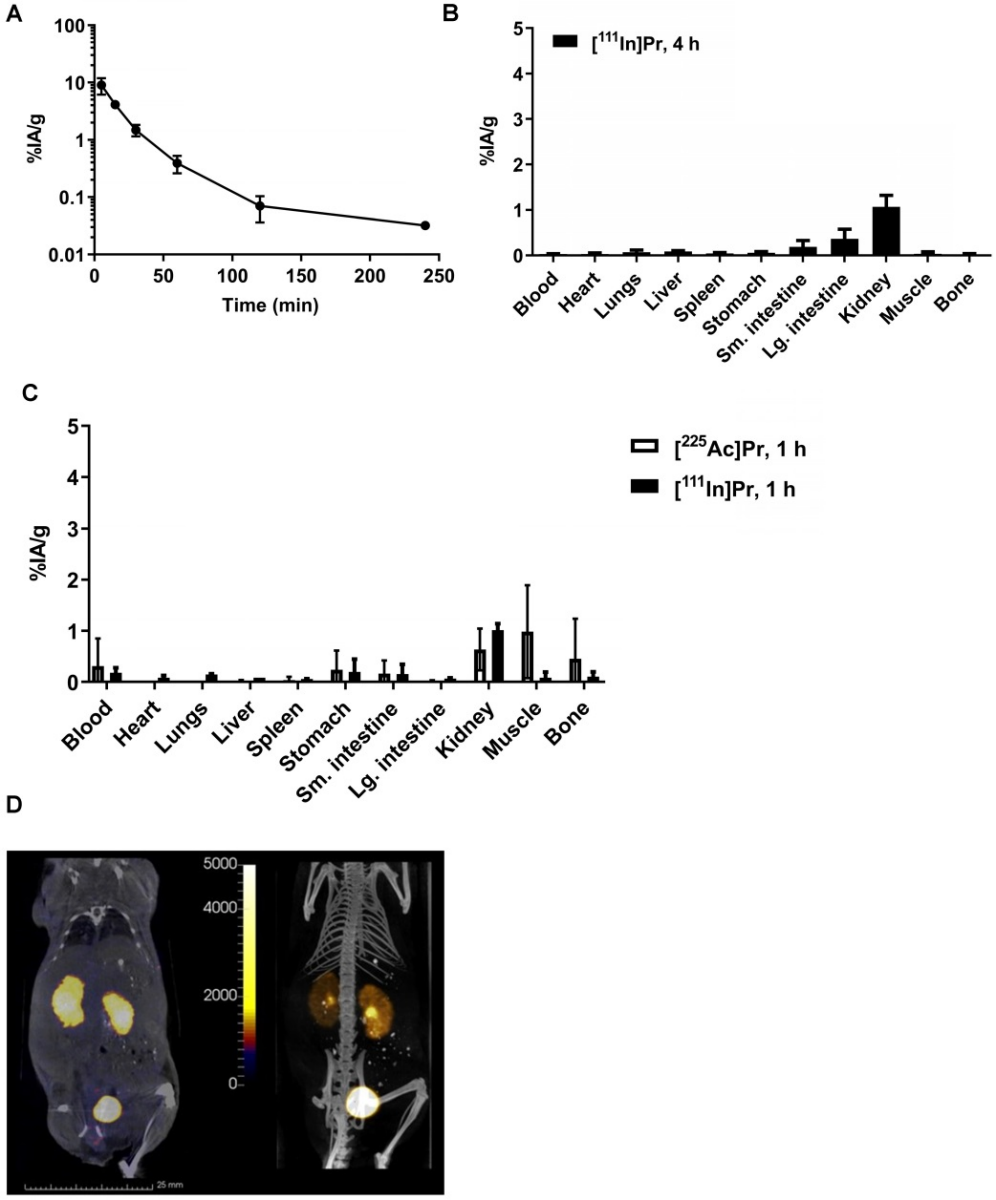
** Pr blood half-life and whole-body clearance. A.** Blood kinetics of [^111^In]Pr in tumor-free athymic nude mice (*n* = 5) and **B.** biodistribution at 240 min (4 h) p.i. (*n* = 5). Data is presented as mean ± SD. The blood half-life of [^111^In]Pr was 7.09 min (fast; 86%) and 24.4 min (slow; 14%) (*R*^2^ = 0.99). Blood clearance parameters were determined by fitting the data to exponential model curves using MATLAB (Mathworks, Inc). **C.** Biodistribution of either [^225^Ac]Pr or [^111^In]Pr in tumor-free athymic nude mice at 1 h p.i. (*n* = 3/tracer). Data is presented as mean ± SD. **D.** SPECT/CT images obtained at 1 h p.i. (*Left*: coronal slice through kidney, SPECT scale units: kBq/mL; *Right*: maximum-intensity projection) confirm renal clearance and minimal normal tissue uptake and retention.

In tumor-free mice, the blood half-life of [^111^In]Pr was 7.09 min (fast; 86%) and 24.4 min (slow; 14%) (*R*^2^ = 0.99) (Figure 2A), consistent with [^177^Lu]LuDOTA-Bn (0.60 min (fast; 92%) and 24.8 min (slow; 8%)) 2. Radiolabeled Pr tracers showed low normal tissue uptake, with the kidney having the highest activity concentration of 1 %IA/g by 1 h p.i. (Figure 2B; see also Table S3). The blood and kidney uptake of [^225^Ac]Pr was 0.31 ± 0.54 %IA/g and 0.63 ± 0.41 %IA/g at 1 h p.i., respectively, consistent with rapid renal clearance and negligible normal tissue uptake. After tissue dissection at 4 h p.i. of [^111^In]Pr, the remaining activity in the carcass was 0.95 ± 0.16 % injected activity (%IA) (note: data not collected for [^225^Ac]Pr).

As revealed in the SPECT/CT images (Figure 2C), in the absence of specific tumor binding of the [^111^In]Pr to pretargeted BsAb, Pr traffics quickly and almost exclusively out of the body by renal clearance. The whole-body activity assays demonstrate rapid excretion of activity with ~90-94% removal by 6 h p.i. of tracer (whole-body clearance half-lives for mouse 1 and mouse 2: 2.71 h (*R*^2^ = 0.912) and 2.03 h (*R*^2^ = 0.992), respectively; Figure S4A). Therefore, the whole-body clearance of [^111^In]Pr is similar to that reported for [^177^Lu]LuDOTA-Bn, as the %IA remaining of [^177^Lu]LuDOTA-Bn in the carcass was reported to be approximately 2-4% at 4 h and 1-3% at 24 h 2. Image-derived volume-of-interest (VOI) analysis of tumor and select normal tissues also confirmed renal clearance and minimal normal tissue uptake and retention (Figures S4B and S4C).

### *In vivo* studies of SW1222 tumor-antigen pretargeted [^225^Ac]Pr or [^111^In]Pr

**Figure 3:**
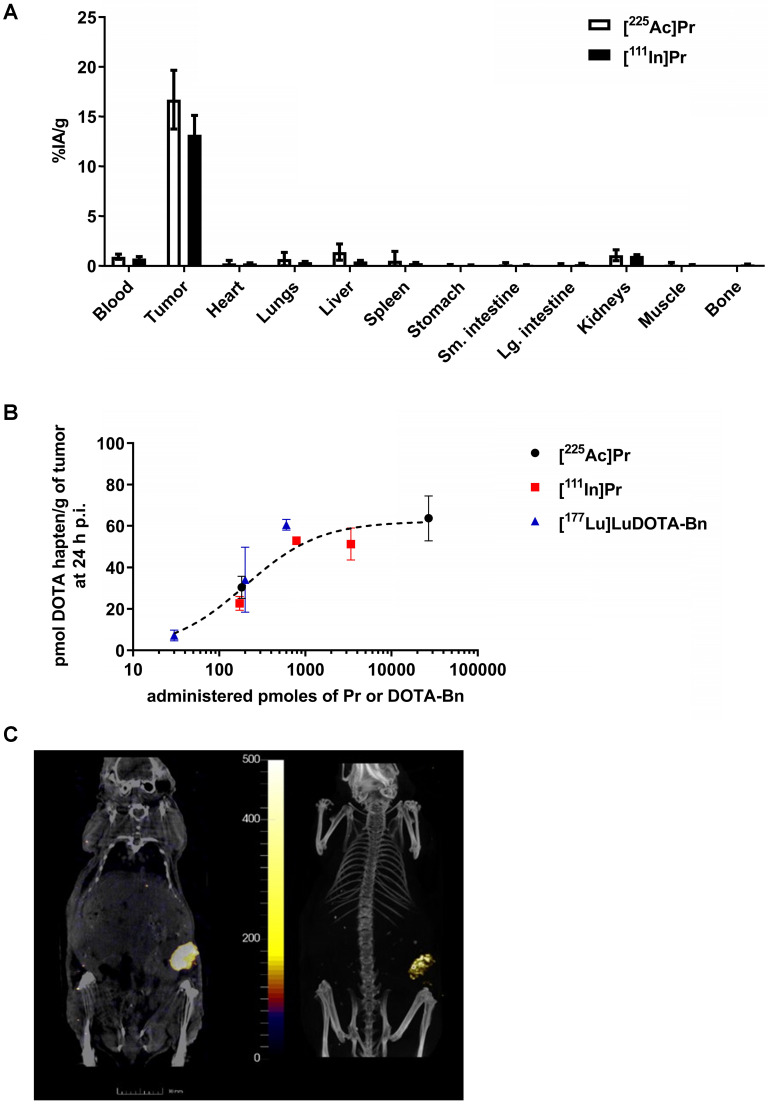
***In vivo* studies of tumor-antigen pretargeted [^225^Ac]Pr or [^111^In]Pr. A.** Comparison of biodistribution of tracer pretargeted [^225^Ac]Pr (*n* = 3) or [^111^In]Pr (*n* = 4) in groups of SW1222 tumor-bearing mice at 24 h p.i. Data is presented as mean ± SD. **B.** Absolute SW1222-tumor uptake of pretargeted radiolabeled DOTA haptens determined using *ex vivo* biodistribution at 24 h p.i., plotted as a function of administered moles of hapten-tracer; Bmax = ~60 pmol radiohapten per gram of SW1222 tumor (*R*^2^ = 0.88). The Bmax was calculated by fitting the data by nonlinear regression analysis (one site, specific binding) using Prism 7 (GraphPad, Inc.). For all data points *n* = 2-4 with the exception of [^111^In]Pr 790 pmol, which is *n* = 1. Data is presented as mean ± SD. Since C825 *does not* bind DOTA-Bn, the molar activity of [^177^Lu]LuDOTA-Bn (~5.45E-3 pmol/kBq or ~0.2 pmol/µCi) was calculated based on the specific activity of the as-received radionuclide (~1,110 GBq/mg or ~30 Ci/mg). On the other hand, since C825 *does* recognize Pr, the molar activity of [^225^Ac]Pr or [^111^In]Pr is calculated based on the moles of added precursor during radiosynthesis as described herein. **C.** SPECT/CT images approximately 24 h p.i. of pretargeted [^111^In]Pr (790 pmol/7.67 MBq) in a SW1222 tumor-bearing mouse (*Left*: coronal slice through tumor, SPECT scale units: kBq/mL; *Right*: maximum-intensity projection). The SW1222-tumor can be clearly delineated in the flank.

The blood, SW1222-tumor, and kidney uptakes of pretargeted [^225^Ac]Pr at 24 h p.i. were 0.94 ± 0.37 %IA/g, 16.71 ± 5.11 %IA/g, and 1.08 ± 0.95 %IA/g, respectively, corresponding to tumor-to-organ activity ratios (T:NT) of about 18:1 and 16:1 for blood and kidney, respectively (Figure 3A and Table S4). The lowest T:NT was observed for liver (~12:1), with liver uptake of 1.40 ± 1.42 %IA/g). For [^111^In]Pr, the blood, tumor, and kidney uptakes at 24 h p.i. were 0.76 ± 0.38 %IA/g, 13.19 ± 3.88 %IA/g, and 1.02 ± 0.38 %IA/g, respectively, corresponding to T:NT of approximately 17:1 and 13:1 for blood and kidney, respectively.

No major differences in the biodistribution profiles were seen between pretargeted [^225^Ac]Pr or [^111^In]Pr with two exceptions (Table S5): liver, which on average was about three times higher for [^225^Ac]Pr (1.40 ± 1.42 %IA/g versus 0.47 ± 0.20 %IA/g for [^225^Ac]Pr or [^111^In]Pr, respectively; *P* = 0.066, not significant), and bone, which was higher for [^111^In]Pr (not detectable above background versus 0.16 ± 0.06 %IA/g for [^225^Ac]Pr or [^111^In]Pr, respectively). We found no evidence of hepatic damage, however, on immunohistochemistry (IHC) of mice injected with [^225^Ac]Pr (Tables S6 and S11). Biodistribution analysis at 24 h p.i. following *in vivo* competition studies showed maximum tumor uptake (“Bmax”) of ~60 pmol radiohapten per gram of SW1222 tumor ((Bmax = 62.23, *R*^2^ = 0.88; Figure 3B), as well as verified that pretargeting of Pr to tumor is similar to [^177^Lu]LuDOTA-Bn. These data will serve as a guide for therapeutic dosing of radiolabeled forms of Pr, as maximizing the relative uptake of tumor to normal tissues is essential for high-TI targeting.

Finally, highly efficient tumor pretargeting of [^111^In]Pr was also verified by non-invasive SPECT/CT imaging, as SW1222-tumor can be clearly delineated in the flank at 20 h p.i. (Figure 3C). Image-based VOI (mean) analysis of tumor, kidney (left), heart, and liver revealed activity concentrations of 6.89 ± 4.68 %IA/g, 0.46 ± 0.47 %IA/g, 0.20 ± 0.24 %IA/g, and 0.22 ± 0.27 %IA/g, respectively. In addition, the average activity concentration for the highest 10% of voxels in each VOI were also tabulated; for tumor: 18.48 ± 2.92 %IA/g; kidney (left): 1.80 ± 0.79 %IA/g; heart: 0.91 ± 0.46 %IA/g; and liver 1.03 ± 0.48 %IA/g.

### Therapy with α-DOTA-PRIT

**Figure 4:**
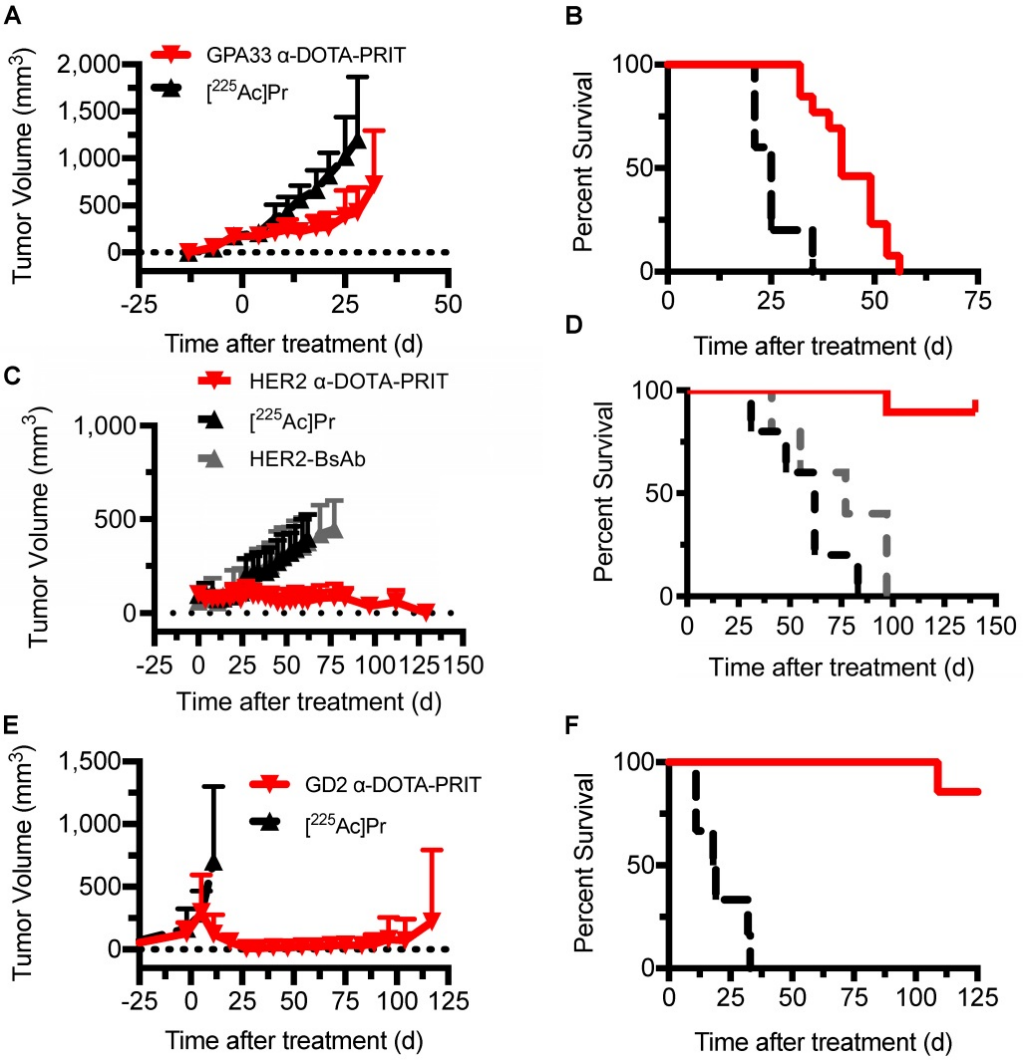
** Single-cycle therapeutic efficacy of GPA33 α-DOTA-PRIT in SW1222 tumor-bearing mice (*n* = 5-13), GD2 α-DOTA-PRIT in IMR-32 tumor-bearing mice (*n* = 6-7), and HER2 α-DOTA-PRIT in BT-474 tumor-bearing mice (*n* = 5-19). Tumor volumes represent mean ± SD. A.** Mean SW1222 tumor volumes in mice that received GPA33 α-DOTA-PRIT (296 kBq) in comparison to [^225^Ac]Pr alone (296 kBq) (*n* = 13 and *n* = 5, respectively). **B.** Survival analysis for GPA33 α-DOTA-PRIT. MS (in d post-treatment) was 42 d vs. 25 d for GPA33 α-DOTA-PRIT and [^225^Ac]Pr only, respectively (*P* < 0.0001). **C.** Mean BT-474 tumor volumes in mice that received HER2 α-DOTA-PRIT (296 kBq) in comparison to [^225^Ac]Pr alone (296 kBq) or BsAb alone (*n* = 19, *n* = 5, and *n* = 5, respectively). **D.** Survival analysis for HER2 α-DOTA-PRIT. MS (in d after treatment start) was >130 d for HER2 α-DOTA-PRIT vs. 62 d or 77 d for [^225^Ac]Pr or BsAb only, respectively (*P* < 0.0001). **E.** Mean IMR-32 tumor volumes in mice that received anti-GD2 α-DOTA-PRIT (37 kBq) in comparison to [^225^Ac]Pr alone (37 kBq) (*n* = 7 and *n* = 6, respectively). **F.** Survival analysis for GD2 α-DOTA-PRIT. MS (in d after treatment start) was significantly different (i.e., >125 d) for GD2 α-DOTA-PRIT, compared to 18.5 d post-treatment for [^225^Ac]Pr-only treatment controls (*P* = 0.0002).

Single-cycle therapy was performed in the three tumor xenograft models for initial testing of efficacy and safety. There were no significant differences in tumor volumes at time of treatment initiation between treatment groups and [^225^Ac]Pr alone controls. Briefly, for SW1222: [^225^Ac]Pr alone (*n* = 5; 176 ± 24 mm^3^, range: 136-200 mm^3^) *versus* GPA33 α-DOTA-PRIT therapy (*n* = 13; 171 ± 32 mm^3^, range: 144-248 mm^3^), *P* = 0.375; for BT-474: [^225^Ac]Pr alone (*n* = 5; 108 ± 57 mm^3^, range: 66-208 mm^3^) *versus* HER2 α-DOTA-PRIT therapy (*n* = 19; 98 ± 39 mm^3^, range: 49-212 mm^3^), *P* = 0.322; for IMR32/luc: [^225^Ac]Pr alone (*n* = 6; 234 ± 176 mm^3^, range: 69-480 mm^3^) *versus* GD2 α-DOTA-PRIT therapy (*n* = 7; 319 ± 278 mm^3^, range: 31-666 mm^3^), *P* = 0.266. Also, for BT-474, an additional control was included (treatment with BsAb only), and the tumors were significantly smaller (for BsAb only: *n* = 5; 63 ± 14 mm^3^, range: 40-74 mm^3^ versus HER2 α-DOTA-PRIT therapy (*n* = 19; 98 ± 39 mm^3^, range: 49-212 mm^3^), *P* = 0.032. In SW1222 tumor-bearing animals (tumor volumes at treatment start: 141-248 mm^3^), GPA33 α-DOTA-PRIT significantly slowed tumor progression and extended survival compared with mice treated with [^225^Ac]Pr only (Figure 4A and 4B). Median survival (MS; in d post-treatment) was 42 d vs. 25 d for GPA33 α-DOTA-PRIT and [^225^Ac]Pr only, respectively (*P* < 0.0001). In BT-474 tumor-bearing animals (tumor volumes at treatment start: 40-212 mm^3^), HER2 α-DOTA-PRIT led to 7/19 (37%) complete response (CR) and significantly extended survival compared with mice treated with controls (Figure 4C and 4D). Furthermore, 3/19 HER2 α-DOTA-PRIT treated mice died within 150 d post-treatment with tumor volumes of 141 mm^3^ (day 97), 67 mm^3^ (day 97), and no tumor (CR; died on day 140, not assessable by pathology). The remaining 16 mice (6 with CR, others with palpable mass) had pathologic examinations of tumor at study endpoint and 6/6 CRs were confirmed to be tumor-free (i.e., histologic cure). Of the 10 mice with palpable mass at site of tumor inoculation, 4/10 were histologic cures, 4/10 had small nests of neoplastic cells but no tumor, and 2/10 had residual tumor. In summary, of 16/19 assessable mice: 10/16 were cured; 4/16 had small nests; and 2/16 had residual tumor. MS (in d after treatment start) was >130 d for HER2 α-DOTA-PRIT vs. 62 d or 77 d for [^225^Ac]Pr or BsAb only, respectively (*P* < 0.0001). Notably, MS was not significantly different for treatment with [^225^Ac]Pr versus treatment with BsAb only (*P* = 0.2501). IMR-32/luc tumor-bearing animals (tumor volumes at treatment start: 31-605 mm^3^) treated with GD2 α-DOTA-PRIT led to 100% CR within ~30 d of treatment start with eventual tumor escape in 3/7 treated mice, while treatment with [^225^Ac]Pr was ineffective, with 100% of mice requiring sacrifice due to tumor progression ~30 d of treatment (Figure 4E). We observed BLI-positive (tumor volumes: 74 mm^3^ and 474 mm^3^) in 2/7 animals within 90 d of treatment (Figure S5). Of these two BLI-positive mice, one required sacrifice due to tumor burden at 117 d after treatment, and the other was sacrificed for further analysis after the tumor failed to grow above 100 mm^3^ within 210 d of treatment. We have observed this rare apparent spontaneous tumor regression previously during treatment studies of SW1222 with β-DOTA-PRIT (^177^Lu) 19, and we are currently investigating the possibility of a senescent phenotype. A third mouse developed a recurrence and had to be sacrificed 133 d after treatment due to tumor burden. The remaining four mice were confirmed to be histologic cures 141-241 d after treatment. In summary, of 4/7 assessable mice: 4/4 were cured. Survival of mice treated with GD2 α-DOTA-PRIT compared with controls was significantly extended (Figure 4F); the MS (in d after treatment start) was significantly different (i.e., >125 d) for GD2 α-DOTA-PRIT, compared to 18.5 d post-treatment for [^225^Ac]Pr-only treatment controls (*P* = 0.0002).

### Toxicity of [^225^Ac]Pr and α-DOTA-PRIT

**Figure 5:**
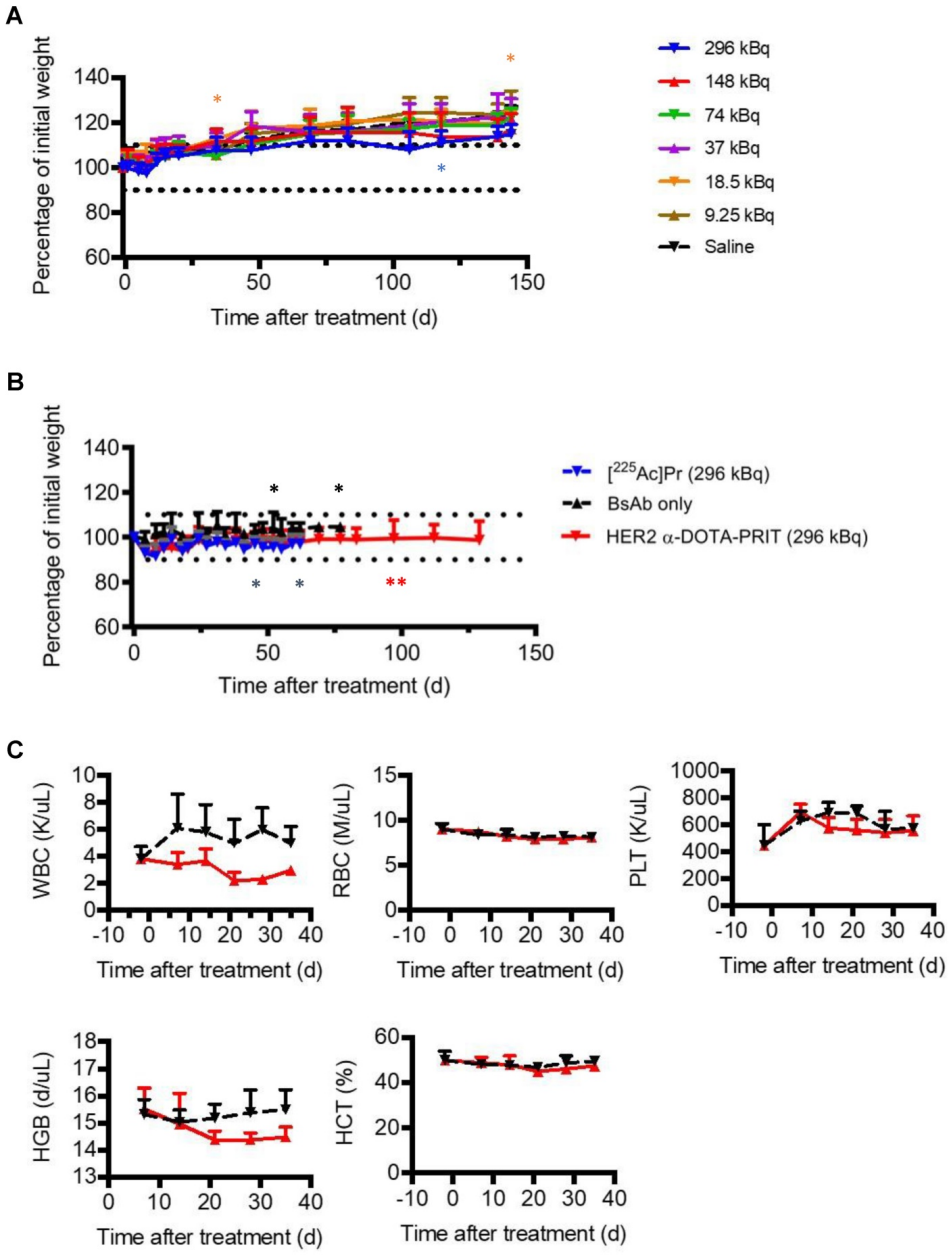
** Toxicity evaluation after treatment with [^225^Ac]Pr (up to 296 kBq) or HER2 α-DOTA-PRIT (296 kBq). A.** Body weight progression of groups of healthy nude mice treated with [^225^Ac]Pr (*n* = 5/dose). The MTD was not reached. Treated animal weights plotted as the percentage of pre-treatment baseline weight. The asterisk (*) indicates that the mouse required euthanasia or was discovered deceased (3/35, 9%). Data represent mean ± SD. **B.** Body weight progression of groups from HER2 α-DOTA-PRIT (296 kBq) study. BT-474 tumor-bearing female athymic nude mice were treated with HER2 α-DOTA-PRIT (296 kBq) or controls ([^225^Ac]Pr only or BsAb only) and evaluated for toxicity. Data represent mean ± SD. For [^225^Ac]Pr only, *n* = 5; for BsAb only *n* = 5; and for HER2 α-DOTA-PRIT, *n* = 19. Treated animal weights are plotted as the percentage of pre-treatment baseline weight. For clarity, data is shown until MS (62 d or 77 d for HER2 α-DOTA-PRIT and [^225^Ac]Pr or BsAb only, respectively). The asterisk (*) indicates that the mouse required euthanasia due to tumor burden (controls) or apparent toxicity (HER2 α-DOTA-PRIT). **C.** Select hematology data from groups of BT474-tumored mice undergoing treatment with HER2 α-DOTA-PRIT (296 kBq) (red line) or no treatment control (black line); *n* = 4 for baseline at -2 days and *n* = 5 for all other data. Data represent mean ± SD. WBC, white blood cells; RBC, red blood cells; PLT, platelets; HBG, hemoglobin; HCT, hematocrit.

Prior studies with long circulating ^225^Ac-IgG provide a valuable benchmark for toxicity comparisons to the target organ (kidney) for ^225^Ac toxicity after parenteral injection in mice 28. For example, radiation nephropathy was observed in ^225^Ac-IgG (720 kBq/kg (12.95 kBq))-treated mice examined at 20 w (from 28, summarized in Table 1). In contrast, in our study, no acute toxicity (as weight loss >10% of baseline; Figure 5A), nor any radiation-induced histologic organ damage was observed at any [^225^Ac]Pr dose level at necropsy performed at 145 d (Table 1 and Table S6). Additional details are provided in SI.

Treatment studies with either: GPA33 α-DOTA-PRIT, GD2 α-DOTA-PRIT, or [^225^Ac]Pr only was well tolerated, with no unscheduled mortalities. Treatment with HER2 α-DOTA-PRIT was also well tolerated, but three unscheduled mortalities were noted (3/19, 16%; two mice at 97 d and a single mouse at 140 d after treatment), which may be related to estrogen supplementation in the BT-474 mouse model, which was observed during HER2 β-DOTA-PRIT 20). No weight losses were recorded in any of the treatment groups (SW1222: Figure S7; BT-474: Figure 5B; IMR-32/luc: Figure S8). Complete blood count (CBC) measurements in the mice treated with GPA33 α-DOTA-PRIT (Figure S9) or HER2 α-DOTA-PRIT (Figure 5C) revealed no significant changes during the observation period (up to 36 d post-treatment), with the exception of WBC for HER2 α-DOTA-PRIT, which showed an average ~55% difference at 21 d post-treatment (3.82 K/uL versus 2.21 K/uL for -2 d and 21 d post-treatment, respectively; *P* = 0.00813), with rebound to average 2.97 K/uL at 36 d (~25% difference from -2 d post-treatment, *P* = 0.04488).

**Figure 6:**
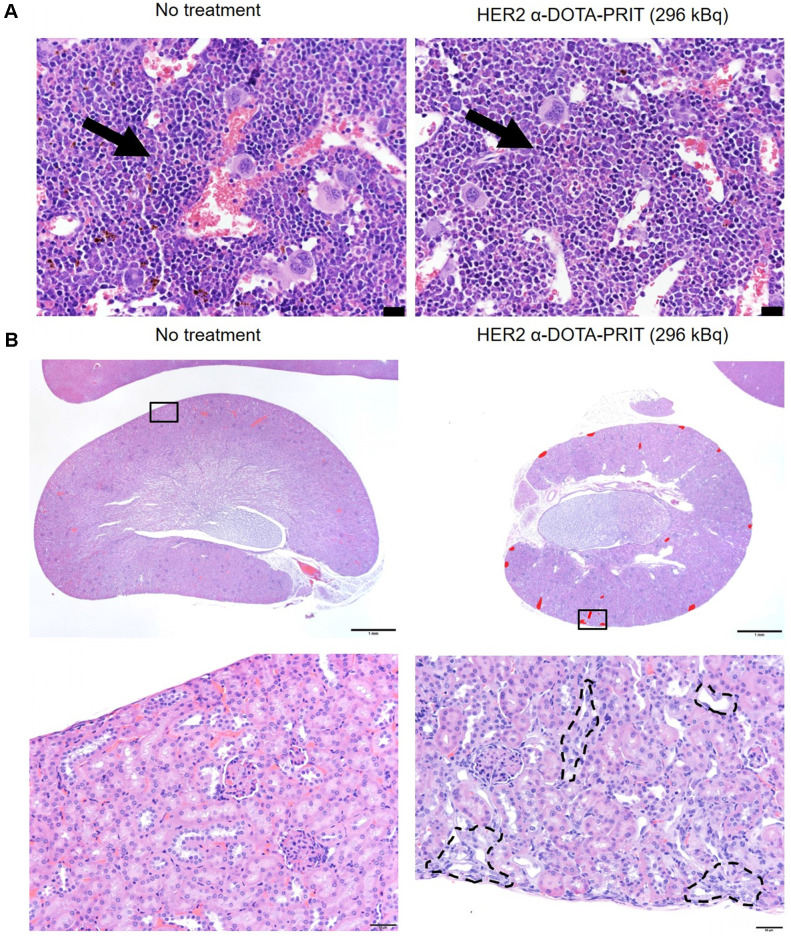
** Representative histology of bone marrow and kidney of BT-474 tumor-bearing female athymic nude mice treated with HER2 α-DOTA-PRIT (296 kBq) or no treatment control at 150 d. A.** H&E staining of bone marrow. The bone marrow in the images is from sternum (but we examined bone marrow in sternum, femur, tibia, and vertebrae in all mice and all sites were normal). Representative hematopoietic cells have been labeled with arrows. There is no difference in cellularity (i.e. cell density) of hematopoietic cells. Scale bar = 20 µm. **B.** H&E staining of kidney. Lesions are labeled in red on the low magnification image. Histopathology of the kidneys revealed multifocal minimal to mild chronic tubular injury in treated mice, while the kidneys of the untreated animal was histologically normal. While these lesions were interpreted as probably caused by the treatment, they were not considered adverse, as they did not appear to significantly affect renal function or general health of the animals, based on the low percentage of the renal parenchyma affected (< 5%), normal serum CREA concentration and minimal elevation of BUN, and absence of clinical signs or change in body weight. Scale bars = 1000 µm (low magnification) and 50 µm (high magnification).

In groups treated with HER2 α-DOTA-PRIT (296 kBq), no changes in gross organ weights, apart from a moderate decrease in kidney weights (Figure S6B), nor radiation-induced histologic organ damage was observed at necropsy performed at 150 d (Figure 6 and Table S11). There was evidence of minimal to mild renal tubular injury and such changes may be observed spontaneously in aging mice, so we cannot definitively attribute this finding to treatment, although the group difference suggests a possible association with treatment. Remarkable tubuleinterstitial features included minimal to mild multifocal cortical tubular degeneration and atrophy in 5/6 (83%) treated animals. Tubulointerstitial features were all <5% (Table 1). The CBC showed no significant group differences or deviation from reference ranges (Tables S12-14). A single significant effect on serum chemistry (Table S15) that could be attributed to treatment consisted of evidence of mild elevation of serum blood urea nitrogen (BUN) and creatinine (CREA) in the mice showing minimal to mild renal tubular injury. BUN was occasionally slightly higher than the reference range while the CREA was always within the reference ranges despite the slight increase compared to untreated mice.

Histopathology analysis of the survivors from the GD2 α-DOTA-PRIT (37 kBq) study at 141-241 d revealed no treatment-related findings (Table S16). The hematology and clinical chemistry values were also within normal reference limits (Tables S17-S19). The elevated aspartate transaminase in a single treated mouse had no histopathologic correlate and was considered incidental (Mouse #4; Table S19).

## Discussion

α-radiation is potentially one of the most highly focused and radiotoxic anti-cancer agents known, wherein a few radioactive atoms can kill a cancer cell. α-particles have much greater compared to β-radiation, due to their high energy deposition (α-particles: 5-8 MeV; β-particles: 0.1-1 MeV) and far shorter penetration depth (α-particles: 50-80 µm, corresponding to 2-4 typical cell diameters; β-particles: 2-12 mm) 35. Thus, α-emitters offer high-LET radiation with multiple lethal radiation events, potentially leading to a significant number of double DNA strand breaks. In addition, high selectivity is achieved once they are cancer cell-bound, which results in minimal collateral damage to normal tissue 35.

α-RIT with whole IgG combines highly cytotoxic α radiation with efficient tumor targeting. However, the combination of slow tumor uptake and whole-body clearance creates off-target radiotoxicities. ^225^Ac decay results in 4 α-particles, each of which are associated with daughter radionuclides, which can be redistributed throughout the body via the circulation. Radiation nephropathy was reported in mice at administered activities of [^225^Ac]-IgG, ranging from 12.95-14.80 kBq/mouse 28, 36, 37; 37 kBq/mouse was 100% lethal 38. As an alternative to IgG with long biologic half-lives, small-size carriers with short serum half-lives such as peptides should theoretically shorten radiation exposure, but the renal uptake of such peptides can lead to significant whole-body retention of 10-30% injected dose 2, accompanied by minor (e.g., negligible protein cases observed histologically) to major kidney damage (e.g, pathologic changes consistent with radiation-induced tubular necrosis observed) based on the administered ^225^Ac-activity 39-41.

Previous manifestations of PRIT were investigated clinically with objective tumor response including CRs (e.g., strept(avidin)-biotin PRIT with Yttrium-90 for treatment of non-Hodgkin's lymphoma 42), and proof of principle for targeting human tumors in patients was achieved. Actually, for a single dose, very good targeting was achieved to tumors at doses of antibody and radioactivity, which were in a practical range. Nonetheless, using these reagents, full success including cures was essentially out of reach due to a combination of the immunogenicity of strept(avidin), the complexity of dosing schedules, and TI, including renal dose due to retention of strept(avidin). It was clear at that time that achieving radiation doses necessary to effectively treat solid tumors would be associated with intolerable toxicity, especially to kidneys. Fundamental improvement in reagents for PRIT, such as ultra-high affinity multivalent BsAb-based approaches to PRIT, including DOTA-PRIT 12, 15, generated new enthusiasm for translation due to the potential for reduced immunogenicity via use of humanized BsAb and absence of endogenous biotin interference, as well as superior TI and efficacy to prior versions of PRIT 21, 43.

α-DOTA-PRIT offers a potential solution: an anti-tumor antibody-based drug platform that harnesses radiohapten capture and glomerular filtration to significantly enhance therapeutic window and safety, possibly allowing dose escalation to cure for human tumors. Thus, we reasoned that this Pr chelator cage, with a MW of ~1.5 kDa (as [^225^Ac]Pr or [^111^In]Pr for pretargeting ^225^Ac or ^111^In, respectively), would have suitable uptake in tumor and clearances ideal for high-TI DOTA-PRIT. Furthermore, this Pr approach should, in principle, allow for highly efficient and specific α-particle RIT, irrespective of the tumor antigen or tumor type, a true “plug-and-play” platform.

In the current publication, we show that at radionuclide doses up to 296 kBq [^225^Ac]Pr per animal, or 23 times the administered activity of [^225^Ac]-IgG observed to be radiotoxic to kidney, there is no detectable toxicity at 140 d after injection. MTD is not reached for [^225^Ac]Pr and, in fact, there is no detectable histologic evidence of adverse radiation effects. Using anti-GPA33 DOTA-PRIT as a model system, we showed that [^225^Ac]Pr or [^111^In]Pr could be pretargeted *in vivo* to GPA33-expressing human colorectal cancer SW1222 xenografts with radioactive uptakes in the range of 13-17 %IA/g for tumor, and with liver showing modest radioactive uptake for [^225^Ac]Pr (~1.5 %IA/g) and kidney (~1.0 %IA/g) for [^111^In]Pr. These activities in tumor and normal tissues resulted in T:NT with both haptens of ≥~12:1 at 24 h p.i. of tracers, consistent with previous studies in SW1222 tumor-bearing mice with GPA33 β-DOTA-PRIT (T:NT ≥~18:1 at 24 h p.i., with ~10 %IA/g for tumor) 19. These tissue uptake ratios translate into high TI and provide a basis for potentially curative regimens for both α- and β-emitters.

The purpose of this study was to introduce a new DOTA-PRIT hapten for α-PRIT that allows us to obtain significant tumor responses, including CRs and cures, without toxicity. Furthermore, while we establish therapeutic efficacy without observing adverse effects, these initial studies provide benchmarks for further optimization of α-DOTA-PRIT, including: (1) improved tracer dosing with higher specific or molar activity preparations of [^225^Ac]Pr to balance tumor saturation with normal tissue uptake; (2) dosimetry studies to establish dose-response correlates and account for the complexity of the ^225^Ac-decay chain (i.e., *in vivo* fate of the recoiled daughter isotopes on α-emission), as well as the intra-cellular residence of [^225^Ac]Pr; (3) utilize protein engineering innovations to simplify dosing; and (4) utilize fractionated treatment regimens to potentially improve therapeutic outcome. We consider these important experiments to be outside the scope of the current manuscript.

Using DOTA-PRIT for tumor targeting of [^225^Ac]Pr in models of human colon cancer, breast cancer, or neuroblastoma, tested activities ranging from 37-296 kBq of ^225^Ac/mouse were well tolerated, while tumors were ablated. α-DOTA-PRIT therapy was not limited by acute or chronic radiotoxicity to normal organs. We demonstrated that GPA33 α-DOTA-PRIT was effective in SW1222 tumor-bearing mice, observing marginal but significant therapeutic effects (tumor growth control with prolonged survival; *P*<0.0001; Mantel-Cox test), with no detectable hematopoietic toxicity. Tumor response was likely limited by a combination of factors including the non-internalizing huA33 BsAb-GPA33 antibody-antigen complex 44 and low [^225^Ac]Pr specific activity, significantly impacting tumor localization of decay daughters and absolute tumor uptake of ^225^Ac, respectively. Furthermore, treatment of solid tumors with α-particle RIT, including α-DOTA-PRIT, may be theoretically limited by the potential mismatch between limited antibody penetration in tumor 45 and the short penetration depth of α-particles, leading to intratumoral absorbed dose heterogeneity.

Although not a requirement for efficacy, the application of internalizing mAb for improved α-RIT potency has been demonstrated 46, as there is a limited probability of α-particles originating on the cell surface to traverse a cell due to the high recoil energy of α-particle emission and daughter product release 47. We show that using an internalizing HER2 BsAb for treatment of BT-474 tumor-bearing mice, we can achieve 7/19 (37%) CRs, including 10/19 (53%) with verified histologic cure at ~150 d post-treatment. This result was somewhat surprising, considering that we anticipated that the low molar activity of [^225^Ac]Pr would potentially impair responses as we observed for GPA33 α-DOTA-PRIT. We cautiously attribute this high level of efficacy to a combination of factors, including increased probability of exposure of tumor to α-particles due to increased internalization 48, as well as possibly higher radiosensitivity compared with SW1222 based on our previous β-DOTA-PRIT studies. We establish benchmarks for kidney toxicity, as remarkable tubulointerstitial features included minimal to mild multifocal cortical tubular degeneration and atrophy in 5/6 (83%) HER2 α-DOTA-PRIT (296 kBq)-treated animals. These lesions were interpreted as probably caused by the treatment, but they were not considered adverse, as they did not appear to significantly affect renal function or general health of the animals, based on the low percentage of the renal parenchyma affected (< 5%), normal serum CREA concentration and minimal elevation of BUN, and absence of clinical signs or change in body weight.

Also, we demonstrate in IMR-32 tumor-bearing mice that 100% of CRs could be achieved with a single pretargeted administered activity of 37 kBq, including 4/7 showing histologic cure at 141-241 d post-treatment. We hypothesize this remarkable efficacy to the relative radio-sensitivity of the model to [^225^Ac]Pr treatment, even though the hu3F8 BsAb-GD2 antibody-antigen complex is minimally internalizing 38, 49. No treatment-related histopathologic changes were noted in 4/4 (100%) GD2 α-DOTA-PRIT (37 kBq)-treated animals.

In addition to therapy applications, the ability to readily image tumor with high contrast to normal tissue is significant for staging as well as dosimetry. The development of theranostic pairs for patient-specific dosimetry has been described as imperative for precision molecular radiotherapy (e.g., radiotherapy of prostate-specific membrane antigen using α- and β-emitters 50). As a theranostic pair for ^90^Y or ^177^Lu β-DOTA-PRIT, ^86^Y can also be used for positron emission tomography (PET) 19. As a theranostic pair for [^225^Ac]Pr, ^111^In is more convenient due to its considerably lower cost and availability (compared to ^86^Y), as well as its acceptance in routine nuclear medicine SPECT imaging, which is still more widely available than PET. In addition, ^111^In has a half-life of 2.8 d, a better match to the half-life of ^225^Ac (10 d) in comparison to ^86^Y (14 h), for the purposes of dosimetry estimates. The use of ^111^In as a surrogate for ^225^Ac was previously described in an antibody-oligonucleotide/radiolabeled oligonucleotide PRIT system by Mulvey, et al. 51. We demonstrate that [^111^In]Pr is a suitable SPECT imaging surrogate for [^225^Ac]Pr for dosimetry and treatment monitoring, with comparably high TI and low uptake in the key normal tissues, kidney, liver, and bone. Thus, as a theranostic radiohapten, [^111^In]Pr will help establish optimized α-therapy regimens centered on quantitative dosimetry, emphasizing cures with minimal toxicity in target organs.

## Conclusion

In summary, we have described a novel proteus-DOTA that can efficiently chelate ^225^Ac for use as a small radioligand for DOTA-PRIT. In addition, we have developed [^111^In]Pr as a SPECT-imaging surrogate for [^225^Ac]Pr for dosimetry and treatment monitoring. Finally, we have established therapeutic activity of α-DOTA-PRIT in models of human colon cancer, breast cancer, or neuroblastoma. Furthermore, these anti-tumor effects were comparable to those achieved with β-DOTA-PRIT, although with much lower levels of administered [^177^Lu]LuDOTA-Bn radioactivity (for GPA33: 55 MBq/mouse 19; for HER2: 167 MBq/mouse 20; for GD2: 33 MBq/mouse 17), consistent with recently reported preclinical α-therapy studies 41 including α-PRIT 52, 53. With pharmacodynamic control of radiotoxic non-tumor-bound [^225^Ac]Pr, DOTA-PRIT offers a meaningful improvement in the selectivity of α-therapy.

## Supplementary Material

Supplementary figures and tables.

## Figures and Tables

**Table 1:** Radiation-induced morphologic changes in mouse kidneys 20 w after injection with either: ^225^Ac-labeled antibody ([^225^Ac]-IgG), [^225^Ac]Pr, or HER2 α-DOTA-PRIT

Renal tubulointerstitial features	[^225^Ac]-IgG 12.95 kBq/mouse^*^	[^225^Ac]Pr 296 kBq/mouse	HER2 α-DOTA-PRIT 296 kBq/mouse
Abnormal/reactive nuclear change	Moderate, focal karyorrhexis	Mild, focal karyomegaly	Mild, focal karyomegaly and karyorrhexis
Cytoplasmic vacuolization (% of cells)	>50	<1	<5
Tubulolysis with collapse (% of tubules)	>50	<1	<5
Loss of brush border (% of tubules)	70	<1	<5
Atrophy (% of tubules)	---	<1	<5
Shrinkage/simplification (% of residual tubules)	50	<1	<5
Interstitial inflammation/interstitial fibrosis	---/---	None	Minimal, lymphoplasmacytic interstitial inflammation
Medullary tubules	Normal	Normal	Normal

*From Jaggi et al. 28: female BALB/c mice, 6 weeks old and weighing 18-20 g were administered 12.95 kBq of [^225^Ac]HuM195 ([^225^Ac]-IgG; HuM195 is a humanized IgG1 that recognizes the extracellular domain on CD33).

## Abbreviations

PRIT: pretargeted radioimmunotherapy
TI: therapeutic index
RIT: radioimmunotherapy
BsAb: bispecific anti-tumor/anti-DOTA-hapten antibody
DOTA: 1,4,7,10-tetraazacyclododecane-N, N', N'', N'''-tetraacetic acid
scFv: single-chain variable fragment
CA: clearing agent
MW: molecular weight
Pr: proteus-DOTA
SI: supplemental information
SPECT/CT: single-photon emission computed tomography/computed tomography
i.v.: intravenous
NSS: normal sterile isotonic saline solution
%IA/g: percent injected activity per gram
p.i.: post-injection
DO3A: three-arm DOTA radiometal-chelating agent
RP: reverse-phase
HPLC: high-pressure liquid chromatography
LC/MS: liquid-chromatography/mass spectrometry
NMR: nuclear magnetic resonance
RCY: radiochemical yield
RCP: radiochemical purity
EDTA: ethylenediaminetetraacetic acid
MTD: maximum tolerated dose
RBE: relative biologic effectiveness
LET: linear energy transfer
BLI: bioluminescent imaging
WBC: white blood cell
NE: neutrophils
LY: lymphocytes
MO: monocytes
EO: eosinophils
BA: basophils
RBC: red blood cell
HGB: hemoglobin
HCT: hematocrit
MCV: mean corpuscular volume
MCH: mean corpuscular hemoglobin
MCHC: mean corpuscular hemoglobin concentration
RDW: red cell distribution width
PLT: platelets
MPV: mean platelet volume
SD: standard deviation
%IA: percent injected activity
VOI: volume of interest
T:NT: tumor-to-organ activity ratios
IHC: immunohistochemistry
MS: median survival
CR: complete response
CBC: complete blood count
BUN: blood urea nitrogen
CREA: creatinine
PET: positron emission tomography

## References

[1] Wittrup KD, Thurber GM, Schmidt MM, Rhoden JJ (2012). Practical theoretic guidance for the design of tumor-targeting agents. Methods Enzymol.

[2] Orcutt KD, Nasr KA, Whitehead DG, Frangioni JV, Wittrup KD (2011). Biodistribution and clearance of small molecule hapten chelates for pretargeted radioimmunotherapy. Mol Imaging Biol.

[3] Larson SM, Carrasquillo JA, Cheung NK, Press OW (2015). Radioimmunotherapy of human tumours. Nat Rev Cancer.

[4] Hapuarachchige S, Huang CT, Donnelly MC, Barinka C, Lupold SE, Pomper MG (2020). Cellular Delivery of Bioorthogonal Pretargeting Therapeutics in PSMA-Positive Prostate Cancer. Mol Pharm.

[5] Verhoeven M, Seimbille Y, Dalm SU (2019). Therapeutic Applications of Pretargeting. Pharmaceutics.

[6] Liu G (2018). A Revisit to the Pretargeting Concept-A Target Conversion. Front Pharmacol.

[7] Strebl MG, Yang J, Isaacs L, Hooker JM (2018). Adamantane/Cucurbituril: A Potential Pretargeted Imaging Strategy in Immuno-PET. Mol Imaging.

[8] Altai M, Membreno R, Cook B, Tolmachev V, Zeglis BM (2017). Pretargeted Imaging and Therapy. J Nucl Med.

[9] Frampas E, Rousseau C, Bodet-Milin C, Barbet J, Chatal JF, Kraeber-Bodere F (2013). Improvement of radioimmunotherapy using pretargeting. Front Oncol.

[10] Goldenberg DM, Chang CH, Rossi EA (2012). Pretargeted molecular imaging and radioimmunotherapy. Theranostics.

[11] Boerman OC, van Schaijk FG, Oyen WJ, Corstens FH (2003). Pretargeted radioimmunotherapy of cancer: progress step by step. J Nucl Med.

[12] Orcutt KD, Ackerman ME, Cieslewicz M, Quiroz E, Slusarczyk AL, Frangioni JV (2010). A modular IgG-scFv bispecific antibody topology. Protein Eng Des Sel.

[13] Corneillie TM, Fisher AJ, Meares CF (2003). Crystal structures of two complexes of the rare-earth-DOTA-binding antibody 2D12.5: ligand generality from a chiral system. J Am Chem Soc.

[14] Corneillie TM, Whetstone PA, Fisher AJ, Meares CF (2003). A rare earth-DOTA-binding antibody: probe properties and binding affinity across the lanthanide series. J Am Chem Soc.

[15] Orcutt KD, Slusarczyk AL, Cieslewicz M, Ruiz-Yi B, Bhushan KR, Frangioni JV (2011). Engineering an antibody with picomolar affinity to DOTA chelates of multiple radionuclides for pretargeted radioimmunotherapy and imaging. Nucl Med Biol.

[16] Orcutt KD, Rhoden JJ, Ruiz-Yi B, Frangioni JV, Wittrup KD (2012). Effect of small-molecule-binding affinity on tumor uptake *in vivo*: a systematic study using a pretargeted bispecific antibody. Mol Cancer Ther.

[17] Cheal SM, Xu H, Guo HF, Zanzonico PB, Larson SM, Cheung NK (2014). Preclinical evaluation of multistep targeting of diasialoganglioside GD2 using an IgG-scFv bispecific antibody with high affinity for GD2 and DOTA metal complex. Molecular cancer therapeutics.

[18] Cheal SM, Fung EK, Patel M, Xu H, Guo HF, Zanzonico PB (2017). Curative Multicycle Radioimmunotherapy Monitored by Quantitative SPECT/CT-Based Theranostics, Using Bispecific Antibody Pretargeting Strategy in Colorectal Cancer. Journal of nuclear medicine: official publication, Society of Nuclear Medicine.

[19] Cheal SM, Xu H, Guo HF, Lee SG, Punzalan B, Chalasani S (2016). Theranostic pretargeted radioimmunotherapy of colorectal cancer xenografts in mice using picomolar affinity (8)(6)Y- or (1)(7)(7)Lu-DOTA-Bn binding scFv C825/GPA33 IgG bispecific immunoconjugates. Eur J Nucl Med Mol Imaging.

[20] Cheal SM, Xu H, Guo HF, Patel M, Punzalan B, Fung EK (2018). Theranostic pretargeted radioimmunotherapy of internalizing solid tumor antigens in human tumor xenografts in mice: Curative treatment of HER2-positive breast carcinoma. Theranostics.

[21] Green DJ, Frayo SL, Lin Y, Hamlin DK, Fisher DR, Frost SH (2016). Comparative Analysis of Bispecific Antibody and Streptavidin-Targeted Radioimmunotherapy for B-cell Cancers. Cancer Res.

[22] Green DJ, O'Steen S, Lin Y, Comstock ML, Kenoyer AL, Hamlin DK (2018). CD38-bispecific antibody pretargeted radioimmunotherapy for multiple myeloma and other B-cell malignancies. Blood.

[23] McDevitt MR, Sgouros G, Sofou S (2018). Targeted and Nontargeted alpha-Particle Therapies. Annual review of biomedical engineering.

[24] Poty S, Francesconi LC, McDevitt MR, Morris MJ, Lewis JS (2018). alpha-Emitters for Radiotherapy: From Basic Radiochemistry to Clinical Studies-Part 1. J Nucl Med.

[25] Pandit-Taskar N (2019). Targeted Radioimmunotherapy and Theranostics with Alpha Emitters. J Med Imaging Radiat Sci.

[26] Tafreshi NK, Doligalski ML, Tichacek CJ, Pandya DN, Budzevich MM, El-Haddad G (2019). Development of Targeted Alpha Particle Therapy for Solid Tumors. Molecules.

[27] Aghevlian S, Boyle AJ, Reilly RM (2017). Radioimmunotherapy of cancer with high linear energy transfer (LET) radiation delivered by radionuclides emitting alpha-particles or Auger electrons. Adv Drug Deliv Rev.

[28] Jaggi JS, Seshan SV, McDevitt MR, LaPerle K, Sgouros G, Scheinberg DA (2005). Renal tubulointerstitial changes after internal irradiation with alpha-particle-emitting actinium daughters. Journal of the American Society of Nephrology: JASN.

[29] Nedrow JR, Josefsson A, Park S, Back T, Hobbs RF, Brayton C (2017). Pharmacokinetics, microscale distribution, and dosimetry of alpha-emitter-labeled anti-PD-L1 antibodies in an immune competent transgenic breast cancer model. EJNMMI research.

[30] Cheal SM, Patel M, Yang G, Veach DR, Xu H, Guo HF (2019). A N-acetylgalactosamino Dendron-Clearing Agent for High-Therapeutic Index DOTA-Hapten Pretargeted Radioimmunotherapy. Bioconjugate chemistry.

[31] McDevitt MR, Ma D, Simon J, Frank RK, Scheinberg DA (2002). Design and synthesis of 225Ac radioimmunopharmaceuticals. Appl Radiat Isot.

[32] Wei W, Rosenkrans ZT, Liu J, Huang G, Luo QY, Cai W (2020). ImmunoPET: Concept, Design, and Applications. Chem Rev.

[33] Poty S, Francesconi LC, McDevitt MR, Morris MJ, Lewis JS (2018). alpha-Emitters for Radiotherapy: From Basic Radiochemistry to Clinical Studies-Part 2. J Nucl Med.

[34] Miederer M, Scheinberg DA, McDevitt MR (2008). Realizing the potential of the Actinium-225 radionuclide generator in targeted alpha particle therapy applications. Adv Drug Deliv Rev.

[35] McDevitt MR, Scheinberg DA (2002). Ac-225 and her daughters: the many faces of Shiva. Cell Death Differ.

[36] Song H, Hobbs RF, Vajravelu R, Huso DL, Esaias C, Apostolidis C (2009). Radioimmunotherapy of breast cancer metastases with alpha-particle emitter 225Ac: comparing efficacy with 213Bi and 90Y. Cancer research.

[37] Schwartz J, Jaggi JS, O'Donoghue JA, Ruan S, McDevitt M, Larson SM (2011). Renal uptake of bismuth-213 and its contribution to kidney radiation dose following administration of actinium-225-labeled antibody. Physics in medicine and biology.

[38] Miederer M, McDevitt MR, Borchardt P, Bergman I, Kramer K, Cheung NK (2004). Treatment of neuroblastoma meningeal carcinomatosis with intrathecal application of alpha-emitting atomic nanogenerators targeting disialo-ganglioside GD2. Clin Cancer Res.

[39] Essler M, Gartner FC, Neff F, Blechert B, Senekowitsch-Schmidtke R, Bruchertseifer F (2012). Therapeutic efficacy and toxicity of 225Ac-labelled vs. 213Bi-labelled tumour-homing peptides in a preclinical mouse model of peritoneal carcinomatosis. Eur J Nucl Med Mol Imaging.

[40] Miederer M, Henriksen G, Alke A, Mossbrugger I, Quintanilla-Martinez L, Senekowitsch-Schmidtke R (2008). Preclinical evaluation of the alpha-particle generator nuclide 225Ac for somatostatin receptor radiotherapy of neuroendocrine tumors. Clin Cancer Res.

[41] Pandya DN, Hantgan R, Budzevich MM, Kock ND, Morse DL, Batista I (2016). Preliminary Therapy Evaluation of (225)Ac-DOTA-c(RGDyK) Demonstrates that Cerenkov Radiation Derived from (225)Ac Daughter Decay Can Be Detected by Optical Imaging for *In vivo* Tumor Visualization. Theranostics.

[42] Weiden PL, Breitz HB, Press O, Appelbaum JW, Bryan JK, Gaffigan S (2000). Pretargeted radioimmunotherapy (PRIT) for treatment of non-Hodgkin's lymphoma (NHL): initial phase I/II study results. Cancer Biother Radiopharm.

[43] Green DJ, Press OW (2017). Whither Radioimmunotherapy: To Be or Not To Be?. Cancer Res.

[44] Ackerman ME, Chalouni C, Schmidt MM, Raman VV, Ritter G, Old LJ (2008). A33 antigen displays persistent surface expression. Cancer Immunol Immunother.

[45] Thurber GM, Zajic SC, Wittrup KD (2007). Theoretic criteria for antibody penetration into solid tumors and micrometastases. J Nucl Med.

[46] Boudousq V, Bobyk L, Busson M, Garambois V, Jarlier M, Charalambatou P (2013). Comparison between internalizing anti-HER2 mAbs and non-internalizing anti-CEA mAbs in alpha-radioimmunotherapy of small volume peritoneal carcinomatosis using 212Pb. PLoS One.

[47] Nikula TK, McDevitt MR, Finn RD, Wu C, Kozak RW, Garmestani K (1999). Alpha-emitting bismuth cyclohexylbenzyl DTPA constructs of recombinant humanized anti-CD33 antibodies: pharmacokinetics, bioactivity, toxicity and chemistry. J Nucl Med.

[48] McDevitt MR, Ma D, Lai LT, Simon J, Borchardt P, Frank RK (2001). Tumor therapy with targeted atomic nanogenerators. Science.

[49] Cheung NK, Modak S, Lin Y, Guo H, Zanzonico P, Chung J (2004). Single-chain Fv-streptavidin substantially improved therapeutic index in multistep targeting directed at disialoganglioside GD2. J Nucl Med.

[50] Kulkarni HR, Singh A, Langbein T, Schuchardt C, Mueller D, Zhang J Theranostics of prostate cancer: from molecular imaging to precision molecular radiotherapy targeting the prostate specific membrane antigen. The British journal of radiology. 2018: 20180308.

[51] Mulvey JJ, Villa CH, McDevitt MR, Escorcia FE, Casey E, Scheinberg DA (2013). Self-assembly of carbon nanotubes and antibodies on tumours for targeted amplified delivery. Nature nanotechnology.

[52] Heskamp S, Hernandez R, Molkenboer-Kuenen JDM, Essler M, Bruchertseifer F, Morgenstern A (2017). alpha- Versus beta-Emitting Radionuclides for Pretargeted Radioimmunotherapy of Carcinoembryonic Antigen-Expressing Human Colon Cancer Xenografts. J Nucl Med.

[53] Poty S, Carter LM, Mandleywala K, Membreno R, Abdel-Atti D, Ragupathi A (2019). Leveraging Bioorthogonal Click Chemistry to Improve (225)Ac-Radioimmunotherapy of Pancreatic Ductal Adenocarcinoma. Clin Cancer Res.

