# Supplementary material for: Alpha radioimmunotherapy using 225Ac-proteus-DOTA for solid tumors - safety at curative doses
Source: Theranostics. 2020 Sep 14;10(25):11359–75. doi: 10.7150/thno.48810 (PMC7546012; doi:10.7150/thno.48810)
Supplement: Supplementary file 1 — Supplementary figures and tables. [file thnov10p11359s1.pdf]

Supplementary Materials for

Alpha radioimmunotherapy using ^{225}Ac-proteus-DOTA for solid tumors – safety at curative doses

Sarah M. Cheal[1], Michael R. McDevitt[2], Brian H. Santich[5,6], Mitesh Patel[1], Guangbin Yang[3], Edward K. Fung[4], Darren R. Veach[2], Meghan Bell[1], Afruja Ahad[1], Daniela Burnes Vargas[1], Blesida Punzalan[1], Naga Vara Kishore Pillarsetty[2], Hong Xu[5], Hong-fen Guo[5], Sébastien Monette[7], Adam O. Michel[7], Alessandra Piersigilli[7], David A. Scheinberg[1,8], Ouathek Ouerfelli[3], Nai-Kong V. Cheung[1,5], Steven M. Larson[1,2*]

[1]Molecular Pharmacology Program, Memorial Sloan Kettering Cancer Center, New York, NY
[2]Department of Radiology, Memorial Sloan Kettering Cancer Center, New York, NY
[3]Organic Synthesis Core Facility, Memorial Sloan Kettering Cancer Center, New York, NY
[4]Department of Medical Physics, Memorial Sloan Kettering Cancer Center, New York, NY
[5]Department of Pediatrics, Memorial Sloan Kettering Cancer Center, New York, NY
[6]Louis V. Gerstner Jr. Graduate School of Biomedical Sciences, Memorial Sloan Kettering Cancer Center, New York, NY
[7]Laboratory of Comparative Pathology, Memorial Sloan Kettering Cancer Center, Weill Cornell Medicine, and The Rockefeller University, New York, NY
[8]Immunology Program, Weill Cornell Medicine, New York, NY

*Corresponding Author:
Steven M. Larson, MD, FACNM, FACR; 415 East 68th Street, Z-2064, New York, NY 10065; Tel: 646-888-2212; Fax: 212-717-3263; E-mail: larsons@mskcc.org

General

p-SCN-Bn-DOTA was purchased from Macrocyclics and Amine-PEG$_4$–DOTA was purchased from CheMatech and used without further purification. Optima grade hydrochloric acid was purchased from Fisher Scientific. Chelex-100 resin, 200-400 mesh was purchased from Bio-Rad Laboratories. PD-10 gel-filtration size-exclusion (SE) columns (containing 8.3 mL of Sephadex™ G-25 resin in each column) were purchased from GE Healthcare Life Sciences. The HER2-targeting antibody trastuzumab was purchased commercially as Herceptin™ from Genentech/Roche. Lutetium(III) chloride hexahydrate (≥99.99% trace metal basis) and other starting materials and chemicals (synthesis-grade) were purchased from Sigma-Aldrich and used without further purification. All solvents used for HPLC analysis and compound purification were HPLC-grade and purchased from Fisher Scientific. All buffers and solutions were prepared using ultrapure water (18 MΩ/cm resistivity).

All LC-MS data was obtained with a Waters Autopure system comprising the following instrumentation: 2767 Sample Manager, 2545 Binary Gradient Module, System Fluidics Organizer, 2424 Evaporative Light Scattering Detector, 2998 Photodiode Array Detector, 3100 Mass Detector. HPLC solvents (solvent A, 0.05% trifluoroacetic acid (TFA) in water; solvent B, 0.05% TFA in acetonitrile (ACN)) were filtered before use. The analytical method was 5-25% solvent B in 10 min, 1.2 mL/min flow rate. Analytical columns: Waters XBridge, BEH300, C4, 3.5 µm, 4.6 x 50 mm and C18, 4 µm, 4.6 x 50 mm. Preparative method: 5-25% solvent B in 30 min, 20 mL/min flow rate. Preparative column: Waters XBridge Prep C18, 4 µm, Optimum Bed Density, 19 x 150 mm.

All NMR data were obtained with either a Bruker AV500 or AV 600 instruments at ambient temperature. The following abbreviations were used: singlet (s), broad singlet (bs), doublet (d), triplet (t), quartet (q), pentet (p), doublet of a doublet (dd), multiplet (m).

Synthesis of LuDOTA-Bn-PEG$_4$-DO3A (Proteus DOTA, Pr-DOTA)

Metal-loaded organic complexes such as DOTA (DOTA = 1,4,7,10-Tetraazacyclododecane-*N*,*N'*,*N''*,*N'''*-tetraacetic acid) complexes may present isomerism at times [1]. This is the case here for the DOTA complex with lutetium (p-SCN-Bn-DOTA·Lu^{3+} complex). The interconversion between possible complex diastereoisomers is shown below.

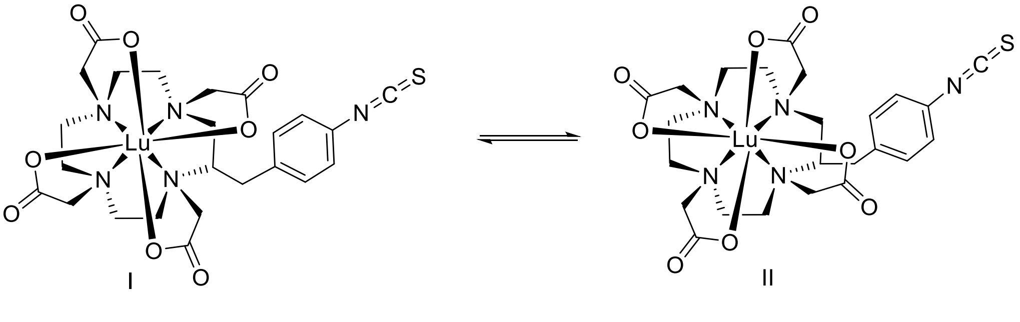

Indeed, we were able to isolate two complexes which we have not attempted to characterize except to note the differences in chromatographic as well as proton NMR data. The isomers are attributed to interconversion between square antiprismatic diastereoisomers of the complexes. No attempt was made at

observing enantiomers through chiral chromatography. Throughout the study, only the major isomer which may be attributed to *p*-SCN-Bn-DOTA·Lu^{3+} complex was used in the biological assessment.

DOTA-Bn-isothiocyanate (*p*-SCN-Bn-DOTA) was chosen for the synthesis as it has proven relatively stable during metal loading and subsequent purification and lyophilization. No attempts were made at optimization or recycling of possibly hydrolyzed isothiocyanate derivatives.

Experimental

Molecules with high metal complexing capacity such as DOTA, NOTA, etc., were conducted in glassware that was pre-washed with metal-free HCl, rinsed with high purity water or preferably glass-distilled water, then oven dried. Chromatography was carried out on manually packed glass columns to avoid loading the complexing agent with metal leached or extracted from metal column walls. The reverse phase (RP) purifications below were carried out on clean, metal-free glass columns which were packed manually with loose C-18 silica gel. No attempt was made to measure water content in the final complexes.

Chemistry

Loading of Lutetium into DOTA: *p*-SCN-Bn-DOTA·Lu^{3+} complex:

$LuCl_3$ ·6 H_2O (127 mg, 326 µmol) was added to 0.4 mL of a 0.4 M solution of sodium acetate, then *p*-isothiocyanatobenzyl-DOTA (*p*-SCN-Bn-DOTA, B-205, Macrocyclics, Inc. Plano, TX) (45 mg, 65 µmol) was introduced. The resulting mixture was stirred at room temperature (RT) overnight. Purification was performed by RP C-18 column, using 0-40% ACN in water as gradient. Appropriate fractions were pooled and lyophilized to provide 18 mg (38% yield) of desired complex as a white solid.

Bis-DOTA monocomplex of natural Lutetium

p-SCN-Bn-DOTA· Lu^{3+} complex (18 mg, 24.9 µmol) and NH_2-PEG-4-DOTA (17 mg, 24.4 µmol) were added to anhydrous dimethylformamide (DMF) (0.4 mL), followed by triethylamine (Et_3N) (20 µL, 140 *µ*mol). The mixture was stirred at RT for 3 h. Solvent was removed under high vacuum, and residue was

purified by RP C-18 column using 0-20% ACN in water as gradient to afford 2 isomers. At this point, the fast eluting isomer was obtained as triethyl ammonium salt. The second eluting fraction was re-purified on RP C-18 column using 0-8% ACN in water without base. Appropriate fractions were pooled and lyophilized. First eluting isomer (2.1 mg, 6.4%) and second isomer (11.2 mg, 34%) were isolated. First isomer: LC/MS m/z 1346.7 [calculated for $C_{50}H_{81}LuN_{11}O_{19}S$ (M+H) 1346.5]. ^{1}H NMR (600 MHz, D_2O, ppm), δ 7.25 (d, 2 H, J = 8.0 Hz), 7.19 (d, 2 H, J = 8.0 Hz), 3.75-3.21 (m, 55 H), 3.12 -2.42 (m, 21 H), 1.20 (t, 8 H, J = 7.3 Hz). Second isomer: LC/MS m/z 1346.7 [calculated for $C_{50}H_{81}LuN_{11}O_{19}S$ (M+H) 1346.5]. ^{1}H NMR (600 MHz, D_2O, ppm), δ 7.24-7.20 (m, 4 H), 3.75-3.00 (m, 57 H), 2.84-2.81 (m, 2 H), 2.77-2.74 (m, 1 H), 2.72-2.64 (m, 2 H), 2.61-2.51 (m, 3 H), 2.50-2.47 (m, 1 H), 2.44-2.38 (m, 2 H), 2.19 (m, 1 H). LC/MS: using 5-25% ACN (0.05% (TFA))/water (0.05% TFA).

Cell Culture

The GPA33-expressing human colorectal cancer cell line SW1222 was obtained from the Ludwig Institute for Cancer Immunotherapy (New York, NY). The HER2-expressing breast cancer cell line BT-474 and the GD2-expressing neuroblastoma cell line IMR-32 were obtained from American Type Culture Collection (Manassas, VA). The luciferase-labeled GD2-expressing neuroblastoma cell line IMR-32/luc was generated by stably expressing an SFG-GFLuc vector into the IMR-32 cells [1]. SW1222 cells were cultured in Minimal Essential Medium supplemented with 10% heat-inactivated fetal calf serum (FCS), 2.0 mM glutamine, 100 units/mL penicillin (P), and 100 µg/mL streptomycin (S). BT-474 cells were cultured in Dulbecco's modified Eagle-high-glucose/F-12 medium supplemented with non-essential amino acids (0.1 mM), 10% heat-inactivated FCS, 100 units/mL P, and 100 µg/mL S. IMR-32 cells were cultured in RPMI media supplemented with 10% heat-inactivated FCS, 100 units/mL P, and 100 µg/mL S. All cells were maintained in a 37°C environment containing 5% CO_2(g). Upon receipt of the cell line, cultures were established and cryopreserved in small aliquots to limit passages to less than three months and were periodically tested for mycoplasma negativity using a commercial kit (Lonza, Basel, Switzerland). A

solution of 0.25% trypsin/0.53 mM EDTA in Hanks Buffered Salt Solution without calcium and magnesium was used for trypsinization during cell passaging and harvesting.

Notes regarding animal care and models

After arrival at MSKCC, mice were acclimated in the vivarium for at least one week prior to experiments. For the BT-474 tumor model only, mice were either implanted with estrogen (17β-estradiol; 0.72 mg/pellet 60-day (d) release; Innovative Research of America) by trochar injection 3 d before inoculation with cells (for biodistribution studies) or received estrogen-supplemented water (for therapy studies; final β-estradiol (Sigma-Aldrich cat# E2758) concentration 8 µg/mL) from 1 week in advance of inoculation until sacrificed. Fresh-estradiol supplemented water was provided twice a week. The mice were housed in type II polycarbonate cages, fed with sterilized standard laboratory diet and received sterile water ad libitum. The animals were housed at approximately 22 °C, 60% relative humidity, and a 12 h light, 12 h dark cycle was maintained. For establishment of all tumors, groups of mice were inoculated with 5.0×10^6 cells (with the exception of SW1222 model, during which 3.0×10^6 cells or 5.0×10^6 cells were used for therapy and biodistribution studies, respectively) in a 200 µL cell suspension of a 1:1 mixture of media with reconstituted basement membrane (BD Matrigel, Collaborative Biomedical Products Inc., Bedford, MA) on lower flank via s.c. injection using a sterile syringe with a 28-gauge needle, and established tumors (100-300 mm^3) were observed within 7-10 d (SW1222) or 3-8 w (BT-474, IMR-32, or IMR-32/luc).

SPECT/CT image analysis

^{111}In SPECT image volume was acquired coaxially with a CT image volume. Images were processed using 3D Slicer v4.8.0. Segmented volumes of interest (VOIs) from a digital mouse atlas (Digimouse [2]) were individually manually positioned on the CT image and boundaries adjusted accordingly. VOIs were applied to the co-aligned SPECT image and voxel values extracted. Average activity concentration expressed as %IA/g for all voxels in each VOI (all data), as well as for the highest 10% of voxels in each VOI, were tabulated (pretargeting with BsAb and CA).

Macroscopic post-mortem examination and tissue sample collection

Immediately following euthanasia by CO_2 inhalation, blood collection by cardiocenthesis was performed, followed by complete necropsies (macroscopic examination, organ weight measurement, and tissue collection) under the supervision of a board-certified pathologist (SM, AOM, AP). Macroscopic lesions were recorded using standard macroscopic pathological descriptive terminology. Weights of the body and following organs were recorded: kidneys, liver, spleen, heart. All tissues were collected and fixed in 10% neutral buffered formalin (NBF) for histopathology.

Histopathology (microscopic examination)

After at least 72 h of fixation in 10% NFB, tissue listed below were trimmed and processed in xylene and ethanol, embedded in paraffin, sectioned at 5 µm thickness, and stained with hematoxylin and eosin. Bones were decalcified in a solution of formic acid and formaldehyde after fixation and prior to processing. All tissues were examined by a board-certified pathologist (SM, AOM, AP) and included: heart, lungs, thymus, kidneys, liver, gallbladder, stomach, duodenum, jejunum, ileum, cecum, colon, mesenteric lymph node, salivary glands, submandibular lymph node, uterus, cervix, vagina, urinary bladder, spleen, pancreas, adrenals, ovaries, oviducts, trachea, esophagus, thyroid, parathyroid, skin (trunk), mammary glands, bones (femur, tibia, sternum, vertebrae), bone marrow (femur, tibia, sternum, vertebrae), stifle joint, skeletal muscles (hind limb, spine), nerves (hind limb, spine), spinal cord, oral cavity, teeth, nasal cavity, eyes, harderian gland, bones (skull), pituitary, brain, and ears.

Hematology and serum chemistry

For hematology, blood was collected in tubes containing EDTA. Automated analysis was performed by the LCP on an IDEXX Procyte DX hematology analyzer and the following parameters were determined: white blood cell count (WBC), red blood cell count (RBC), hemoglobin concentration (HGB), hematocrit (HCT), mean corpuscular volume (MCV), mean corpuscular hemoglobin (MCH), mean corpuscular hemoglobin concentration (MCHC), red blood cell distribution width standard deviation and coefficient of variance

(RDW-SD and RDW-CV), platelet count (PLT), platelet distribution width (PDW), mean platelet volume (MPV), and relative and absolute counts of neutrophils (NEUT), lymphocytes (LYMPH), monocytes (MONO), eosinophils (EO), basophils (BASO), and reticulocytes (RET).

For serum chemistry, blood was collected in tubes containing a serum separator. The tubes were then centrifuged, and the serum was obtained for analysis. Serum chemistry was performed by the LCP on a Beckman Coulter AU680 analyzer for the following parameters: alkaline phosphatase concentration (ALP), alanine aminotransferase concentration (ALT), aspartate aminotransferase concentration (AST), creatine kinase concentration (CK), gamma-glutamyl transpeptidase concentration (GGT), albumin concentration (ALB), total protein concentration (TP), globulin concentration (GLOB), albumin/globulin ratio (A/G), total bilirubin concentration (TBIL), blood urea nitrogen concentration (BUN), creatinine concentration (CREA), cholesterol concentration (CHOL), triglycerides concentration (TRIG), glucose concentration (GLUC), calcium concentration (Ca), phosphorus concentration (P), chloride concentration (Cl), potassium concentration (K), sodium concentration (Na), Na/K ratio.

In vitro mixing of [^{225}Ac]Pr with an anti-HER2 BsAb, followed by in vivo targeting studies of HER2-expressing BT-474 tumors

[^{225}Ac]Pr was prepared to a final molar activity of 2.22 GBq/g [0.06 Ci/g] or 3108 GBq/mol [84 Ci/mol]. Approximately one week later, after storage of [^{225}Ac]Pr at RT, an *in vitro* mixing experiment consisting of mixing 145 µL of 6.91 mg/mL of anti-HER2-C825 (4.8 nmol of BsAb or 9.6 nmol of C825) and 90 µL of [^{225}Ac]Pr (18.056 kBq/8.64 nmol) for 1 h at RT (final volume: 235 µL) was performed. As a control, 1 mg of trastuzumab (6.67 nmol) was mixed with 90 µL of [^{225}Ac]Pr (17.316 kBq/8.64 nmol) in the same manner as the anti-HER2-C825 BsAb. These two solutions were run separately on PD-10 SE columns pre-equilibrated with saline + 1% human serum albumin and compared with the column elution of 90 µL (18.056 kBq/8.64 nmol) of [^{225}Ac]Pr only. Elution fractions were counted on the gamma-counter (Figure S2).

The two [^{225}Ac]Pr-BsAb complex fractions containing the most radioactivity (Fractions 5 and 6, corresponding to elutions 3.1-3.6 mL and 3.6-4.1 mL, respectively; 83% of total recovered activity) were combined (total volume: 1 mL). The following day, two groups of BT-474 tumor-bearing mice tumors (n = 3/group) were injected with either: [^{225}Ac]Pr only (0.51 nmol/mouse; ~1.11 kBq of ^{225}Ac/mouse) or [^{225}Ac]Pr-BsAb complex (1.79 nmol [^{225}Ac]Pr/mouse, 1.0 nmol of anti-HER2-C825/mouse; ~3.7 kBq ^{225}Ac/mouse) formulated in a total volume of 250 µL, and sacrificed 4 h p.i. for biodistribution assay. Note: a 4 h p.i. biodistribution time point was chosen to allow comparisons in uptake between the [^{225}Ac]Pr-BsAb complex and [^{225}Ac]Pr only, since the clearance of [^{225}Ac]Pr only is so rapid.

The data presented in Figure S3 shows that the [^{225}Ac]Pr-BsAb complex was able to target HER2(+) BT-474 tumor, while in comparison, [^{225}Ac]Pr showed negligible accumulation (12.4 ± 3.92 %IA/g or 0.50 ± 0.34 %IA/g, respectively) at 4 h p.i. Also for [^{225}Ac]Pr, all tissues assayed showed uptake of ≤ 2 %IA/g suggesting renal elimination and minimal retention in tissues. This was also evidenced by the remaining carcass ^{225}Ac activity, which for the [^{225}Ac]Pr-BsAb or [^{225}Ac]Pr groups was 48.1 ± 11.4 %IA or 2.85 ± 2.94 %IA. Notably, the blood activity of [^{225}Ac]Pr-BsAb (22.6 ± 4.18 %IA/g) was greater than tumor (12.4 ± 3.92 %IA/g) at 4 h p.i., suggesting that circulating [^{225}Ac]Pr-BsAb could have accumulated further in tumor if the animals were euthanized at a later time.

Additional details regarding in vivo biodistribution and clearance studies with Pr

The whole mouse activity assays shown in Figure S4A demonstrate rapid excretion of activity with ~90–94% removal by 6 h p.i. of [^{111}In]Pr. In the absence of specific tumor binding of the [^{111}In]Pr to administered BsAb, Pr traffics quickly and almost exclusively out of the body by renal clearance. This is further evidenced by image-based VOI analysis of tumor and individual organs as shown in Figures S4B and S4C. There is negligible uptake in tumor; kidney and bladder are the only organs assayed showing appreciable activity. Kidney activity is 36.5 ± 11.5 %IA/g at 1 h p.i. and falls rapidly to 1-2 %IA/g. The bulk of the activity is present in the bladder urine, which peaks at 300 %IA/g at 1 hr p.i. This is equivalent to 25.7% and 58.1% of total injected activity in each of the mice due to the small volume in the bladder.

Retention in the bladder, however, is primarily dependent on voiding, as evidenced by the rapid change in mouse #1 from 254 %IA/g to <2 %IA/g between 1 and 6 h p.i. of tracer.

Pathologist summary for unscheduled mortalities from study of toxicity of [^{225}Ac]Pr and additional details regarding [^{225}Ac]Pr toxicity

Regarding the two mice from the 18.5 kBq dose group, one on 36 d was submitted for necropsy because of 20% weight loss—no gross pathologic or histopathologic lesions were observed, and no significant findings were observed on hematology and serum chemistry—and one on 144 d, by which time necropsy was not possible. For the third unscheduled mortality, which was from the 296 kBq dose group: this animal was submitted for necropsy because of 20% weight loss on 123 d, and was found to have histiocytic and eosinophilic myocarditis, eosinophilic interstitial pneumonia, soft tissue hemorrhages, marked thrombocytopenia, and mild anemia with elevation of reticulocytes.

In the [^{225}Ac]Pr dose escalation study, no clinical toxicity (defined as >10% weight loss, presented in Figure 5A), changes in gross organ weights (Figure S6A), or radiation-induced histologic organ damage was observed at any [^{225}Ac]Pr dose level at necropsy performed at 145 d (Table S6). Immunohistochemical (IHC) analyses of the kidneys showed tubulointerstitial features that included minimal to mild multifocal cortical tubular degeneration and atrophy in 2/4 animals treated with the highest dose (296 kBq). Tubulointerstitial features including cytoplasmic vacuolization (as % of cells), tubulolysis with collapse (as % of tubules), atrophy (as % of tubules), and shrinkage/simplification (as % of residual tubules) were all <1% in the 296 kBq (highest) dose group (Table 1). Minimal to mild interstitial inflammatory infiltrates were rarely observed, interstitial fibrosis was not observed, and medullary tubules were normal (Table 1).

One histopathologic lesion observed in multiple mice was histiocytic and eosinophilic inflammation in some organs. These were inflammatory lesions composed predominantly of eosinophils and macrophages affecting multiple organs (although each affected mouse usually had lesions in only 1 or 2 of these organs): heart, lungs, kidneys, spleen, liver, and urinary bladder. Only the three highest-dose groups were affected, and there was an apparent dose-response (3/5 mice affected in 296 kBq group, 1/5 in

148 kBq, 1/5 in 74 kBq, 0/5 in 37 kBq, 0/4 in 18.5 kBq, 0/4 in 9.25 kBq, and 0/5 in saline vehicle). Similar lesions were observed during a toxicity study between days 100-200 in athymic nude mice treated with β-DOTA-PRIT (^{177}Lu: 165 MBq/mouse) [3, 4]. Based on blood counts, a mild (~10%) decrease of RBC mass was observed in the highest dose group (296 kBq). No significant effect that could be attributed to treatment with [^{225}Ac]Pr observed on serum chemistry at any dose level, including BUN levels and CREA (n = 34), which are biomarkers of renal function.

Supplemental Figures and Tables

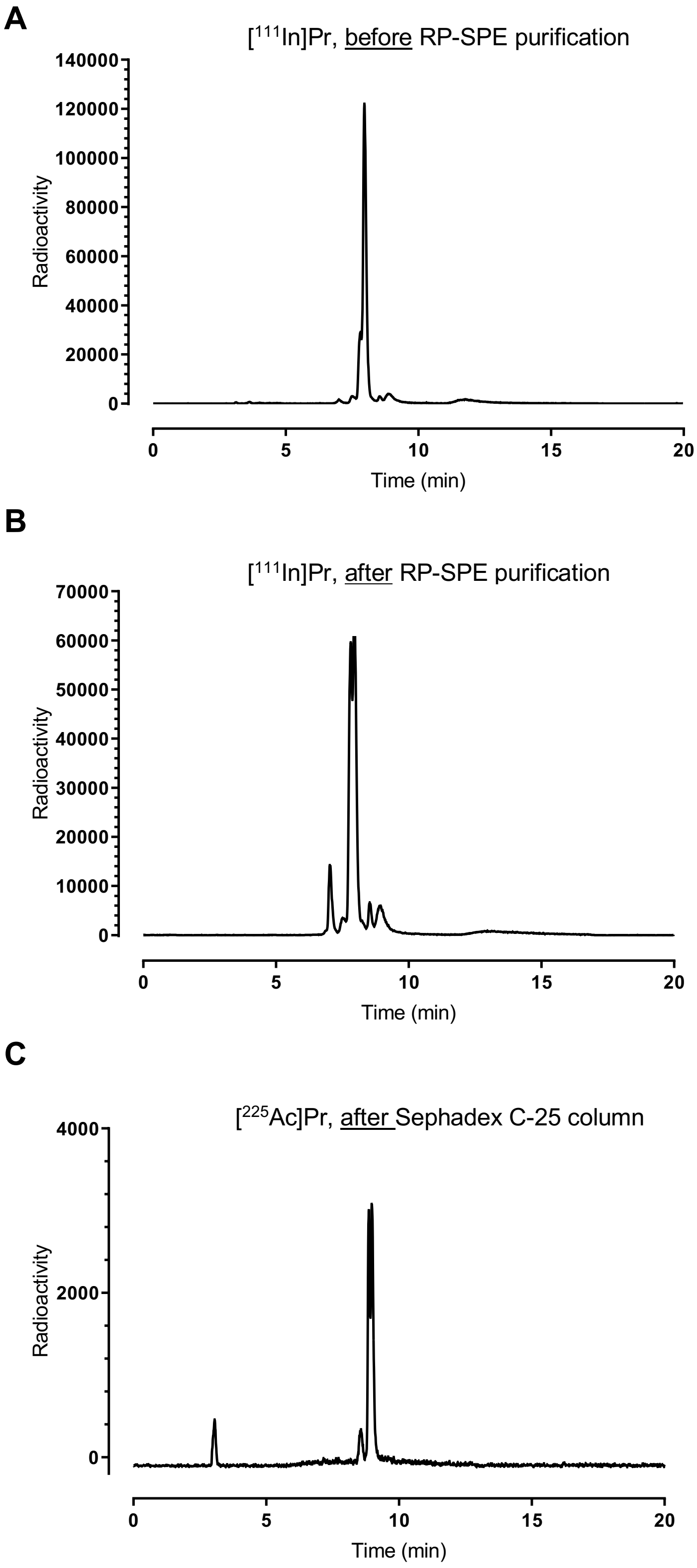

Figure S1. Radio-HPLC of $[^{111}In]Pr$ (**A** and **B**) and $[^{225}Ac]Pr$ (**C**).

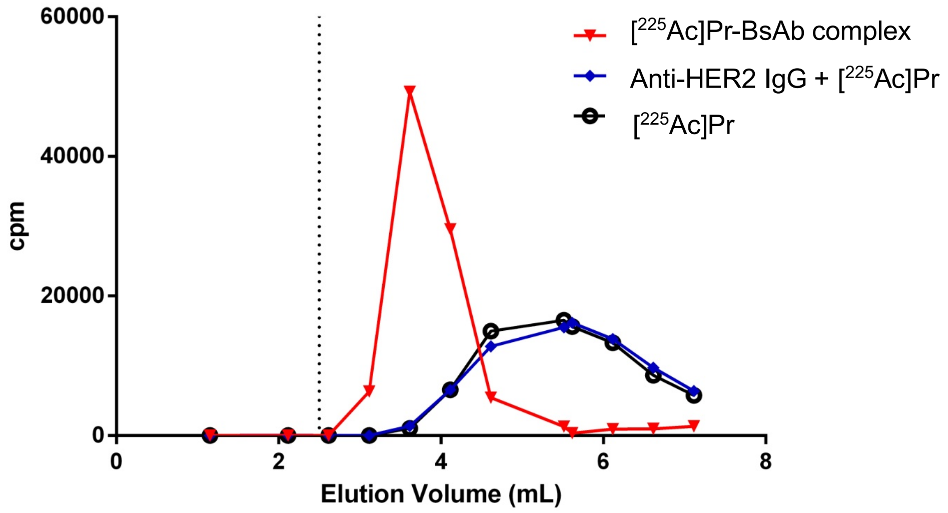

Figure S2. *In vitro* mixing of anti-HER2-C825 or control anti-HER2 IgG with [^{225}Ac]Pr, followed by PD-10 SE chromatography. Each point represenents an individual elution fraction. The dotted line indicates the void volume as specified by the column manufacturer. For reference, [^{225}Ac]Pr alone was also purified.

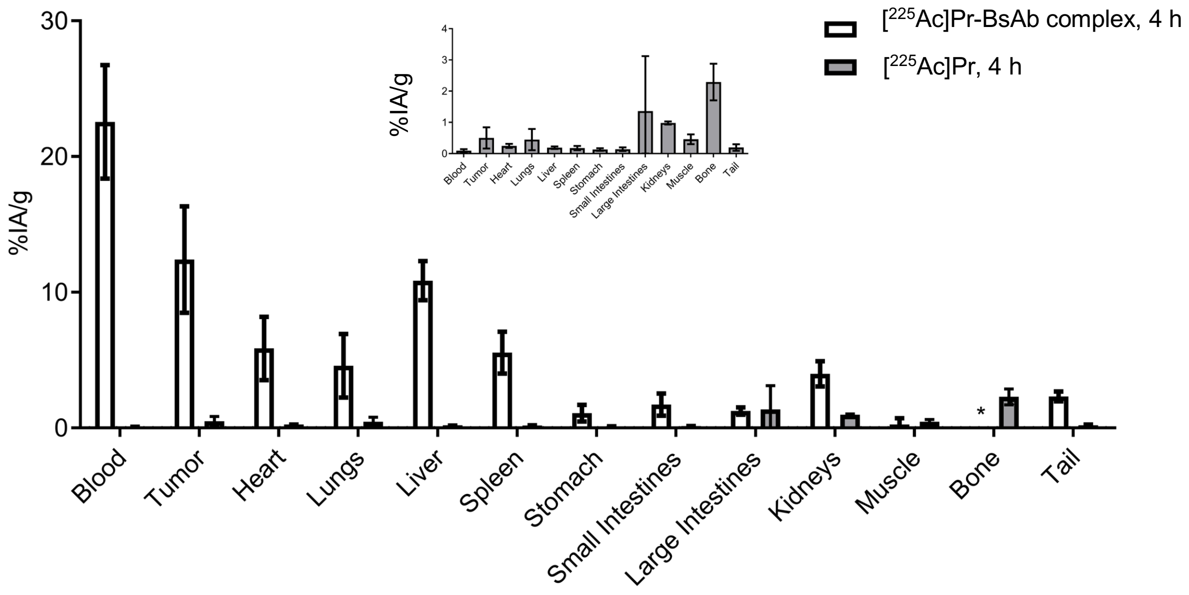

Figure S3. Biodistribution of the [^{225}Ac]Pr-BsAb complex (n = 3) and the [^{225}Ac]Pr tracer alone (n = 3) in BT-474 tumor-bearing mice at 4 h p.i. Data represent mean ± SD. Asterisk (*) indicates quantities below limit of detection.

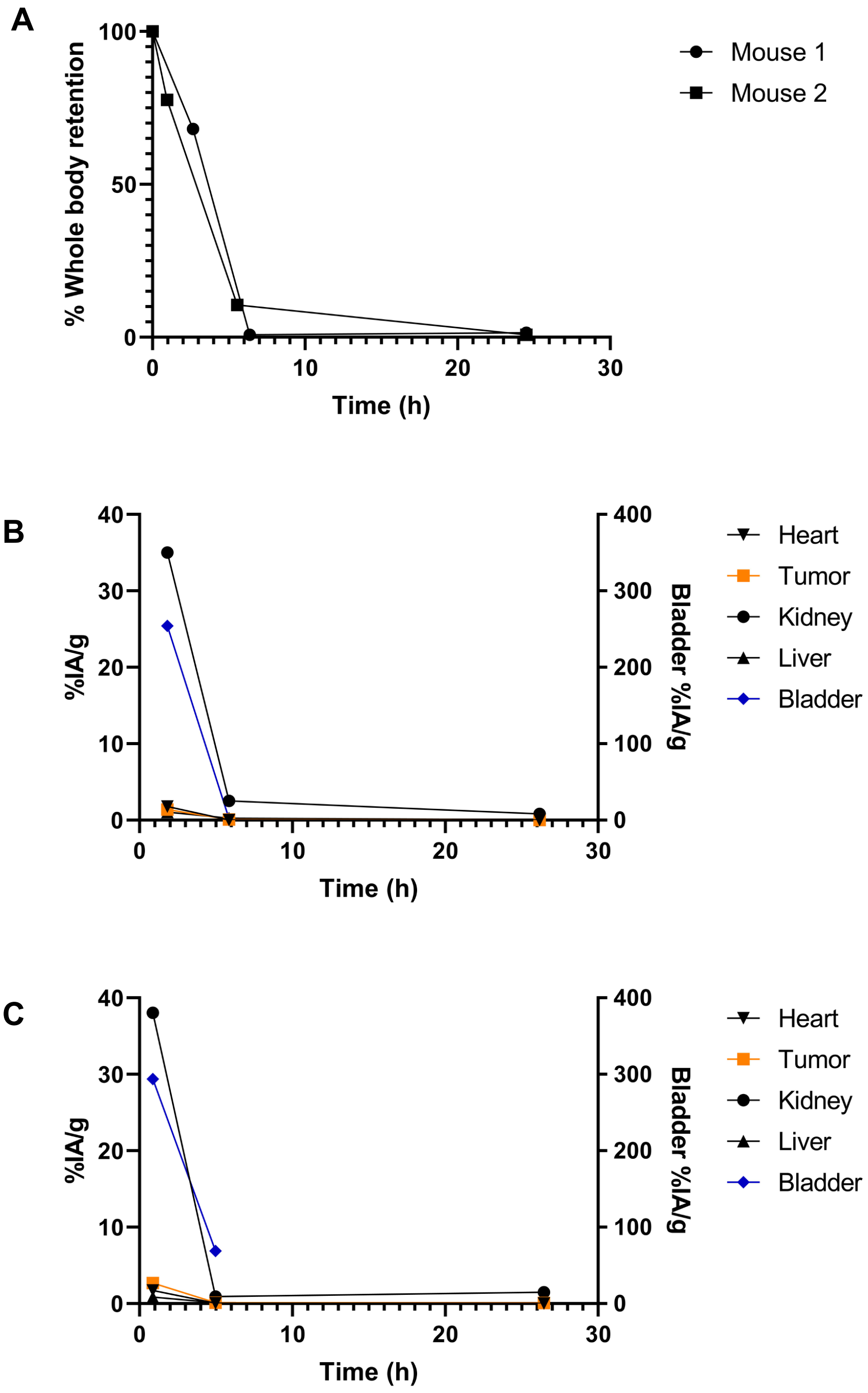

Figure S4. Whole-body clearance and SPECT/CT imaging of [^{111}In]Pr in BT-474 tumor-bearing mice. **A.** Whole-body clearance of [^{111}In]Pr revealed ~90–94% removal of activity by 6 h p.i. of tracer. Each point represents a single measurement. The whole-body clearance half-lives for mouse 1 (2.71 h; R^2 = 0.912) and mouse 2 (2.03 h; R^2 = 0.992) were determined by fitting the data to exponential model curves using MATLAB (Mathworks, Inc.). **B & C**. Mouse 1 and mouse 2 image-derived VOIs for tumor and select normal tissues (presented as mean), demonstrating rapid renal uptake and clearance.

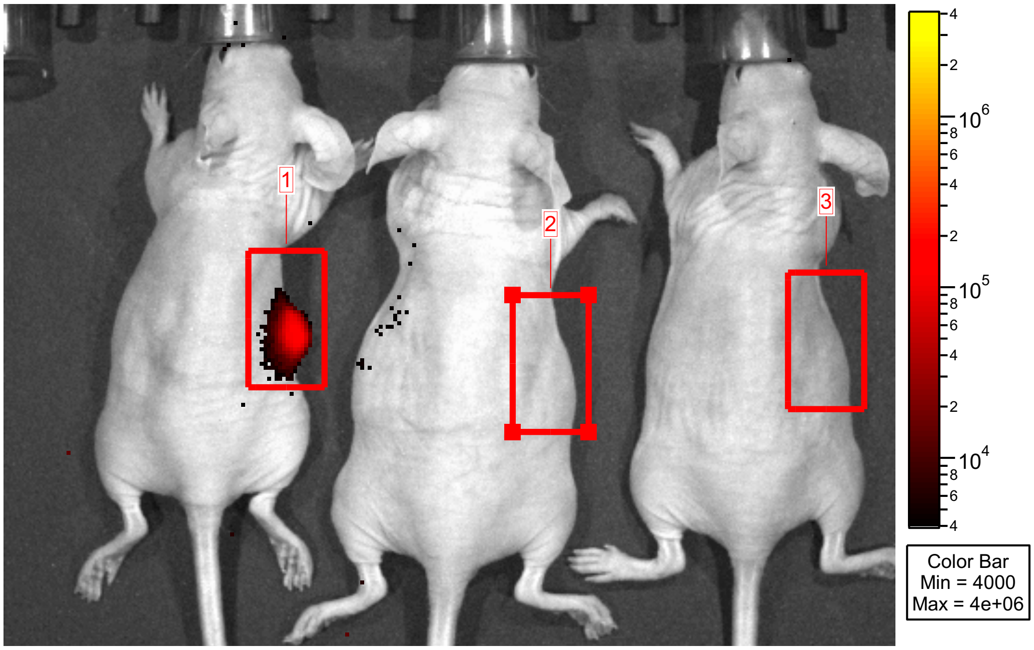

Figure S5. Representative BLI collected at ~110 d post-tumor inoculation or 68 d post-treatment from mice bearing IMR-32/luc xenografts that were treated with GD2 α-DOTA-PRIT (37 kBq). Mice were placed under anesthesia with isoflurane prior to retro-orbital injection of 100 µL D-Luciferin (30 mg/mL, dissolved in phosphate buffered saline). Images were obtained 5 min after injection for 30 s and 120 s exposures. Radiance (photons/s) was recorded (using Living Image® 4.5.2) from each individual tumor. The total flux determined by ROI image analysis (as photons/s): 3.32E+05, 2.66E+04, and 1.72E+04 for mouse 1, 2 and 3, respectively (positioned left to right).

Notably, in the weeks following BLI imaging, the tumor of mouse 1 spontaneously regressed to ~50-100 mm^3 and stabilized without recurrence up to the study endpoint of 210 d. BLI was collected for the remaining 4/7 treated animals at 130 d post-tumor inoculation or 82 d post-treatment. The total flux determined by ROI image analysis (as photons/s): 9.38E+04, 6.79E+04, 2.51E+05, and 7.76E+04 for mouse 4, 5, 6 and 7, respectively (not shown).

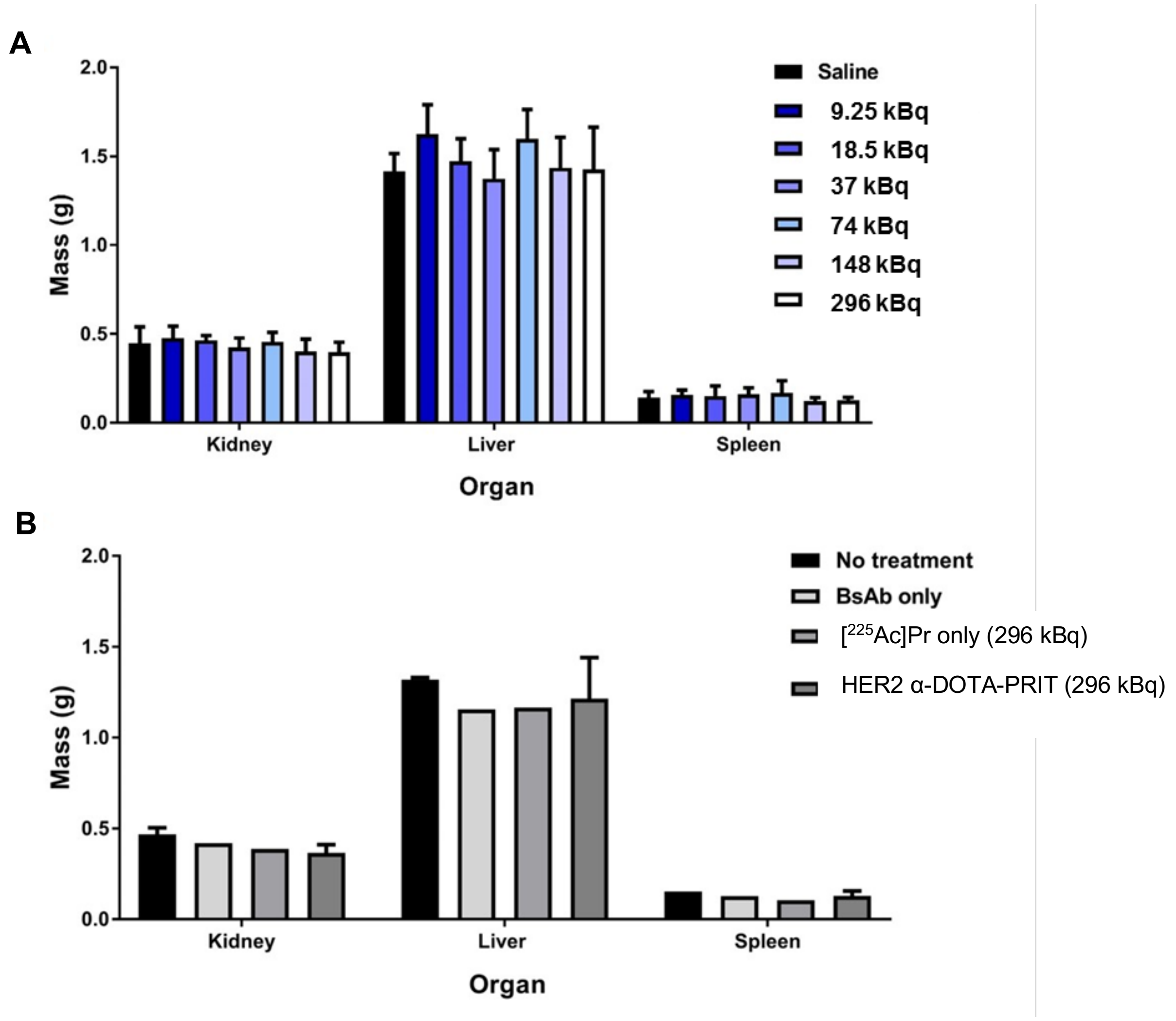

Figure S6. [^{225}Ac]Pr toxicity **A.** Select organ weights at 145 d taken at necropsy of tumor-free healthy female athymic nude mice treated with varying dose levels of [^{225}Ac]Pr only. No significant group differences were observed in organ weights. Data is presented as mean ± SD and *n* = 5 for all doses except for 18.5 kBq (*n* = 3) and 296 kBq (*n* = 4). **B.** Select organ weights at 150 d taken at necropsy of BT-474 tumor-bearing female athymic nude mice treated with HER2 α-DOTA-PRIT (296 kBq) or controls. No changes in gross organ weights with the exception of a moderate decrease in kidney weights were observed in organ weights. The kidney weights were 0.328-0.450 g and 0.388-0.493 g, for HER2 α-DOTA-PRIT (296 kBq) or controls, respectively. Data is presented as mean ± SD and *n* = 2 for no treatment, *n* = 1 for BsAb only, *n* = 1 for [^{225}Ac]Pr only (296 kBq), and *n* = 6 for HER2 α-DOTA-PRIT (296 kBq).

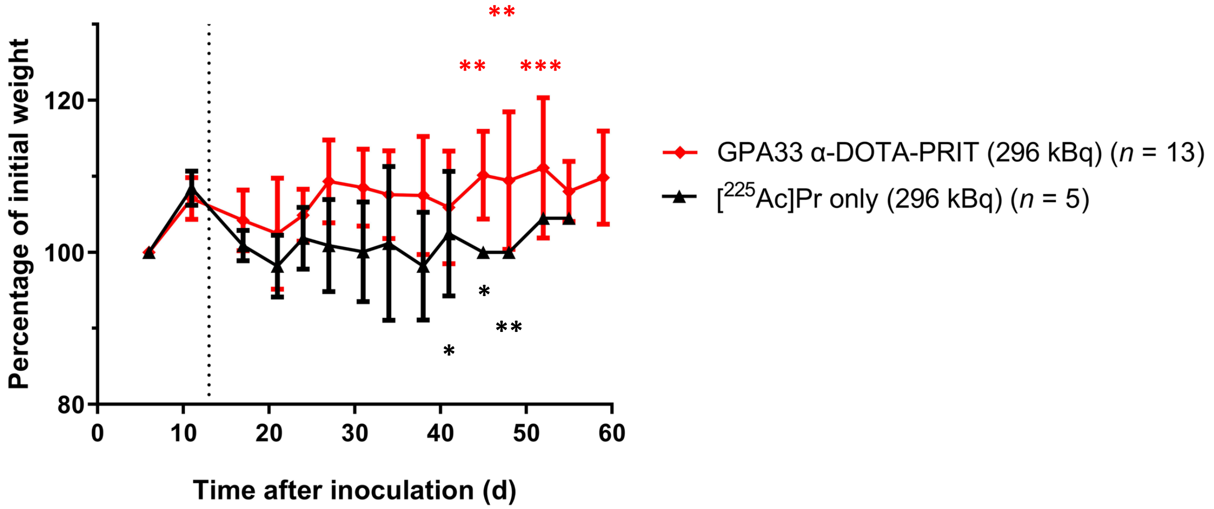

Figure S7. Mouse body weights during single-dose therapeutic studies with GPA33 α-DOTA-PRIT (296 kBq) or [^{225}Ac]Pr only (296 kBq). Data represent mean ± SD. Note: for GPA33 α-DOTA-PRIT (296 kBq) n = 11 at day 48, n = 9 at day 52, and n = 6 at day 55, and n = 6 at day 59. [^{225}Ac]Pr only n = 4 at day 41, n = 3 at day 45, and n = 1 at days 48, 52, and 55. Dotted line indicates time of treatment administration, which was at 13 days post-tumor inoculation. The study endpoint was at ~60 d post-inoculation/47 d post-treatment. Asterisk (*) indicates date of sacrifice. Overall, all treatments were well-tolerated by the mice, and no mice were removed from the study because of poor health.

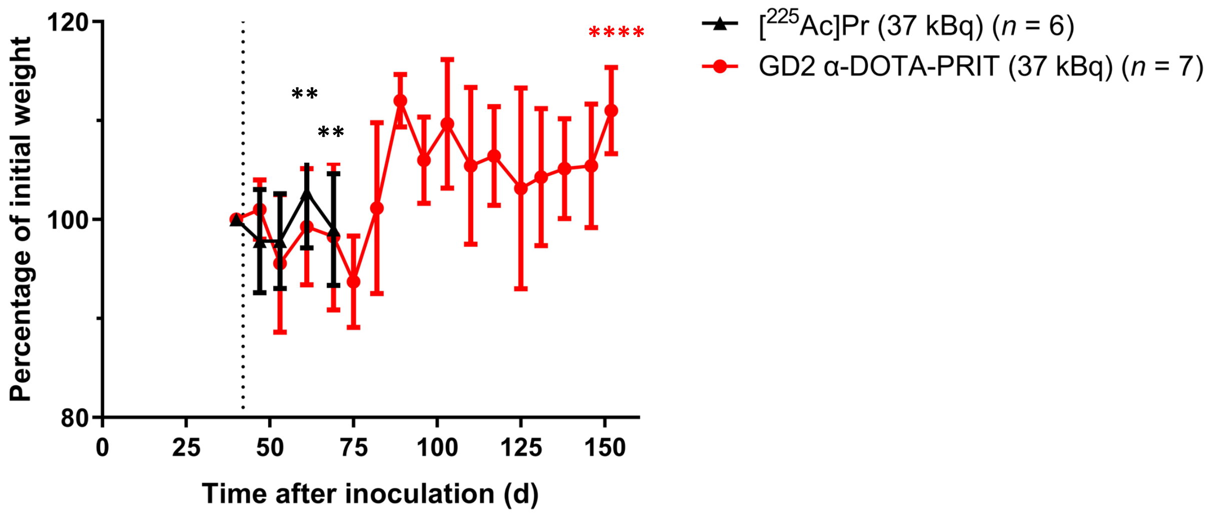

Figure S8. Mouse body weights during single-dose therapeutic studies with GD2 α-DOTA-PRIT (37 kBq) or [^{225}Ac]Pr only (37 kBq). Data represent mean ± SD. Note: for GD2 α-DOTA-PRIT (37 kBq) *n* = 3 at day 152. [^{225}Ac]Pr only *n* = 4 at day 61, and *n* = 2 at day 69. Black dotted line indicates time of treatment administration, which was at 42 days post-tumor inoculation. Overall, both treatments were well-tolerated by the mice, and no mice were removed from the study because of poor health.

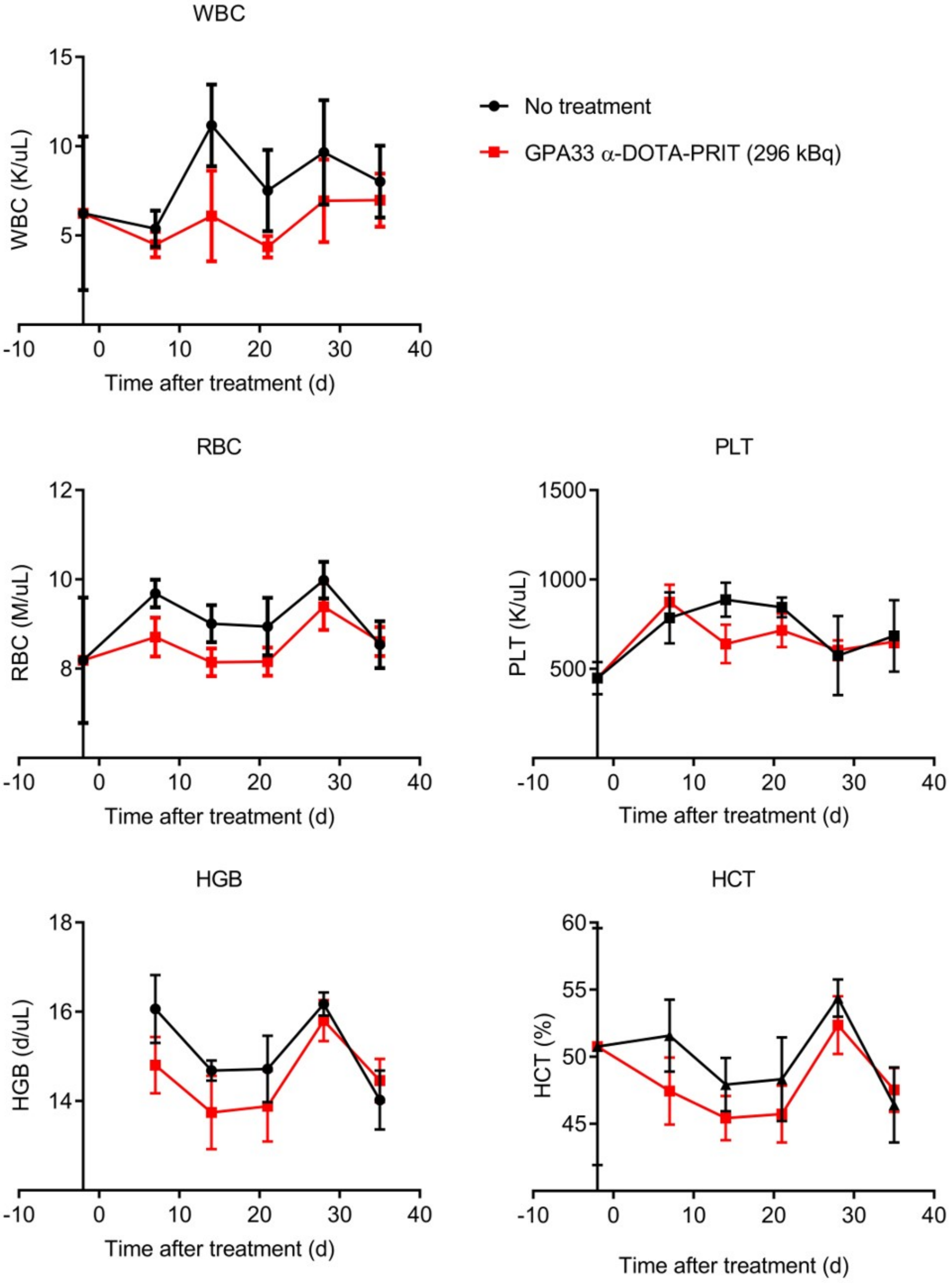

Figure S9. Select hematology data from groups of SW1222-tumored mice undergoing treatment with GPA33 α-DOTA-PRIT or no treatment control (*n* = 4-5/point). Data represent mean ± SD. WBC = white blood cells; RBC = red blood cells; PLT = platelets; HBG = hemoglobin; HCT = hematocrit. No significant differences were observed from pre-treatment values. Note: baseline data not available for HGB due to technical difficulties.

Table S1. Summary of additional [^{225}Ac]Pr preparations

Reaction	^{225}Ac activity MBq (mCi)	Pr µmoles (mg)	[^{225}Ac]Pr yield (%)	molar activity GBq/g (Ci/g)	molar activity GBq/mol (Ci/mol)
1	2.44 (0.066)	0.74(1.0)	94.0	2.22(0.06)	3108(84)
2	4.81 (0.130)	0.74(1.0)	94.7	4.44(0.12)	6142(166)
3	15.4 (0.415)	1.5(2.0)	100	7.4(0.20)	10138(274)
4	36.3 (1.01)	3.3(4.5)	97.0	7.8(0.21)	10989(297)
5	34.4(0.93)	1.9(2.5)	91.0	12.6(0.34)	16946(458)
6	9.25(0.25)	0.55(0.74)	95.0	11.5(0.31)	15281(413)

Table S2. Biodistribution of pretargeted [^{177}Lu]LuDOTA-Bn or [^{225}Ac]DOTA-Bn at 24 p.i. (n = 5 mice; 1.85 MBq and 3.7 MBq of ^{177}Lu and ^{225}Ac, respectively, 8-10 pmol) in IMR-32 tumor-bearing mice. Data (%IA/g) represent mean ± SD. Student's t test P values highlighted in red are considered significant (P < 0.05). The tumor masses were (presented as mean ± SD) 0.77 ± 0.62 g and 0.49 ± 0.28 g for ^{177}Lu- and ^{225}Ac-cohorts, respectively.

Organ	[^{177}Lu]LuDOTA-Bn (n = 5)	[^{225}Ac]DOTA-Bn (n = 5)	P value
Blood	0.49 ± 0.20	0.33 ± 0.08	0.0632
IMR-32 Tumor	10.30 ± 6.42	0.49 ± 0.28	0.0054
Heart	0.25 ± 0.07	0.24 ± 0.07	0.3983
Lungs	0.63 ± 0.18	0.23 ± 0.07	0.0009
Liver	0.66 ± 0.23	2.01 ± 0.98	0.0085
Spleen	2.03 ± 1.24	0.55 ± 0.20	0.0152
Stomach	0.14 ± 0.06	0.13 ± 0.04	0.4426
Small Intestine	0.20 ± 0.14	0.10 ± 0.05	0.0871
Large Intestine	0.24 ± 0.20	0.90 ± 0.62	0.0257
Kidneys	0.83 ± 0.14	1.16 ± 0.21	0.0107
Muscle	0.20 ± 0.13	0.05 ± 0.01	0.0158
Bone	0.17 ± 0.10	0.60 ± 0.16	0.0005

Table S3. Biodistribution of [^{111}In]Pr or [^{225}Ac]Pr in healthy tumor-free athymic nu/nu female mice at times indicated p.i. of tracer. Data (%IA/g) represent mean ± SD. ^{b}below the limit of detection.

Organ	[^{111}In]Pr alone (n = 5) 3.38 nmol/740 kBq 4 h p.i.	[^{225}Ac]Pr alone (n = 3) 198 pmol/1.85 kBq 1 h p.i.	[^{111}In]Pr alone (n = 3) 200 pmol/740 kBq 1 h p.i.
Blood	0.03 ± 0.00	0.31 ± 0.54	0.18 ± 0.10
Heart	0.03 ± 0.02	^{b}	0.08 ± 0.04
Lungs	0.07 ± 0.05	^{b}	0.15 ± 0.01
Liver	0.09 ± 0.02	0.01 ± 0.02	0.08 ± 0.00
Spleen	0.05 ± 0.01	0.04 ± 0.06	0.06 ± 0.01
Stomach	0.06 ± 0.03	0.24 ± 0.38	0.20 ± 0.25
Small Intestine	0.19 ± 0.14	0.16 ± 0.26	0.15 ± 0.19
Large Intestine	0.37 ± 0.21	0.02 ± 0.02	0.06 ± 0.02
Kidneys	1.07 ± 0.25	0.63 ± 0.41	1.01 ± 0.13
Muscle	0.04 ± 0.04	0.98 ± 0.91	0.08 ± 0.11
Bone	0.02 ± 0.02	0.45 ± 0.79	0.10 ± 0.09

Table S4. Biodistribution of GPA33 pretargeted [^{225}Ac]Pr or [^{111}In]Pr at 24 p.i. in SW1222 tumor-bearing mice. Data (%IA/g) represent mean ± SD. [a]only $n = 2$ due to technical reasons. [b]below the limit of detection. Note: the single mouse administered pretargeted [^{111}In]Pr (790 pmol/7.67 MBq) was imaged by SPECT/CT at 20 h p.i. prior to biodistribution at 24 h p.i. (Figure 3C).

Organ	Pretargeted [^{225}Ac]Pr (n = 3) 182 pmol/1.85 kBq 24 h p.i.	Pretargeted [^{111}In]Pr (n = 4) 172 pmol/1.67 MBq 24 h p.i.	Pretargeted [^{111}In]Pr (n = 1) 790 pmol/7.67 MBq 24 h p.i.
Blood	0.94 ± 0.37[a]	0.76 ± 0.38	0.16
SW1222 tumor	16.71 ± 5.11	13.19 ± 3.88	6.70
Heart	0.28 ± 0.48	0.28 ± 0.08	0.09
Lungs	0.70 ± 1.16	0.40 ± 0.14	0.17
Liver	1.40 ± 1.42	0.47 ± 0.20	0.22
Spleen	0.54 ± 1.61	0.29 ± 0.12	0.10
Stomach	0.07 ± 0.13	0.09 ± 0.06	0.03
Small Intestine	0.16 ± 0.31	0.12 ± 0.04	0.06
Large Intestine	0.11 ± 0.21	0.19 ± 0.16	0.06
Kidneys	1.08 ± 0.95	1.02 ± 0.38	0.65
Muscle	0.13 ± 0.40	0.12 ± 0.04	0.04
Bone	[b]	0.16 ± 0.06	0.05
Tumor-to-tissue ratios			
Blood	17.9 ± 4.0	17.3 ± 4.4	41.1
Heart	60.4 ± 35.8	47.5 ± 12.1	75.3
Lungs	24.0 ± 13.5	33.4 ± 8.5	40.5
Liver	11.9 ± 4.2	28.4 ± 7.2	31.2
Spleen	31.1 ± 31.3	45.1 ± 11.4	65.8
Stomach	238.8 ± 150.7	155.1 ± 39.8	229
Small Intestine	102.3 ± 67.1	107.6 ± 27.5	110
Large Intestine	147.5 ± 90.9	70.3 ± 18.9	119
Kidneys	15.5 ± 4.8	12.9 ± 3.3	10
Muscle	128.6 ± 129.2	112.2 ± 28.5	162
Bone	---	85.1 ± 21.7	127

Table S5. Statistical comparison of GPA33 pretargeted [^{225}Ac]Pr and [^{111}In]Pr at 24 p.i. in SW1222 tumor-bearing mice using Student's *t* test. Student's *t* test *P* values highlighted in red are considered significant ($P < 0.05$). Biodistribution data (%IA/g) represent mean ± SD.

Organ	Pretargeted [^{225}Ac]Pr ($n = 3$) 182 pmol/1.85 kBq	Pretargeted [^{111}In]Pr ($n = 4$) 172 pmol/1.67 MBq	*P* value
Blood	0.94 ± 0.37[a]	0.76 ± 0.38	0.385
SW1222 Tumor	16.71 ± 5.11	13.19 ± 3.88	0.475
Heart	0.28 ± 0.48	0.28 ± 0.08	0.242
Lungs	0.70 ± 1.16	0.40 ± 0.14	0.202
Liver	1.40 ± 1.42	0.47 ± 0.20	0.066
Spleen	0.54 ± 1.61	0.29 ± 0.12	0.256
Stomach	0.07 ± 0.13	0.09 ± 0.06	0.448
Small Intestine	0.16 ± 0.31	0.12 ± 0.04	0.305
Large Intestine	0.11 ± 0.21	0.19 ± 0.16	0.264
Kidneys	1.08 ± 0.95	1.02 ± 0.38	0.374
Muscle	0.13 ± 0.40	0.12 ± 0.04	0.378
Bone	[b]	0.16 ± 0.06	---

Table S6. Single-dose toxicity testing in tumor-free female athymic nude mice 145 d after systemic administration of saline vehicle (Control group, *n*=5) versus varying dose levels of [^{225}Ac]Pr (Treatment groups, *n*=5/group; 9.25, 18.5, 37, 74, 148, and 296 kBq). Unscheduled mortalities included: two mice of 18.5 kBq; single mouse of 296 kBq. Red text: Significant lesions (probably treatment-related). Note: The following histopathologic findings were not reported in the table as they are normal phenotype for this mouse strains and were observed in all animals: Thymus: Lymphoid depletion; Cystic epithelial structures.; Mesenteric and submandibular lymph nodes: Paracortical lymphoid depletion.; Spleen: White pulp, lymphoid depletion of periarteriolar sheaths.; Skin: Follicular dysplasia.

Animal ID	Anatomic Pathology	
	Gross Finding(s)	Microscopic Finding(s)
#1 Saline	• Body weight is 25.173 g.<br>• One (1) 2 mm diameter white focus in the mesentery, adjacent to the mesenteric lymph node.	**All tissues are normal unless otherwise described.**<br>• Kidneys: Lymphocytic interstitial infiltrate, multifocal, bilateral, mild.<br>• Liver: Lymphocytic infiltrate, portal and centrilobular perivascular, multifocal, mild.<br>• Stomach: Mucosal infiltrate, lymphocytic and eosinophilic, multifocal, mild.<br>• Mesenteric lymph node: Paracortical lymphoid depletion.<br>• Skin (trunk): Acanthosis and hyperkeratosis, moderate, diffuse, with chronic dermatitis, mild, multifocal.<br>• Eyes: Meibomian adenitis, pyogranulomatousa, focal, unilateral, minimal.<br>• Cervix, parathyroid: Not present on slide.<br>• Other: Abnormal mesenteric tissue observed grossly: Lymph node, with follicular lymphoid hyperplasia.
#2 Saline	• Body weight is 24.325 g.<br>• No gross lesions are observed.	**All tissues are normal unless otherwise described.**<br>• Lungs: Peribronchiolar and perivascular lymphocytic infiltrate, mild, multifocal.<br>• Kidneys: Tubular basophilia, multifocal, bilateral, minimal; Glomerular hyalinosis, multifocal, minimal, bilateral.<br>• Liver: Lymphocytic infiltrate, portal and centrilobular perivascular, multifocal, mild.<br>• Gallbladder: Mucosal lymphocytic infiltrate, minimal, focal.<br>• Stomach: Mucosal infiltrate, lymphocytic, multifocal, mild.<br>• Mesenteric lymph node: Follicular lymphoid hyperplasia; Plasmacytosis.<br>• Submandibular lymph node: Follicular lymphoid hyperplasia; Plasmacytosis.<br>• Trachea: Mucosal lymphocytic infiltrate, mild, multifocal.<br>• Skin (trunk): Acanthosis and hyperkeratosis, moderate, diffuse, with chronic dermatitis, mild, multifocal.<br>• Pituitary: Not present on slide.<br>• Other: Not applicable.

Table S6, cont'd. Single-dose toxicity testing in tumor-free female athymic nude mice 145 d after systemic administration of saline vehicle versus varying dose levels of [^{225}Ac]Pr.

#3 Saline	• Body weight is 25.900 g.<br>• Right inguinal lymph node is mildly enlarged. Liver and kidneys appear slightly pale.	**All tissues are normal unless otherwise described.**<br>• Lungs: Peribronchiolar and perivascular lymphoplasmacytic infiltrate, mild, multifocal.<br>• Kidneys: Lymphoplasmacytic interstitial infiltrate, multifocal, unilateral, mild; Tubular basophilia, focal, unilateral, minimal; Tubular ectasia, focal, unilateral, minimal; Glomerular hyalinosis, multifocal, mild, bilateral.<br>• Liver: Lymphoplasmacytic infiltrate, portal and centrilobular perivascular, multifocal, moderate.<br>• Duodenum, jejunum, ileum: Mucosal infiltrate, eosinophilic, segmental, multifocal, minimal.<br>• Mesenteric lymph node: Follicular lymphoid hyperplasia.<br>• Submandibular lymph node: Follicular lymphoid hyperplasia.<br>• Urinary bladder: Lymphoplasmacytic mucosal infiltrate, multifocal, mild.<br>• Spleen: Follicular lymphoid hyperplasia; Plasmacytosis.<br>• Skin (trunk): Acanthosis and hyperkeratosis, moderate, diffuse, with chronic dermatitis, mild, multifocal.<br>• Pituitary: Not present on slide.<br>• Other: Not applicable.
#4 Saline	• Body weight is 28.594 g.<br>• No gross lesions are observed.	**All tissues are normal unless otherwise described.**<br>• Lungs: Granulomatous pneumonia, mild, multifocal.<br>• Liver: Lymphocytic infiltrate, portal and centrilobular perivascular, multifocal, mild.<br>• Duodenum, jejunum, ileum: Mucosal infiltrate, eosinophilic, segmental, multifocal, minimal.<br>• Mesenteric lymph node: Follicular lymphoid hyperplasia; Plasmacytosis.<br>• Submandibular lymph node: Follicular lymphoid hyperplasia; Plasmacytosis.<br>• Urinary bladder: Lymphocytic mucosal infiltrate, multifocal, minimal.<br>• Spleen: Follicular lymphoid hyperplasia.<br>• Skin (trunk): Acanthosis and hyperkeratosis, moderate, diffuse, with chronic dermatitis, mild, multifocal.<br>• Gallbladder, cervix, parathyroid, pituitary: Not present on slide.<br>• Other: Not applicable.
#5 Saline	• Body weight is 28.116 g.<br>• Right kidney appears slightly enlarged and has multiple pale foci.	**All tissues are normal unless otherwise described.**<br>• Kidneys: Tubular degeneration and atrophy, cortical, focal, unilateral, minimal.<br>• Liver: Lymphocytic infiltrate, portal and centrilobular perivascular, multifocal, mild.<br>• Mesenteric lymph node: Follicular lymphoid hyperplasia.<br>• Urinary bladder: Cystitis, eosinophilic and lymphocytic, moderate, diffuse.<br>• Skin (trunk): Acanthosis and hyperkeratosis, moderate, diffuse, with chronic dermatitis, mild, multifocal.<br>• Submandibular lymph node, pituitary: Not present on slide.<br>• Other: Not applicable.

Table S6, cont'd. Single-dose toxicity testing in tumor-free female athymic nude mice 145 d after systemic administration of saline vehicle versus varying dose levels of [^{225}Ac]Pr.

#1 9.25 kBq	• Body weight is 30.223 g.<br>• Spleen has irregular edges and a pink focus, 1 mm in diameter.	**All tissues are normal unless otherwise described.**<br>• Lungs: Peribronchiolar and perivascular lymphocytic infiltrate, mild, multifocal.<br>• Kidneys: Tubular basophilia, focal, unilateral, minimal.<br>• Liver: Lymphocytic infiltrate, portal and centrilobular perivascular, multifocal, mild.<br>• Stomach: Mucosal infiltrate, lymphocytic and eosinophilic, multifocal, mild.<br>• Mesenteric lymph node: Follicular lymphoid hyperplasia.<br>• Submandibular lymph node: Follicular lymphoid hyperplasia.<br>• Urinary bladder: Lymphocytic mucosal infiltrate, multifocal, minimal.<br>• Pancreas: Periductal infiltrate, eosinophilic, minimal, focal.<br>• Skin (trunk): Acanthosis and hyperkeratosis, moderate, diffuse, with chronic dermatitis, mild, multifocal.<br>• Uterus, cervix, vagina, parathyroid, pituitary, ears: Not present on slide.<br>• Other: Not applicable.
#2 9.25 kBq	• Body weight is 27.177 g.<br>• Cecum and colon are mildly dilated; Mandibular lymph nodes are slightly enlarged.	**All tissues are normal unless otherwise described.**<br>• Liver: Lymphocytic infiltrate, portal and centrilobular perivascular, multifocal, mild; Random infiltrate, lymphocytic, histiocytic, neutrophilic, with hepatocyte necrosis, multifocal, minimal.<br>• Mesenteric lymph node: Plasmacytosis; Follicular lymphoid hyperplasia.<br>• Skin (trunk): Acanthosis and hyperkeratosis, moderate, diffuse, with chronic dermatitis, mild, multifocal.<br>• Parathyroid, mammary glands, pituitary: Not present on slide.<br>• Other: Not applicable.
#3 9.25 kBq	• Body weight is 25.484 g.<br>• No gross lesions are observed.	**All tissues are normal unless otherwise described.**<br>• Lungs: Neutrophilic and histiocytic pneumonia, mild, focal.<br>• Liver: Kupffer cell cytoplasmic pigment, most consistent with hemosiderin, moderate, diffuse; Extramedullary hematopoiesis, focal, minimal.<br>• Mesenteric lymph node: Plasmacytosis; Follicular lymphoid hyperplasia.<br>• Urinary bladder: Lymphoplasmacytic mucosal infiltrate, multifocal, minimal.<br>• Spleen: Follicular lymphoid hyperplasia.<br>• Skin (trunk): Acanthosis and hyperkeratosis, moderate, diffuse, with chronic dermatitis, mild, multifocal.<br>• Colon, submandibular lymph node, parathyroid, mammary glands: Not present on slide.<br>• Other: Not applicable.

Table S6, cont'd. Single-dose toxicity testing in tumor-free female athymic nude mice 145 d after systemic administration of saline vehicle versus varying dose levels of [^{225}Ac]Pr.

#4 9.25 kBq	• Body weight is 30.691 g.<br>• Small intestine has prominent Peyer's patches. Heart surface has white foci.	**All tissues are normal unless otherwise described.**<br>• Heart: Epicardial mineralization and fibrosis, right ventricular, moderate, multifocal.<br>• Kidneys: Tubular degeneration, multifocal, bilateral, minimal.<br>• Liver: Lymphocytic infiltrate, portal and centrilobular perivascular, multifocal, minimal.<br>• Mesenteric lymph node: Follicular lymphoid hyperplasia.<br>• Skin (trunk): Acanthosis and hyperkeratosis, moderate, diffuse, with chronic dermatitis, mild, multifocal.<br>• Vagina, parathyroid: Not present on slide.<br>• Other: Not applicable.
#5 9.25 kBq	• Body weight is 29.648 g.<br>• No gross lesions are observed.	**All tissues are normal unless otherwise described.**<br>• Lungs: Peribronchiolar and perivascular lymphocytic infiltrate, mild, multifocal.<br>• Kidneys: Glomerular hyalinosis, multifocal, minimal, bilateral.<br>• Liver: Lymphocytic infiltrate, portal and centrilobular perivascular, multifocal, moderate.<br>• Stomach: Submucosal infiltrate, lymphocytic, multifocal, mild.<br>• Mesenteric lymph node: Plasmacytosis; Follicular lymphoid hyperplasia.<br>• Urinary bladder: Lymphocytic mucosal infiltrate, focal, minimal.<br>• Spleen: Follicular lymphoid hyperplasia.<br>• Skin (trunk): Acanthosis and hyperkeratosis, moderate, diffuse, with chronic dermatitis, mild, multifocal.<br>• Thymus, ears: Not present on slide.<br>• Other: Not applicable.

Table S6, cont'd. Single-dose toxicity testing in tumor-free female athymic nude mice 145 d after systemic administration of saline vehicle versus varying dose levels of [^{225}Ac]Pr.

#1 18.5 kBq	• Body weight is 27.362 g.<br>• No gross lesions are observed.	**All tissues are normal unless otherwise described.**<br>• Lungs: Peribronchiolar and perivascular lymphocytic infiltrate, minimal, multifocal.<br>• Kidneys: Lymphocytic interstitial infiltrate, multifocal, bilateral, mild.<br>• Liver: Lymphocytic infiltrate, portal and centrilobular perivascular, multifocal, marked.<br>• Stomach: Normal (only nonglandular portion is present in section)<br>• Urinary bladder: Lymphocytic mucosal infiltrate, multifocal, moderate.<br>• Spleen: Splenitis, granulomatous, with intrahistiocytic pigment, white pulp, diffuse, mild.<br>• Skin (trunk): Acanthosis and hyperkeratosis, moderate, diffuse, with chronic dermatitis, mild, multifocal.<br>• Submandibular lymph node, ovaries, parathyroid, mammary glands, ears: Not present on slide.<br>• Other: Not applicable.
#2 18.5 kBq	• Body weight is 27.415 g.<br>• No gross lesions are observed.	**All tissues are normal unless otherwise described.**<br>• Lungs: Peribronchiolar and perivascular lymphocytic infiltrate, minimal, multifocal.<br>• Kidneys: Tubular basophilia, multifocal, bilateral, minimal; Glomerular hyalinosis, multifocal, mild, bilateral.<br>• Liver: Lymphocytic infiltrate, portal and centrilobular perivascular, multifocal, mild.<br>• Stomach: Mucosal infiltrate, lymphocytic and eosinophilic, focal, minimal.<br>• Skin (trunk): Acanthosis and hyperkeratosis, moderate, diffuse, with chronic dermatitis, mild, multifocal.<br>• Gallbladder, cervix, parathyroid, mammary glands: Not present on slide.<br>• Other: Not applicable.
#3 18.5 kBq	• Body weight is 27.863 g.<br>• Two (2) 2 mm diameter white foci in the mesentery, adjacent to the mesenteric lymph node.	**All tissues are normal unless otherwise described.**<br>• Kidneys: Tubular basophilia, focal, unilateral, minimal; Glomerular hyalinosis, multifocal, minimal, bilateral.<br>• Liver: Lymphocytic infiltrate, portal and centrilobular perivascular, multifocal, mild.<br>• Stomach: Mucosal infiltrate, lymphocytic and eosinophilic, multifocal, minimal.<br>• Mesenteric lymph node: Follicular lymphoid hyperplasia.<br>• Skin (trunk): Acanthosis and hyperkeratosis, moderate, diffuse, with chronic dermatitis, mild, multifocal.<br>• Spinal cord: Epidermoid cyst, meninges.<br>• Parathyroid, mammary glands, eyes, pituitary, ears: Not present on slide.<br>• Other: Abnormal mesenteric tissue observed grossly: Lymph node, with sinus ectasia and follicular lymphoid hyperplasia.

Table S6, cont'd. Single-dose toxicity testing in tumor-free female athymic nude mice 145 d after systemic administration of saline vehicle versus varying dose levels of [^{225}Ac]Pr.

#1 37 kBq	• Body weight is 24.470 g.<br>• No gross lesions are observed.	**All tissues are normal unless otherwise described.**<br>• Lungs: Peribronchiolar and perivascular lymphocytic infiltrate, mild, multifocal.<br>• Kidneys: Lymphocytic interstitial infiltrate, multifocal, bilateral, minimal.<br>• Liver: Lymphocytic infiltrate, portal and centrilobular perivascular, multifocal, mild.<br>• Mesenteric lymph node: Follicular lymphoid hyperplasia.<br>• Urinary bladder: Lymphocytic mucosal infiltrate, multifocal, mild.<br>• Skin (trunk): Acanthosis and hyperkeratosis, moderate, diffuse, with chronic dermatitis, mild, multifocal.<br>• Gallbladder, pituitary: Not present on slide.<br>• Other: Not applicable.
#2 37 kBq	• Body weight is 27.502 g.<br>• No gross lesions are observed.	**All tissues are normal unless otherwise described.**<br>• Lungs: Peribronchiolar and perivascular lymphoplasmacytic infiltrate, mild, multifocal.<br>• Liver: Lymphocytic infiltrate, portal and centrilobular perivascular, multifocal, mild; Random infiltrate, lymphocytic, histiocytic, neutrophilic, multifocal, minimal.<br>• Mesenteric lymph node: Plasmacytosis; Follicular lymphoid hyperplasia.<br>• Spleen: Follicular lymphoid hyperplasia.<br>• Skin (trunk): Acanthosis and hyperkeratosis, moderate, diffuse, with chronic dermatitis, mild, multifocal.<br>• Thyroid, parathyroid, ears: Not present on slide.<br>• Other: Not applicable.
#3 37 kBq	• Body weight is 24.034 g.<br>• No gross lesions are observed.	**All tissues are normal unless otherwise described.**<br>• Liver: Lymphocytic infiltrate, portal and centrilobular perivascular, multifocal, minimal; Random infiltrate, lymphocytic, histiocytic, neutrophilic, multifocal, minimal.<br>• Mesenteric lymph node: Follicular lymphoid hyperplasia.<br>• Skin (trunk): Acanthosis and hyperkeratosis, moderate, diffuse, with chronic dermatitis, mild, multifocal.<br>• Eyes: Blepharitis, pyogranulomatous, focal, unilateral, marked.<br>• Pituitary, ears: Not present on slide.<br>• Other: Not applicable.

Table S6, cont'd. Single-dose toxicity testing in tumor-free female athymic nude mice 145 d after systemic administration of saline vehicle versus varying dose levels of [^{225}Ac]Pr.

#4 37 kBq	• Body weight is 26.022 g.<br>• No gross lesions are observed.	**All tissues are normal unless otherwise described.**<br>• Lungs: Peribronchiolar and perivascular lymphocytic infiltrate, mild, multifocal.<br>• Liver: Lymphocytic infiltrate, portal and centrilobular perivascular, multifocal, minimal; Random infiltrate, lymphocytic, histiocytic, neutrophilic, multifocal, mild.<br>• Duodenum, jejunum, ileum: Mucosal and submucosal infiltrate, eosinophilic, diffuse, mild.<br>• Mesenteric lymph node: Plasmacytosis; Follicular lymphoid hyperplasia.<br>• Submandibular lymph node: Follicular lymphoid hyperplasia.<br>• Trachea: Mucosal lymphoplasmacytic infiltrate, mild, multifocal.<br>• Skin (trunk): Acanthosis and hyperkeratosis, moderate, diffuse, with chronic dermatitis, mild, multifocal.<br>• Eyes: Meibomian adenitis, pyogranmulomatousa, focal, unilateral, mild.<br>• Pituitary, ears: Not present on slide.<br>• Other: Not applicable.
#5 37 kBq	• Body weight is 25.292 g.<br>• No gross lesions are observed.	**All tissues are normal unless otherwise described.**<br>• Liver: Lymphocytic infiltrate, portal and centrilobular perivascular, multifocal, minimal; Random infiltrate, lymphocytic, histiocytic, neutrophilic, multifocal, minimal.<br>• Mesenteric lymph node: Plasmacytosis.<br>• Submandibular lymph node: Paracortical lymphoid depletion.<br>• Urinary bladder: Lymphocytic mucosal infiltrate, focal, mild.<br>• Spleen: Follicular lymphoid hyperplasia.<br>• Oviducts: Lymphocytic infiltrate, mild, focal.<br>• Skin (trunk): Acanthosis and hyperkeratosis, moderate, diffuse, with chronic dermatitis, mild, multifocal.<br>• Eyes: Meibomian adenitis, pyogranulomatousa, focal, unilateral, mild.<br>• Parathyroid, pituitary, ears: Not present on slide.<br>• Other: Not applicable.

Table S6, cont'd. Single-dose toxicity testing in tumor-free female athymic nude mice 145 d after systemic administration of saline vehicle versus varying dose levels of [^{225}Ac]Pr.

#1 74 kBq	• Body weight is 27.749 g.<br>• Spleen is enlarged; Mesenteric lymph node is enlarged.	**All tissues are normal unless otherwise described.**<br>• Liver: Lymphocytic infiltrate, portal and centrilobular perivascular, multifocal, minimal.<br>• Mesenteric lymph node: Plasmacytosis; Follicular lymphoid hyperplasia.<br>• Spleen: Follicular lymphoid hyperplasia.<br>• Adrenals: Cortical atrophy, with vacuolar degeneration, diffuse.<br>• Ovaries: Follicular atrophy, with vacuolation of interstitial cells, diffuse.<br>• Skin (trunk): Acanthosis and hyperkeratosis, moderate, diffuse, w/ chronic dermatitis, mild, multifocal.<br>• Harderian gland: Lymphocytic infiltrate, focal, unilateral, mild.<br>• Thymus, gallbladder, pituitary: Not present on slide.<br>Other: Not applicable.
#2 74 kBq	• Body weight is 31.008 g.<br>• Spleen is enlarged and has rough edges.	**All tissues are normal unless otherwise described.**<br>• Lungs: Peribronchiolar and perivascular lymphocytic infiltrate, mild, multifocal.<br>• Mesenteric lymph node: Plasmacytosis; Follicular lymphoid hyperplasia.<br>• Kidneys: Interstitial infiltrate, eosinophilic, histiocytic, and lymphocytic, multifocal, moderate, marked.<br>• Liver: Eosinophilic, histiocytic, and lymphocytic portal infiltrate, multifocal, marked; Hepatic necrosis, focal, mild.<br>• Mesenteric lymph node: Follicular lymphoid hyperplasia.<br>• Uterus: Granulomatous metritis, focal, mild.<br>• Urinary bladder: Lymphocytic mucosal infiltrate, multifocal, mild.<br>• Spleen: Splenitis, histiocytic, neutrophilic, and eosinophilic, with fibrosis, multifocal, moderate.<br>• Adrenals: Cortical atrophy, with vacuolar degeneration, diffuse.<br>• Ovaries: Follicular atrophy, with vacuolation of interstitial cells, diffuse.<br>• Skin (trunk): Acanthosis and hyperkeratosis, moderate, diffuse, with chronic dermatitis, mild, multifocal.<br>• Harderian gland: Lymphocytic infiltrate, focal, unilateral, mild.<br>• Submandibular lymph node, parathyroid, pituitary: Not present on slide.<br>• Other: Not applicable.
#3 74 kBq	• Body weight is 26.472 g.<br>• No gross lesions are observed.	**All tissues are normal unless otherwise described.**<br>• Liver: Lymphocytic infiltrate, portal and centrilobular perivascular, multifocal, minimal.<br>• Mesenteric lymph node: Plasmacytosis; Follicular lymphoid hyperplasia.<br>• Spleen: Follicular lymphoid hyperplasia.<br>• Skin (trunk): Acanthosis and hyperkeratosis, moderate, diffuse, with chronic dermatitis, mild, multifocal.<br>• Ears: Otitis media, neutrophilic and histiocytic, moderate, unilateral.<br>• Colon, parathyroid: Not present on slide.<br>• Other: Not applicable.
#4 74 kBq	• Body weight is 24.739 g.<br>• No gross lesions are observed.	**All tissues are normal unless otherwise described.**<br>• Liver: Lymphocytic infiltrate, portal and centrilobular perivascular, multifocal, minimal.<br>• Trachea: Mucosal squamous metaplasia, mild, focal.<br>• Skin (trunk): Acanthosis and hyperkeratosis, moderate, diffuse, with chronic dermatitis, mild, multifocal.<br>• Parathyroid, pituitary, ears: Not present on slide.<br>• Other: Not applicable.

Table S6, cont'd. Single-dose toxicity testing in tumor-free female athymic nude mice 145 d after systemic administration of saline vehicle versus varying dose levels of [^{225}Ac]Pr.

#5 74 kBq	• Body weight is 27.880 g.<br>• No gross lesions are observed.	**All tissues are normal unless otherwise described.**<br>• Heart: Epicardial lymphocytic infiltrate, left ventricular and atrial walls, minimal, multifocal.<br>• Lungs: Peribronchiolar and perivascular lymphocytic infiltrate, mild, multifocal.<br>• Liver: Lymphocytic infiltrate, portal and centrilobular perivascular, multifocal, moderate.<br>• Duodenum, jejunum, ileum: Mucosal and submucosal infiltrate, eosinophilic, segmental, diffuse, mild.<br>• Mesenteric lymph node: Follicular lymphoid hyperplasia.<br>• Urinary bladder: Lymphocytic mucosal infiltrate, focal, minimal.<br>• Skin (trunk): Acanthosis and hyperkeratosis, moderate, diffuse, with chronic dermatitis, mild, multifocal.<br>• Eyes: Keratitis, neutrophilic, with stromal vascularization, diffuse, bilateral, moderate.<br>• Parathyroid, pituitary, ears: Not present on slide.<br>• Other: Not applicable.

#1 148 kBq	• Body weight is 28.106 g.<br>• No gross lesions are observed.	**All tissues are normal unless otherwise described.**<br>• Kidney: Glomerular hyalinosis, multifocal, minimal, bilateral.<br>• Liver: Lymphocytic infiltrate, portal and centrilobular perivascular, multifocal, minimal.<br>• Stomach: Mucosal infiltrate, lymphocytic and eosinophilic, multifocal, mild.<br>• Submandibular lymph node: Follicular lymphoid hyperplasia.<br>• Urinary bladder: Lymphocytic mucosal infiltrate, focal, minimal.<br>• Skin (trunk): Acanthosis and hyperkeratosis, moderate, diffuse, with chronic dermatitis, mild, multifocal.<br>• Ears: Otitis media, neutrophilic and histiocytic, moderate, unilateral.<br>• Parathyroid: Not present on slide.<br>• Other: Not applicable.
#2 148 kBq	• Body weight is 25.213 g.<br>• Cyst-like lesion adjacent to liver, 7 x 3 x 2 mm.	**All tissues are normal unless otherwise described.**<br>• Liver: Lymphocytic infiltrate, portal and centrilobular perivascular, multifocal, moderate; Random infiltrate, lymphocytic, histiocytic, neutrophilic, with hepatocyte apoptosis, multifocal, minimal.<br>• Mesenteric lymph node: Follicular lymphoid hyperplasia; Sinus ectasia.<br>• Thyroid: Follicular cyst, focal.<br>• Skin (trunk): Acanthosis and hyperkeratosis, moderate, diffuse, with chronic dermatitis, mild, multifocal.<br>• Parathyroid: Not present on slide.<br>• Other: Cyst-like lesion adjacent to liver observed grossly: Lymph node, with sinus ectasia.

Table S6, cont'd. Single-dose toxicity testing in tumor-free female athymic nude mice 145 d after systemic administration of saline vehicle versus varying dose levels of [^{225}Ac]Pr.

#3 148 kBq	• Body weight is 25.540 g.<br>• Spleen focally adhered to stomach; Left uterine horn dilated, 4 mm in diameter.	**All tissues are normal unless otherwise described.**<br>• Heart: Histiocytic myocardial infiltrate, right ventricular wall, focal, minimal.<br>• Liver: Lymphocytic infiltrate, portal and centrilobular perivascular, multifocal, mild; Random infiltrate, lymphocytic, histiocytic, neutrophilic, with hepatocyte apoptosis, multifocal, minimal.<br>• Uterus: Luminal dilation, moderate, diffuse.<br>• Urinary bladder: Lymphocytic mucosal infiltrate, focal, minimal.<br>• Skin (trunk): Acanthosis and hyperkeratosis, moderate, diffuse, with chronic dermatitis, mild, multifocal.<br>• Harderian gland: Glandular hyperplasia, focal, unilateral, mild.<br>• Mesenteric lymph node, parathyroid: Not present on slide.<br>• Other: Not applicable.
#4 148 kBq	• Body weight is 24.246 g.<br>• No gross lesions are observed.	**All tissues are normal unless otherwise described.**<br>• Kidneys: Tubular basophilia, focal, unilateral, minimal.<br>• Liver: Lymphocytic infiltrate, portal and centrilobular perivascular, multifocal, moderate; Random infiltrate, lymphocytic, histiocytic, neutrophilic, multifocal, minimal.<br>• Mesenteric lymph node: Plasmacytosis; Follicular lymphoid hyperplasia.<br>• Urinary bladder: Lymphocytic mucosal infiltrate, focal, minimal.<br>• Pancreas: Exocrine atrophy, marked, diffuse.<br>• Skin (trunk): Acanthosis and hyperkeratosis, moderate, diffuse, with chronic dermatitis, mild, multifocal.<br>• Parathyroid: Not present on slide.<br>• Other: Not applicable.
#5 148 kBq	• Body weight is 25.506 g.<br>• No gross lesions are observed.	**All tissues are normal unless otherwise described.**<br>• Lungs: Neutrophilic and histiocytic pneumonia, minimal, focal.<br>• Kidneys: Tubular basophilia, multifocal, unilateral, minimal.<br>• Liver: Eosinophilic, histiocytic, and lymphocytic portal infiltrate, multifocal, marked.<br>• Stomach: Mucosal infiltrate, lymphocytic and eosinophilic, multifocal, moderate.<br>• Mesenteric lymph node: Follicular lymphoid hyperplasia.<br>• Thyroid: Follicular cyst, focal.<br>• Skin (trunk): Acanthosis and hyperkeratosis, moderate, diffuse, with chronic dermatitis, mild, multifocal.<br>• Pituitary: Not present on slide.<br>• Other: Not applicable.

Table S6, cont'd. Single-dose toxicity testing in tumor-free female athymic nude mice 145 d after systemic administration of saline vehicle versus varying dose levels of [^{225}Ac]Pr.

#1 296 kBq	• Body weight is 26.783 g.<br>• No gross lesions are observed.	**All tissues are normal unless otherwise described.**<br>• Kidneys: Tubular degeneration and atrophy, cortical, multifocal, bilateral, mild.<br>• Liver: Lymphocytic infiltrate, portal and centrilobular perivascular, multifocal, minimal.<br>• Stomach: Mucosal infiltrate, lymphocytic, segmental, multifocal, mild.<br>• Mesenteric lymph node: Plasmacytosis; Sinus ectasia.<br>• Skin (trunk): Acanthosis and hyperkeratosis, moderate, diffuse, with chronic dermatitis, mild, multifocal.<br>• Vagina, pituitary, ears: Not present on slide.<br>• Other: Not applicable.
#2 296 kBq	• Body weight is 23.875 g.<br>• No gross lesions are observed.	**All tissues are normal unless otherwise described.**<br>• Kidneys: Tubular degeneration and atrophy, cortical, multifocal, bilateral, mild.<br>• Liver: Lymphocytic infiltrate, portal and centrilobular perivascular, multifocal, mild.<br>• Duodenum, jejunum, ileum: Mucosal and submucosal infiltrate, eosinophilic, segmental, diffuse, mild.<br>• Mesenteric lymph node: Follicular lymphoid hyperplasia.<br>• Submandibular lymph node: Follicular lymphoid hyperplasia.<br>• Urinary bladder: Lymphocytic and eosinophilic mucosal infiltrate, multifocal, minimal.<br>• Spleen: Follicular lymphoid hyperplasia.<br>• Skin (trunk): Acanthosis and hyperkeratosis, moderate, diffuse, with chronic dermatitis, mild, multifocal.<br>• Parathyroid, mammary glands, pituitary, ears: Not present on slide.<br>• Other: Not applicable.
#3 296 kBq	• Body weight is 28.403 g.<br>• No gross lesions are observed.	**All tissues are normal unless otherwise described.**<br>• Lungs: Peribronchiolar and perivascular lymphocytic infiltrate, minimal, multifocal.<br>• Liver: Lymphocytic infiltrate, portal and centrilobular perivascular, multifocal, moderate.<br>• Stomach: Mucosal infiltrate, lymphocytic, segmental, multifocal, mild.<br>• Mesenteric lymph node: Plasmacytosis; Follicular lymphoid hyperplasia.<br>• Urinary bladder: Cystitis, eosinophilic, neutrophilic, and lymphocytic, with mucosal necrosis and fibrosis, and urothelial hyperplasia, marked, diffuse.<br>• Spleen: Follicular lymphoid hyperplasia.<br>• Skin (trunk): Acanthosis and hyperkeratosis, moderate, diffuse, with chronic dermatitis, mild, multifocal.<br>• Parathyroid: Not present on slide.<br>• Other: Not applicable.

Table S6, cont'd. Single-dose toxicity testing in tumor-free female athymic nude mice 145 d after systemic administration of saline vehicle versus varying dose levels of [^{225}Ac]Pr.

#4 296 kBq	• Body weight is 25.149 g.<br>• No gross lesions are observed.	**All tissues are normal unless otherwise described.**<br>• Liver: Lymphocytic infiltrate, portal and centrilobular perivascular, multifocal, minimal.<br>• Duodenum, jejunum, ileum: Mucosal and submucosa infiltrate, eosinophilic, segmental, diffuse, mild.<br>• Urinary bladder: Lymphocytic mucosal infiltrate, focal, minimal.<br>• Spleen: Splenitis, histiocytic, neutrophilic, and eosinophilic, with fibrosis, multifocal, moderate.<br>• Skin (trunk): Acanthosis and hyperkeratosis, moderate, diffuse, with chronic dermatitis, mild, multifocal.<br>• Thymus, mesenteric lymph node, parathyroid: Not present on slide.<br>• Other: Not applicable.

Table S7. Complete automated differential blood counts of tumor-free female athymic nude mice 145 d after systemic administration of saline vehicle versus varying dose levels of [^{225}Ac]Pr.

Test Name	#1 Saline	#2 Saline	#3 Saline	#4 Saline	#5 Saline	#1 9.25 kBq	#2 9.25 kBq	#3 9.25 kBq	#4 9.25 kBq	#5 9.25 kBq	#1 18.5 kBq	#2 18.5 kBq	#3 18.5 kBq
WBC# (K/uL)	12.86	5.07	5.18	7.29	9.17	9.88	8.67	4.08	6.26	4.82	2.87	6.29	5.55
NEUT# (K/uL)	2.69	1.54	1.36	1.67	3.45	1.76	2.17	0.62	1.59	1.21	1.03	1.57	1.17
LYMPH# (K/uL)	9.31	3.22	3.40	4.67	5.15	7.40	5.88	3.24	4.22	3.17	1.70	4.30	3.98
MONO# (K/uL)	0.54	0.13	0.24	0.45	0.32	0.48	0.29	0.11	0.27	0.32	0.04	0.24	0.25
EO# (K/uL)	0.32	0.16	0.16	0.48	0.24	0.23	0.32	0.11	0.17	0.12	0.09	0.17	0.14
BASO# (K/uL)	0.00	0.02	0.02	0.02	0.01	0.01	0.01	0.00	0.01	0.00	0.01	0.01	0.01
NEUT (%)	20.9	30.3	26.3	22.8	37.6	17.8	25.1	15.2	25.4	25.1	36.0	24.9	21.1
LYMPH (%)	72.4	63.5	65.6	64.1	56.2	74.9	67.8	79.4	67.4	65.8	59.2	68.4	71.7
MONO (%)	4.2	2.6	4.6	6.2	3.5	4.9	3.3	2.7	4.3	6.6	1.4	3.8	4.5
EO (%)	2.5	3.2	3.1	6.6	2.6	2.3	3.7	2.7	2.7	2.5	3.1	2.7	2.5
BASO (%)	0.0	0.4	0.4	0.3	0.1	0.1	0.1	0.0	0.2	0.0	0.3	0.2	0.2

Test Name	#1 37 kBq	#2 37 kBq	#3 37 kBq	#4 37 kBq	#5 37 kBq	#1 74 kBq	#2 74 kBq	#3 74 kBq	#4 74 kBq	#5 74 kBq	#1 148 kBq	#2 148 kBq	#3 148 kBq	#4 148 kBq	#5 148 kBq
WBC# (K/uL)	11.17	9.83	10.91	6.39	3.98	4.05	4.10	3.91	4.19	9.65	4.56	7.38	4.63	6.42	5.00
NEUT# (K/uL)	2.47	2.04	3.74	1.81	1.21	0.88	1.09	1.75	1.82	2.83	1.30	2.55	1.27	1.62	1.31
LYMPH# (K/uL)	8.05	7.37	6.63	4.05	2.52	2.73	2.47	1.67	2.17	6.57	2.68	4.18	2.67	4.24	3.17
MONO# (K/uL)	0.49	0.26	0.46	0.35	0.16	0.29	0.46	0.40	0.07	0.14	0.17	0.44	0.55	0.35	0.37
EO# (K/uL)	0.13	0.14	0.06	0.18	0.08	0.15	0.07	0.08	0.11	0.09	0.38	0.20	0.12	0.20	0.14
BASO# (K/uL)	0.03	0.02	0.02	0.00	0.01	0.00	0.01	0.01	0.02	0.02	0.03	0.01	0.02	0.01	0.01
NEUT (%)	22.0	20.8	34.3	28.3	30.4	21.7	26.7	44.8	43.4	29.3	28.5	34.6	27.4	25.2	26.2
LYMPH (%)	72.1	75.0	60.8	63.4	63.3	67.4	60.2	42.7	51.8	68.1	58.8	56.6	57.7	66.0	63.4
MONO (%)	4.4	2.6	4.2	5.5	4.0	7.2	11.2	10.2	1.7	1.5	3.7	6.0	11.9	5.5	7.4
EO (%)	1.2	1.4	0.5	2.8	2.0	3.7	1.7	2.0	2.6	0.9	8.3	2.7	2.6	3.1	2.8
BASO (%)	0.3	0.2	0.2	0.0	0.3	0.0	0.2	0.3	0.5	0.2	0.7	0.1	0.4	0.2	0.2

WBC: White blood cell; NEUT: Neutrophils; LYMPH: Lymphocytes; MONO: Monocytes; EO: Eosinophils; BASO: Basophils

Table S7, cont'd. Complete automated differential blood counts of tumor-free female athymic nude mice 145 d after systemic administration of saline vehicle versus varying dose levels of [^{225}Ac]Pr.

Test Name	#1 296 kBq	#2 296 kBq	#3 296 kBq	#4 296 kBq
WBC# (K/uL)	5.58	8.20	6.67	6.11
NEUT# (K/uL)	2.14	1.10	1.53	1.35
LYMPH# (K/uL)	3.00	6.61	4.70	4.39
MONO# (K/uL)	0.29	0.38	0.30	0.18
EO# (K/uL)	0.15	0.11	0.14	0.18
BASO# (K/uL)	0.00	0.00	0.00	0.01
NEUT (%)	38.3	13.5	22.9	22.2
LYMPH (%)	53.8	80.6	70.5	71.8
MONO (%)	5.2	4.6	4.5	2.9
EO (%)	2.7	1.3	2.1	2.9
BASO (%)	0.0	0.0	0.0	0.2

WBC: White blood cell; NEUT: Neutrophils; LYMPH: Lymphocytes; MONO: Monocytes; EO: Eosinophils; BASO: Basophils

Table S8. Complete manual differential blood counts of tumor-free female athymic nude mice 145 d after systemic administration of saline vehicle versus varying dose levels of [^{225}Ac]Pr.

Test Name	#1 Saline	#2 Saline	#3 Saline	#4 Saline	#5 Saline
NEUT# (K/uL)	3.73	2.23	2.38	3.57	5.04
Band# (K/uL)	1.54	0.71	0.62	0.15	0.55
LYMPH# (K/uL)	6.30	1.83	1.14	2.55	2.48
MONO# (K/uL)	0.39	0.00	0.10	0.00	0.09
EO# (K/uL)	0.64	0.30	0.73	0.80	0.46
NEUT (%)	29.0	44.0	46.0	49.0	55.0
Band (%)	12.0	14.0	12.0	2.0	6.0
LYMPH (%)	49.0	36.0	22.0	35.0	27.0
MONO (%)	3.0	0.0	2.0	0.0	1.0
Other 1 (%)	5.0	6.0	14.0	11.0	5.0
Other 2 (%)	0.0	0.0	2.0	2.0	4.0
EO (%)	2.0	0.0	2.0	1.0	2.0
Morphology	3+ reactive lymphocytes. 4+ fragile WBCs.	3+ reactive lymphocytes. 4+ fragile WBCs.	3+ reactive lymphocytes. 4+ fragile WBCs.	3+ reactive lymphocytes. 4+ fragile WBCs.	3+ reactive lymphocytes. 4+ fragile WBCs.

Test Name	#1 9.25 kBq	#2 9.25 kBq	#3 9.25 kBq	#4 9.25 kBq	#5 9.25 kBq
NEUT# (K/uL)	2.07	3.55	0.57	2.88	2.02
Band# (K/uL)	0.79	0.52	0.41	0.88	0.48
LYMPH# (K/uL)	6.82	3.81	3.02	1.94	2.12
MONO# (K/uL)	0.00	0.00	0.00	0.00	0.00
EO# (K/uL)	0.10	0.26	0.08	0.31	0.10
NEUT (%)	21.0	41.0	14.0	46.0	42.0
Band (%)	8.0	6.0	10.0	14.0	10.0
LYMPH (%)	69.0	44.0	74.0	31.0	44.0
MONO (%)	0.0	0.0	0.0	0.0	0.0
Other 1 (%)	1.0	3.0	2.0	5.0	2.0
Other 2 (%)	1.0	3.0	0.0	3.0	2.0
EO (%)	0.0	3.0	0.0	1.0	0.0
Morphology	3+ reactive lymphocytes. 4+ fragile WBCs. Macrophage seen.	2+ reactive lymphocytes. 4+ fragile WBCs.	3+ reactive lymphocytes. 4+ fragile WBCs.	3+ reactive lymphocytes. 4+ fragile WBCs.	3+ reactive lymphocytes. 4+ fragile WBCs.

NEUT: Neutrophils; LYMPH: Lymphocytes; MONO: Monocytes; EO: Eosinophils; Other 1: Moderate to large mononuclear cells, with high nucleus to cytoplasm ratio, coarse to clumped chromatin, moderate to marked basophilic cytoplasm, and occasionally vacuolated. Other 2: Large mononuclear cells, with high nucleus to cytoplasm ratio, lacy to coarse, and pale blue cytoplasm.

Table S8, cont'd. Complete manual differential blood counts of tumor-free female athymic nude mice 145 d after systemic administration of saline vehicle versus varying dose levels of [^{225}Ac]Pr.

Test Name	#1 18.5 kBq	#2 18.5 kBq	#3 18.5 kBq
NEUT# (K/uL)	0.69	2.39	3.22
Band# (K/uL)	0.23	0.31	0.33
LYMPH# (K/uL)	1.72	3.02	1.33
MONO# (K/uL)	0.00	0.00	0.00
EO# (K/uL)	0.11	0.50	0.56
NEUT (%)	24.0	38.0	58.0
Band (%)	8.0	5.0	6.0
LYMPH (%)	60.0	48.0	24.0
MONO (%)	0.0	0.0	0.0
Other 1 (%)	4.0	8.0	10.0
Other 2 (%)	2.0	1.0	2.0
EO (%)	2.0	0.0	0.0
Morphology	3+ reactive lymphocytes. 4+ fragile WBCs.	3+ reactive lymphocytes. 4+ fragile WBCs.	3+ reactive lymphocytes. 4+ fragile WBCs. Count performed with 50 cells.

Test Name	#1 37 kBq	#2 37 kBq	#3 37 kBq	#4 37 kBq	#5 37 kBq
NEUT# (K/uL)	3.35	2.06	6.66	2.04	1.19
Band# (K/uL)	0.00	0.00	0.00	0.00	0.00
LYMPH# (K/uL)	6.70	6.49	3.60	3.83	2.63
MONO# (K/uL)	0.00	0.00	0.00	0.13	0.00
EO# (K/uL)	0.22	0.20	0.44	0.19	0.16
NEUT (%)	30.0	21.0	61.0	32.0	30.0
Band (%)	0.0	0.0	0.0	0.0	0.0
LYMPH (%)	60.0	66.0	33.0	60.0	66.0
MONO (%)	0.0	0.0	0.0	2.0	0.0
Other 1 (%)	2.0	2.0	4.0	3.0	4.0
Other 2 (%)	7.0	10.0	0.0	2.0	0.0
EO (%)	1.0	1.0	2.0	1.0	0.0
Morphology	2+ reactive lymphocytes. 4+ fragile WBCs.	2+ reactive lymphocytes. 4+ fragile WBCs.	2+ reactive lymphocytes. 4+ fragile WBCs.	2+ reactive lymphocytes. 4+ fragile WBCs.	4+ reactive lymphocytes. 4+ fragile WBCs.

NEUT: Neutrophils; LYMPH: Lymphocytes; MONO: Monocytes; EO: Eosinophils; Other 1: Moderate to large mononuclear cells, with high nucleus to cytoplasm ratio, coarse to clumped chromatin, moderate to marked basophilic cytoplasm, and occasionally vacuolated. Other 2: Large mononuclear cells, with high nucleus to cytoplasm ratio, lacy to coarse, and pale blue cytoplasm.

Table S8, cont'd. Complete manual differential blood counts of tumor-free female athymic nude mice 145 d after systemic administration of saline vehicle versus varying dose levels of [^{225}Ac]Pr.

Test Name	#1 74 kBq	#2 74 kBq	#3 74 kBq	#4 74 kBq	#5 74 kBq
Neut# (K/uL)	1.46	1.15	1.72	2.01	4.34
Band# (K/uL)	0.00	0.00	0.00	0.00	0.00
LYMPH# (K/uL)	2.35	2.54	1.64	1.84	4.63
MONO# (K/uL)	0.00	0.00	0.00	0.00	0.00
EO# (K/uL)	0.08	0.00	0.16	0.17	0.10
NEUT (%)	36.0	28.0	44.0	48.0	45.0
Band (%)	0.0	0.0	0.0	0.0	0.0
LYMPH (%)	58.0	62.0	42.0	44.0	48.0
MONO (%)	0.0	0.0	0.0	0.0	0.0
Other 1 (%)	2.0	0.0	4.0	4.0	1.0
Other 2 (%)	0.0	2.0	2.0	4.0	4.0
EO (%)	4.0	8.0	8.0	0.0	2.0
Morphology	2+ reactive lymphocytes. 4+ fragile WBCs. Count performed with 50 cells.	2+ reactive lymphocytes. 4+ fragile WBCs.	2+ reactive lymphocytes. 4+ fragile WBCs.	2+ reactive lymphocytes. 4+ fragile WBCs.	2+ reactive lymphocytes. 4+ fragile WBCs.

Test Name	#1 148 kBq	#2 148 kBq	#3 148 kBq	#4 148 kBq	#5 148 kBq
Neut# (K/uL)	1.37	3.69	1.67	1.80	1.80
Band# (K/uL)	0.00	0.15	0.00	0.00	0.10
LYMPH# (K/uL)	2.55	2.29	1.67	3.47	2.30
MONO# (K/uL)	0.18	0.59	0.56	0.19	0.00
EO# (K/uL)	0.46	0.30	0.28	0.45	0.50
NEUT (%)	30.0	50.0	36.0	28.0	36.0
Band (%)	0.0	2.0	0.0	0.0	2.0
LYMPH (%)	56.0	31.0	36.0	54.0	46.0
MONO (%)	4.0	8.0	12.0	3.0	0.0
Other 1 (%)	10.0	4.0	6.0	7.0	10.0
Other 2 (%)	0.0	5.0	9.0	5.0	4.0
EO (%)	0.0	0.0	1.0	3.0	2.0
Morphology	3+ reactive lymphocytes. 4+ fragile WBCs. Count performed with 50 cells.	2+ reactive lymphocytes. 4+ fragile WBCs. Count performed with 50 cells.	+ reactive lymphocytes. 4+ fragile WBCs. Count performed with 50 cells.	2+ reactive lymphocytes. 4+ fragile WBCs. Count performed with 50 cells.	2+ reactive lymphocytes. 4+ fragile WBCs. Count performed with 50 cells.

NEUT: Neutrophils; LYMPH: Lymphocytes; MONO: Monocytes; EO: Eosinophils; Other 1: Moderate to large mononuclear cells, with high nucleus to cytoplasm ratio, coarse to clumped chromatin, moderate to marked basophilic cytoplasm, and occasionally vacuolated. Other 2: Large mononuclear cells, with high nucleus to cytoplasm ratio, lacy to coarse, and pale blue cytoplasm.

Table S8, cont'd. Complete manual differential blood counts of tumor-free female athymic nude mice 145 d after systemic administration of saline vehicle versus varying dose levels of [^{225}Ac]Pr.

Test Name	#1 296 kBq	#2 296 kBq	#3 296 kBq	#4 296 kBq
Neut# (K/uL)	3.35	2.79	2.40	2.26
Band# (K/uL)	0.00	0.90	0.67	0.43
LYMPH# (K/uL)	1.84	4.18	3.07	2.93
MONO# (K/uL)	0.11	0.16	0.20	0.00
EO# (K/uL)	0.28	0.08	0.27	0.24
NEUT (%)	60.0	34.0	36.0	37.0
Band (%)	0.0	11.0	10.0	7.0
LYMPH (%)	33.0	51.0	46.0	48.0
MONO (%)	2.0	2.0	3.0	0.0
Other 1 (%)	5.0	1.0	4.0	4.0
Other 2 (%)	0.0	0.0	1.0	4.0
EO (%)	0.0	1.0	0.0	0.0
Morphology	3+ reactive lymphocytes. 4+ fragile WBCs.	3+ reactive lymphocytes. 4+ fragile WBCs.	3+ reactive lymphocytes. 4+ fragile WBCs.	3+ reactive lymphocytes. 4+ fragile WBCs.

NEUT: Neutrophils; LYMPH: Lymphocytes; MONO: Monocytes; EO: Eosinophils; Other 1: Moderate to large mononuclear cells, with high nucleus to cytoplasm ratio, coarse to clumped chromatin, moderate to marked basophilic cytoplasm, and occasionally vacuolated. Other 2: Large mononuclear cells, with high nucleus to cytoplasm ratio, lacy to coarse, and pale blue cytoplasm.

Table S9. Complete blood counts of tumor-free female athymic nude mice 145 d after systemic administration of saline vehicle versus varying dose levels of [^{225}Ac]Pr.

Test Name	#1 Saline	#2 Saline	#3 Saline	#4 Saline	#5 Saline
RBC (M/uL)	9.53	9.57	8.48	9.22	8.34
HGB (g/dL)	14.5	14.4	13.7	14.5	12.8
HCT (%)	48.1	48.4	44.5	47.2	41.6
MCV (fL)	50.5	50.6	52.5	51.2	49.9
MCH (pg)	15.2	15.0	16.2	15.7	15.3
MCHC (g/dL)	30.1	29.8	30.8	30.7	30.8
RDW-SD (fL)	32.5	30.1	32.6	27.0	30.1
RDW-CV (%)	24.3	23.0	22.5	21.4	22.2
RET# (K/uL)	419.3	395.2	285.8	452.7	312.8
RET (%)	4.40	4.13	3.37	4.91	3.75
PLT (K/uL)	756	193	745	915	1249
PDW (fL)	6.6	7.1	6.3	6.9	6.4
MPV (fL)	5.9	6.3	5.9	6.1	5.8
Morphology	3+ Polychromasia. RBC morphology within normal limits	3+ Polychromasia. RBC morphology within normal limits	3+ Polychromasia. RBC morphology within normal limits	3+ Polychromasia. RBC morphology within normal limits	3+ Polychromasia. RBC morphology within normal limits

RBC: Red blood cell count; HGB: hemoglobin; HCT: Hematocrit; MCV: Mean corpuscular volume; MCH: Mean Corpuscular Hemoglobin; MCHC: Mean corpuscular hemoglobin concentration; RDW: Red blood cell distribution width; RET: Reticulocyte; PLT: Platelets; PDW: Platelet Distribution Width; MPV: Mean platelet volume

Table S9, cont'd. Complete blood counts of tumor-free female athymic nude mice 145 d after systemic administration of saline vehicle versus varying dose levels of [^{225}Ac]Pr.

Test Name	#1 9.25 kBq	#2 9.25 kBq	#3 9.25 kBq	#4 9.25 kBq	#5 9.25 kBq
RBC (M/uL)	9.26	9.35	7.41	9.14	8.70
HGB (g/dL)	13.4	14.0	13.2	14.7	13.9
HCT (%)	44.1	46.9	42.4	47.8	45.7
MCV (fL)	47.6	50.2	57.2	52.3	52.5
MCH (pg)	14.5	15.0	17.8	16.1	16.0
MCHC (g/dL)	30.4	29.9	31.1	30.8	30.4
RDW-SD (fL)	29.5	29.7	36.7	28.8	30.0
RDW-CV (%)	23.6	22.8	21.7	21.8	21.5
RET# (K/uL)	327.8	326.3	442.4	383.9	255.8
RET (%)	3.54	3.49	5.97	4.20	2.94
PLT (K/uL)	990	768	622	933	699
PDW (fL)	6.3	6.1	6.6	6.5	6.7
MPV (fL)	5.6	5.7	6.1	5.8	6.0
Morphology	3+ Polychromasia. RBC morphology within normal limits	3+ Polychromasia. RBC morphology within normal limits	3+ Polychromasia. 4+ PLT clumps. RBC morphology within normal limits	3+ Polychromasia. 4+ PLT clumps. RBC morphology within normal limits	3+ Polychromasia. RBC morphology within normal limits

Test Name	#1 18.5 kBq	#2 18.5 kBq	#3 18.5 kBq
RBC (M/uL)	9.35	8.96	9.50
HGB (g/dL)	14.3	14.4	15.3
HCT (%)	47.3	46.4	50.1
MCV (fL)	50.6	51.8	52.7
MCH (pg)	15.3	16.1	16.1
MCHC (g/dL)	30.2	31.0	30.5
RDW-SD (fL)	29.5	28.3	29.2
RDW-CV (%)	22.5	21.5	21.6
RET# (K/uL)	260.9	370.0	336.3
RET (%)	2.79	4.13	3.54
PLT (K/uL)	939	818	875
PDW (fL)	7.1	6.6	6.5
MPV (fL)	6.2	6.0	5.9
Morphology	3+ Polychromasia. RBC morphology within normal limits	3+ Polychromasia. RBC morphology within normal limits	3+ Polychromasia. RBC morphology within normal limits

RBC: Red blood cell count; HGB: hemoglobin; HCT: Hematocrit; MCV: Mean corpuscular volume; MCH: Mean Corpuscular Hemoglobin; MCHC: Mean corpuscular hemoglobin concentration; RDW: Red blood cell distribution width; RET: Reticulocyte; PLT: Platelets; PDW: Platelet Distribution Width; MPV: Mean platelet volume

Table S9, cont'd. Complete blood counts of tumor-free female athymic nude mice 145 d after systemic administration of saline vehicle versus varying dose levels of [^{225}Ac]Pr.

Test Name	#1 37 kBq	#2 37 kBq	#3 37 kBq	#4 37 kBq	#5 37 kBq
RBC (M/uL)	9.22	8.50	9.05	9.64	9.23
HGB (g/dL)	13.7	13.6	14.2	14.0	13.5
HCT (%)	44.9	43.9	46.6	46.2	43.9
MCV (fL)	48.7	51.6	51.5	47.9	47.6
MCH (pg)	14.9	16.0	15.7	14.5	14.6
MCHC (g/dL)	30.5	31.0	30.5	30.3	30.8
RDW-SD (fL)	30.9	30.8	29.3	30.8	29.2
RDW-CV (%)	23.9	22.4	22.1	24.5	23.3
RET# (K/uL)	229.6	430.1	453.4	454.0	368.3
RET (%)	2.49	5.06	5.01	4.71	3.99
PLT (K/uL)	267	799	776	1147	785
PDW (fL)	8.4	6.6	6.9	6.2	6.7
MPV (fL)	6.6	6.0	6.4	5.5	5.9
Morphology	2+ Polychromasia. RBC morphology within normal limits	3+ Polychromasia. 4+ PLT clumps. RBC morphology within normal limits	3+ Polychromasia. 2+ PLT clumps. RBC morphology within normal limits	3+ Polychromasia. 3+ PLT clumps. RBC morphology within normal limits	4+ Polychromasia. 4+ PLT clumps RBC morphology within normal limits

Test Name	#1 74 kBq	#2 74 kBq	#3 74 kBq	#4 74 kBq	#5 74 kBq
RBC (M/uL)	7.87	9.03	8.66	8.83	8.98
HGB (g/dL)	12.2	14.1	14.3	14.5	14.3
HCT (%)	40.1	46.3	46.0	46.8	45.9
MCV (fL)	51.0	51.3	53.1	53.0	51.1
MCH (pg)	15.5	15.6	16.5	16.4	15.9
MCHC (g/dL)	30.4	30.5	31.1	31.0	31.2
RDW-SD (fL)	29.3	29.2	29.4	28.8	28.3
RDW-CV (%)	24.0	22.2	21.1	20.8	21.8
RET# (K/uL)	722.5	378.4	323.9	306.4	366.4
RET (%)	9.18	4.19	3.74	3.47	4.08
PLT (K/uL)	985	845	682	746	856
PDW (fL)	6.6	6.9	6.7	6.6	6.4
MPV (fL)	6.0	6.2	6.1	5.9	6.0
Morphology	4+ Polychromasia. RBC morphology within normal limits	3+ Polychromasia. RBC morphology within normal limits	2+ Polychromasia. RBC morphology within normal limits	2+ Polychromasia. RBC morphology within normal limits	3+ Polychromasia. RBC morphology within normal limits

RBC: Red blood cell count; HGB: hemoglobin; HCT: Hematocrit; MCV: Mean corpuscular volume; MCH: Mean Corpuscular Hemoglobin; MCHC: Mean corpuscular hemoglobin concentration; RDW: Red blood cell distribution width; RET: Reticulocyte; PLT: Platelets; PDW: Platelet Distribution Width; MPV: Mean platelet volume

Table S9, cont'd. **Table S9, cont'd.** Complete blood counts of tumor-free female athymic nude mice 145 d after systemic administration of saline vehicle versus varying dose levels of [^{225}Ac]Pr.

Test Name	#1 148 kBq	#2 148 kBq	#3 148 kBq	#4 148 kBq	#5 148 kBq
RBC (M/uL)	9.37	8.83	9.11	9.06	9.02
HGB (g/dL)	14.5	12.9	14.1	13.4	13.8
HCT (%)	46.5	43.5	46.2	43.1	44.3
MCV (fL)	49.6	49.3	50.7	47.6	49.1
MCH (pg)	15.5	14.6	15.5	14.8	15.3
MCHC (g/dL)	31.2	29.7	30.5	31.1	31.2
RDW-SD (fL)	29.7	29.4	28.2	27.9	29.9
RDW-CV (%)	22.8	22.3	21.7	22.8	23.2
RET# (K/uL)	208.0	317.0	300.6	285.4	337.3
RET (%)	2.22	3.59	3.30	3.15	3.74
PLT (K/uL)	731	1163	492	901	860
PDW (fL)	6.3	6.2	7.4	6.4	6.5
MPV (fL)	5.9	5.7	6.5	5.8	5.9
Morphology	2+ Polychromasia. RBC morphology within normal limits	2+ Polychromasia. RBC morphology within normal limits	2+ Polychromasia. 4+ PLT clumps. RBC morphology within normal limits	2+ Polychromasia. RBC morphology within normal limits	2+ Polychromasia. RBC morphology within normal limits

Test Name	#1 296 kBq	#2 296 kBq	#3 296 kBq	#4 296 kBq
RBC (M/uL)	8.37	8.14	8.75	8.17
HGB (g/dL)	13.3	12.4	13.7	12.3
HCT (%)	42.4	40.4	43.7	40.0
MCV (fL)	50.7	49.6	49.9	49.0
MCH (pg)	15.9	15.2	15.7	15.1
MCHC (g/dL)	31.4	30.7	31.4	30.8
RDW-SD (fL)	27.3	30.6	29.2	30.3
RDW-CV (%)	20.4	22.4	22.0	22.4
RET# (K/uL)	323.9	338.6	288.8	291.7
RET (%)	3.87	4.16	3.30	3.57
PLT (K/uL)	719	444	968	862
PDW (fL)	6.2	11.3	6.2	6.1
MPV (fL)	5.8	7.9	5.8	5.6
Morphology	2+ Polychromasia. RBC morphology within normal limits	2+ Polychromasia. RBC morphology within normal limits	3+ Polychromasia. RBC morphology within normal limits	3+ Polychromasia. RBC morphology within normal limits

RBC: Red blood cell count; HGB: hemoglobin; HCT: Hematocrit; MCV: Mean corpuscular volume; MCH: Mean Corpuscular Hemoglobin; MCHC: Mean corpuscular hemoglobin concentration; RDW: Red blood cell distribution width; RET: Reticulocyte; PLT: Platelets; PDW: Platelet Distribution Width; MPV: Mean platelet volume

Table S10. Complete metabolic profiles of tumor-free female athymic nude mice 145 d after systemic administration of saline vehicle versus varying dose levels of [^{225}Ac]Pr.

	Sample ID	#1 Saline	#2 Saline	#3 Saline	#4 Saline	#5 Saline	#1 9.25 kBq	#2 9.25 kBq	#3 9.25 kBq	#4 9.25 kBq	#5 9.25 kBq	#1 18.5 kBq	#2 18.5 kBq	#3 18.5 kBq
Renal	Sex	F	F	F	F	F	F	F	F	F	F	F	F	F
	BUN (mg/dL)	26	27	21	28	30	32	25	35	27	28	38	37	39
	CREA (mg/dL)	0.18	0.20	0.18	0.19	0.19	0.2	0.20	0.2	0.13	0.16	0.26	0.20	0.16
	BUN/CREA ratio	144.4	135.0	116.7	147.4	157.9	160.0	125.0	175.0	207.7	175.0	146.2	185.0	243.8
Hepatic Function	ALP (U/L)	69	48	66	65	61	76	90	77	60	89	107	77	87
	ALT (U/L)	38	33	35	31	25	47	36	57	33	37	38	38	51
	AST (U/L)	87	76	65	103	58	96	82	167	76	83	95	105	130
	GGT (U/L)	0	0	0	0	0	0	0	0	0	0	0	0	0
	TBIL (mg/dL)	0.2	0.2	0.2	0.1	0.1	0.2	0.1	0.4	0.2	0.2	0.1	0.2	0.2
	DBIL (mg/dL)	0.0	0.0	0.0	0.0	0.0	0.0	0.0	0.1	0.0	0.0	0.0	0.0	0.0
	IBIL (mg/dL)	0.2	0.2	0.2	0.1	0.1	0.2	0.1	0.3	0.2	0.2	0.1	0.2	0.2
	TP (g/dL)	5.2	5.4	5.5	5.8	5.1	5.2	5.4	5.7	5.3	5.3	5.6	5.6	5.3
	ALB (g/dL)	2.9	2.8	3.0	2.8	2.9	3.0	3.0	3.2	2.9	2.9	3.1	3.2	3.1
	GLOB (g/dL)	2.3	2.6	2.5	3.0	2.2	2.2	2.4	2.5	2.4	2.4	2.5	2.4	2.2
	A/G ratio	1.3	1.1	1.2	0.9	1.3	1.4	1.3	1.3	1.2	1.2	1.2	1.3	1.4
Metabolic profile	P (mg/dL)	7.5	10.2	8.2	8.2	8.1	8.4	8.3	8.2	8.1	6.9	8.4	7.6	8.4
	Ca (mg/dL)	9.0	9.3	9.0	9.4	8.9	8.8	9.1	9.0	9.0	8.5	9.0	9.1	8.9
	GLU (mg/dL)	143	158	182	157	148	154	187	156	154	151	144	156	167
	CHOL (mg/dL)	75	71	65	83	73	78	76	84	65	64	77	87	73
	TRIG (mg/dL)	91	131	80	103	69	135	81	139	200	147	170	85	95
	CK (U/L)	74	64	34	132	27	69	102	465	85	51	45	65	66
	TCO2 (mEq/L)	23	21	20	19	12	20	26	22	24	18	22	20	20
	Na (mEq/L)	156	157	152	155	156	157	155	154	153	153	154	152	151
	K (mEq/L)	7.9	8.6	7.8	8.8	8.7	7.7	8.5	7.5	7.8	7.6	8.6	7.3	8.0
	Cl (mEq/L)	113	112	109	112	112	114	111	112	110	112	112	111	111
	Na/K ratio	20	18	19	18	18	20	18	21	20	20	18	21	19
	Anion Gap	28	33	31	33	41	31	27	28	27	31	29	28	28

BUN: Blood urea nitrogen; CREA: Creatinine; ALP: Alkaline phosphatase; ALT: Alanine transaminase; AST: Aspartate transaminase; GGT: Gamma-Glutamyl Transferase; TBIL: Total bilirubin; DBIL: Direct bilirubin; IBIL: Indirect bilirubin; TP: Total protein; ALB: Albumin; GLOB: Globulin; A/G: Albumin/Globulin; P: Phosphate; Ca: Calcium; GLU: Glucose; CHOL: Cholesterol; TRIG: Triglycerides; CK: Creatine Kinase; TCO2: Total amount of carbon dioxide; Na: Sodium; K: Potassium: Cl: Chloride

Table S10, cont'd. Complete metabolic profiles of tumor-free female athymic nude mice 145 d after systemic administration of saline vehicle versus varying dose levels of [^{225}Ac]Pr.

	Sample ID	#1 37 kBq	#2 37 kBq	#3 37 kBq	#4 37 kBq	#5 37 kBq	#1 74 kBq	#2 74 kBq	#3 74 kBq	#4 74 kBq	#5 74 kBq	#1 148 kBq	#2 148 kBq	#3 148 kBq	#4 148 kBq	#5 148 kBq
Renal	Sex	F	F	F	F	F	F	F	F	F	F	F	F	F	F	F
	BUN (mg/dL)	27	29	26	30	30	31	28	27	27	26	25	27	27	28	24
	CREA (mg/dL)	0.19	0.18	0.21	0.20	0.20	0.21	0.23	0.21	0.24	0.17	0.23	0.27	0.23	0.27	0.25
	BUN/CREA ratio	142.1	161.1	123.8	150.0	150.0	147.6	121.7	128.6	112.5	152.9	108.7	100.0	117.4	103.7	96.0
Hepatic Function	ALP (U/L)	76	66	41	70	71	57	54	103	71	56	70	101	69	75	116
	ALT (U/L)	40	43	33	45	41	39	49	40	36	46	36	43	52	44	75
	AST (U/L)	162	87	107	135	108	139	81	87	84	86	84	93	150	147	165
	GGT (U/L)	0	0	0	0	0	0	0	0	0	0	0	0	0	0	0
	TBIL (mg/dL)	0.1	0.2	0.2	0.1	0.1	0.1	0.1	0.2	0.1	0.1	0.1	0.1	0.1	0.1	0.1
	DBIL (mg/dL)	0.0	0.0	0.0	0.0	0.0	0.0	0.0	0.0	0.0	0.0	0.0	0.0	0.0	0.0	0.0
	IBIL (mg/dL)	0.1	0.2	0.2	0.1	0.1	0.1	0.1	0.2	0.1	0.1	0.1	0.1	0.1	0.1	0.1
	TP (g/dL)	5.0	5.4	5.2	5.4	5.7	5.2	5.2	5.2	5.2	4.9	5.2	5.0	5.0	5.1	5.1
	ALB (g/dL)	3.0	2.9	2.7	2.9	2.7	3.1	2.9	3.0	3.2	2.9	3.1	2.9	3.1	3.1	3.0
	GLOB (g/dL)	2.0	2.5	2.5	2.5	3.0	2.1	2.3	2.2	2.0	2.0	2.1	2.1	1.9	2.0	2.1
	A/G ratio	1.5	1.2	1.1	1.2	0.9	1.5	1.3	1.4	1.6	1.5	1.5	1.4	1.6	1.6	1.4
Metabolic profile	P (mg/dL)	9.0	7.8	7.0	7.4	10.6	8.9	11.8	9.2	9.7	9.5	11.1	10.7	10.1	9.0	9.8
	Ca (mg/dL)	8.8	8.9	9.2	9.2	9.1	9.3	9.2	8.6	9.4	9.0	9.1	9.2	9.2	8.6	9.1
	GLU (mg/dL)	141	184	123	115	151	119	160	163	168	124	150	167	164	147	146
	CHOL (mg/dL)	75	54	78	69	83	82	114	87	78	91	85	112	79	94	64
	TRIG (mg/dL)	72	151	96	85	50	100	122	138	101	99	126	88	115	112	92
	CK (U/L)	416	22	166	262	184	401	57	120	13	112	97	128	330	346	215
	TCO2 (mEq/L)	26	21	22	28	20	19	23	24	12	26	26	20	23	17	21
	Na (mEq/L)	155	152	159	156	153	157	152	155	158	155	153	152	154	154	159
	K (mEq/L)	8.2	8.0	7.8	7.1	8.1	7.1	9.3	7.2	7.8	7.8	7.5	8.2	8.3	7.7	7.1
	Cl (mEq/L)	112	112	113	111	113	113	108	110	111	110	106	107	108	110	113
	Na/K ratio	19	19	20	22	19	22	16	22	20	20	20	19	19	20	22
	Anion Gap	25	27	32	24	28	32	30	28	43	27	29	33	31	35	32

BUN: Blood urea nitrogen; CREA: Creatinine; ALP: Alkaline phosphatase; ALT: Alanine transaminase; AST: Aspartate transaminase; GGT: Gamma-Glutamyl Transferase; TBIL: Total bilirubin; DBIL: Direct bilirubin; IBIL: Indirect bilirubin; TP: Total protein; ALB: Albumin; GLOB: Globulin; A/G: Albumin/Globulin; P: Phosphate; Ca: Calcium; GLU: Glucose; CHOL: Cholesterol; TRIG: Triglycerides; CK: Creatine Kinase; TCO2: Total amount of carbon dioxide; Na: Sodium; K: Potassium: Cl: Chloride

Table S10, cont'd. Complete metabolic profiles of tumor-free female athymic nude mice 145 d after systemic administration of saline vehicle versus varying dose levels of [^{225}Ac]Pr.

	Sample ID	#1 296 kBq	#2 296 kBq	#3 296 kBq	#4 296 kBq
Renal	Sex	F	F	F	F
	BUN (mg/dL)	28	36	35	34
	CREA (mg/dL)	0.24	0.28	0.22	0.19
	BUN/CREA ratio	116.7	128.6	159.1	178.9
Hepatic Function	ALP (U/L)	62	93	90	71
	ALT (U/L)	43	40	43	30
	AST (U/L)	140	90	73	100
	GGT (U/L)	0	0	0	0
	TBIL (mg/dL)	0.2	0.1	0.2	0.2
	DBIL (mg/dL)	0	0	0	0
	IBIL (mg/dL)	0.2	0.1	0.2	0.2
	TP (g/dL)	5.4	5.6	5.7	5.5
	ALB (g/dL)	3.0	3.1	2.9	3.1
	GLOB (g/dL)	2.4	2.5	2.8	2.4
	A/G ratio	1.3	1.2	1.0	1.3
Metabolic profile	P (mg/dL)	8.1	8.4	8.0	8.2
	Ca (mg/dL)	8.9	9.2	8.8	9.1
	GLU (mg/dL)	164	166	163	158
	CHOL (mg/dL)	99	82	93	98
	TRIG (mg/dL)	116	97	158	172
	CK (U/L)	192	62	35	170
	TCO2 (mEq/L)	19	21	21	22
	Na (mEq/L)	151	153	151	151
	K (mEq/L)	7.7	7.2	7.9	7.4
	Cl (mEq/L)	109	111	110	109
	Na/K ratio	20	21	19	20
	Anion Gap	31	28	28	27

BUN: Blood urea nitrogen; CREA: Creatinine; ALP: Alkaline phosphatase; ALT: Alanine transaminase; AST: Aspartate transaminase; GGT: Gamma-Glutamyl Transferase; TBIL: Total bilirubin; DBIL: Direct bilirubin; IBIL: Indirect bilirubin; TP: Total protein; ALB: Albumin; GLOB: Globulin; A/G: Albumin/Globulin; P: Phosphate; Ca: Calcium; GLU: Glucose; CHOL: Cholesterol; TRIG: Triglycerides; CK: Creatine Kinase; TCO2: Total amount of carbon dioxide; Na: Sodium; K: Potassium: Cl: Chloride

Table S11. Single-cycle HER2 α-DOTA-PRIT (296 kBq) toxicity testing in BT-474 tumor bearing female athymic nude mice 150 d after systemic administration of treatment. A total of 10 mice were evaluated at 150 d (~21 w)) p.i. of either: HER2 α-DOTA-PRIT 296 kBq (*n* = 6) or control treatments (*n* = 4; consisting of: no treatment, injection of BsAb only, or injection of [^{225}Ac]Pr 296 kBq only). Red text: Significant lesions (probably treatment-related). The lesions observed in uterus (cystic endometrial hyperplasia), oviducts (epithelial hyperplasia), urinary bladder (cystitis), and femur and tibia (hyperostosis), are common effect of estrogen administration in mice.

Animal ID	Anatomic Pathology	
	Gross Finding(s)	Microscopic Finding(s)
#1 No treatment	• Body weight is 25.472 g.	**All tissues are normal unless otherwise described.**<br>• Thymus: Thymic cysts.<br>• Kidneys: Metastatic anaplastic carcinoma, unilateral.<br>• Liver: Portal lymphocytic infiltrate, multifocal, minimal.<br>• Mesenteric lymph node: Follicular lymphoid hyperplasia.<br>• Salivary glands: Lymphocytic infiltrate, multifocal, mild.<br>• Submandibular lymph node: Follicular lymphoid hyperplasia.<br>• Uterus: Cystic endometrial hyperplasia, moderate, multifocal.<br>• Urinary bladder: Mucosal lymphocytic infiltrate, focal, mild.<br>• Ovaries: Metastatic anaplastic carcinoma, unilateral.<br>• Oviducts: Epithelial hyperplasia and hyalinosis, multifocal, unilateral, moderate.<br>• Skin (trunk): Acanthosis and hyperkeratosis, diffuse, moderate.<br>• Other: Sublumbar mass: Lymph node (presumptive site): Metastatic anaplastic carcinoma.
#2 No treatment	• Body weight is 27.156 g.	**All tissues are normal unless otherwise described.**<br>• Thymus: Thymic cysts.<br>• Kidneys: Glomerulonephritis, membranous, segmental, multifocal, bilateral, moderate.<br>• Liver: Portal lymphocytic infiltrate, multifocal, mild.<br>• Uterus: Cystic endometrial hyperplasia, marked, diffuse.<br>• Oviducts: Epithelial hyperplasia and hyalinosis, multifocal, unilateral, moderate.<br>• Skin (trunk): Acanthosis and hyperkeratosis, diffuse, moderate.<br>• Bones (femur, tibia, sternum, vertebrae): Femur and tibia, hyperstosis, mild.<br>• Harderian gland: Atrophy and fibrosis, multifocal, bilateral, moderate.<br>• Pituitary: not present on slide.<br>• Skin (trunk): Acanthosis and hyperkeratosis, diffuse, moderate.

Table S11, cont'd. Single-cycle HER2 α-DOTA-PRIT (296 kBq) toxicity testing in BT-474 tumor bearing female athymic nude mice 150 d after systemic administration of treatment. A total of 10 mice were evaluated at 150 d (~21 w)) p.i. of either: HER2 α-DOTA-PRIT 296 kBq (*n* = 6) or control treatments (*n* = 4; consisting of: no treatment, injection of BsAb only, or injection of [^{225}Ac]Pr 296 kBq only).

#1 BsAb only	• Body weight is 25.636 g.	**All tissues are normal unless otherwise described.**<br>• Thymus: Thymic cysts.<br>• Kidneys: Tubular basophilia, focal, unilateral, minimal.<br>• Liver: Portal lymphocytic infiltrate, multifocal, minimal.<br>• Mesenteric lymph node: Follicular lymphoid hyperplasia.<br>• Uterus: Cystic endometrial hyperplasia, marked, diffuse.<br>• Oviducts: Epithelial hyperplasia and hyalinosis, multifocal, bilateral, moderate.<br>• Skin (trunk): Acanthosis and hyperkeratosis, diffuse, moderate.<br>• Bones (femur, tibia, sternum, vertebrae): Femur and tibia, hyperstosis, moderate.<br>• Parathyroid: not present on slide.<br>• Other: not applicable
#1 [^{225}Ac]Pr 296 kBq only	• Body weight is 23.302 g.	**All tissues are normal unless otherwise described.**<br>• Thymus: Thymic cysts.<br>• Liver: Portal lymphocytic infiltrate, multifocal, mild.<br>• Uterus: Cystic endometrial hyperplasia, moderate, multifocal.<br>• Urinary bladder: Cystitis, neutrophilic, with urothelial hyperplasia and hemorrhage, diffuse, moderate.<br>• Oviducts: Epithelial hyperplasia and hyalinosis, multifocal, unilateral, moderate.<br>• Skin (trunk): Acanthosis and hyperkeratosis, diffuse, moderate.<br>• Bones (femur, tibia, sternum, vertebrae): Femur and tibia, hyperstosis, moderate.<br>• Mesenteric lymph node, parathyroid: not present on slide.<br>• Other: not applicable
#1 HER2 α-DOTA-PRIT (296 kBq)	• Body weight is 24.226 g.	**All tissues are normal unless otherwise described.**<br>• Lungs: Main bronchus, BALT hyperplasia, diffuse, moderate.<br>• Thymus: Thymic cysts.<br>• Kidneys: Cortical tubular atrophy and loss, multifocal, bilateral, minimal; Pelvic lymphoplasmacytic infiltrate, multifocal, bilateral, mild.<br>• Liver: Portal lymphocytic infiltrate, multifocal, minimal.<br>• Uterus: Cystic endometrial hyperplasia, mild, multifocal.<br>• Urinary bladder: Mucosal lymphocytic infiltrate, multifocal, mild.<br>• Spleen: Follicular lymphoid hyperplasia.<br>• Skin (trunk): Acanthosis and hyperkeratosis, diffuse, moderate.<br>• Bones (femur, tibia, sternum, vertebrae): Femur and tibia, hyperstosis, moderate.<br>• Harderian gland: Harderian adenitis, neutrophilic and lymphoplasmacytic, multifocal, unilateral, mild.<br>• Ears: Otitis externa, neutrophilic, unilateral, moderate.<br>• Mammary glands, pituitary: not present on slide.

Table S11, cont'd. Single-cycle HER2 α-DOTA-PRIT (296 kBq) toxicity testing in BT-474 tumor bearing female athymic nude mice 150 d after systemic administration of treatment. A total of 10 mice were evaluated at 150 d (~21 w)) p.i. of either: HER2 α-DOTA-PRIT 296 kBq (*n* = 6) or control treatments (*n* = 4; consisting of: no treatment, injection of BsAb only, or injection of [^{225}Ac]Pr 296 kBq only).

#2 HER2 α-DOTA-PRIT (296 kBq)	• Body weight is 24.888 g.	**All tissues are normal unless otherwise described.**<br>• Kidneys: Cortical tubular atrophy and loss, multifocal, bilateral, minimal.<br>• Liver: Portal lymphocytic infiltrate, multifocal, minimal; Extramedullary hematopoiesis, minimal.<br>• Uterus: Cystic endometrial hyperplasia, moderate, multifocal; Luminal dilation.<br>• Oviducts: Epithelial hyperplasia and hyalinosis, multifocal, unilateral, mild.<br>• Skin (trunk): Acanthosis and hyperkeratosis, diffuse, moderate.<br>• Thymus, parathyroid: not present on slide.
#3 HER2 α-DOTA-PRIT (296 kBq)	• Body weight is 25.863 g.	**All tissues are normal unless otherwise described.**<br>• Thymus: Thymic cysts.<br>• Kidneys: Cortical tubular atrophy and loss, multifocal, bilateral, minimal.<br>• Liver: Portal lymphocytic infiltrate, multifocal, minimal; Extramedullary hematopoiesis, minimal.<br>• Uterus: Cystic endometrial hyperplasia, moderate, multifocal; Luminal dilation.<br>• Oviducts: Epithelial hyperplasia and hyalinosis, multifocal, unilateral, mild.<br>• Skin (trunk): Acanthosis and hyperkeratosis, diffuse, moderate.<br>• Thymus, parathyroid: not present on slide.
#4 HER2 α-DOTA-PRIT (296 kBq)	• Body weight is 24.606 g.	**All tissues are normal unless otherwise described.**<br>• Thymus: Thymic cysts.<br>• Kidneys: Cortical tubular degeneration, atrophy, necrosis, and regeneration, multifocal, bilateral, mild; Glomerulonephritis, membranous, segmental, multifocal, bilateral, mild.<br>• Liver: Portal lymphocytic infiltrate, multifocal, minimal.<br>• Uterus: Cystic endometrial hyperplasia, moderate, multifocal.<br>• Spleen: White pulp, plasmacytosis.<br>• Oviducts: Epithelial hyperplasia and hyalinosis, multifocal, bilateral, moderate.<br>• Skin (trunk): Acanthosis and hyperkeratosis, diffuse, moderate.<br>• Eyes: Endophthalmitis and keratitis, neutrophilic, with fibrosis and cataract, unilateral, marked.<br>• Parathyroid, harderian gland: not present on slide.

Table S11, cont'd. Single-cycle HER2 α-DOTA-PRIT (296 kBq) toxicity testing in BT-474 tumor bearing female athymic nude mice 150 d after systemic administration of treatment. A total of 10 mice were evaluated at 150 d (~21 w)) p.i. of either: HER2 α-DOTA-PRIT 296 kBq (*n* = 6) or control treatments (*n* = 4; consisting of: no treatment, injection of BsAb only, or injection of [^{225}Ac]Pr 296 kBq only).

#5 HER2 α-DOTA-PRIT (296 kBq)	• Body weight is 25.045 g.	**All tissues are normal unless otherwise described.**<br>• Thymus: Thymic cysts.<br>• Kidneys: Cortical tubular atrophy and loss, multifocal, bilateral, minimal.<br>• Liver: Portal lymphocytic infiltrate, multifocal, mild.<br>• Uterus: Cystic endometrial hyperplasia, marked, diffuse.<br>• Spleen: White pulp, plasmacytosis.<br>• Oviducts: Epithelial hyperplasia and hyalinosis, multifocal, bilateral, moderate.<br>• Skin (trunk): Acanthosis and hyperkeratosis, diffuse, moderate.<br>• Bones (femur, tibia, sternum, vertebrae): Femur and tibia, hyperstosis, mild.<br>• Harderian gland: Atrophy and fibrosis, multifocal, unilateral, moderate.<br>• Ears: Otitis externa, neutrophilic, unilateral, marked.<br>• Submandibular lymph node, parathyroid: not present on slide.
#6 HER2 α-DOTA-PRIT (296 kBq)	• Body weight is 28.516 g.	**All tissues are normal unless otherwise described.**<br>• Thymus: Thymic cysts.<br>• Liver: Portal lymphocytic infiltrate, multifocal, minimal.<br>• Stomach: Glandular mucosa, gland ectasia, multifocal, mild.<br>• Uterus: Cystic endometrial hyperplasia, marked, diffuse.<br>• Oviducts: Epithelial hyperplasia and hyalinosis, multifocal, bilateral, moderate.<br>• Skin (trunk): Acanthosis and hyperkeratosis, diffuse, moderate.<br>• Bones (femur, tibia, sternum, vertebrae): Femur and tibia, hyperstosis, moderate.<br>• Ears: Otitis media, neutrophilic, unilateral, marked.<br>• Parathyroid: not present on slide.

Table S12. Complete automated differential blood counts of BT-474 tumor bearing female athymic nude mice 150 d after systemic administration of either: HER2 α-DOTA-PRIT 296 kBq (*n* = 6) or control treatments (*n* = 4; consisting of: no treatment, injection of BsAb only, or injection of [^{225}Ac]Pr 296 kBq only).

Test Name	#1 No treatment	#2 No treatment	#1 BsAb only	#1 [^{225}Ac]Pr 296 kBq only	#1 HER2 α-DOTA-PRIT (296 kBq)	#2 HER2 α-DOTA-PRIT (296 kBq)	#3 HER2 α-DOTA-PRIT (296 kBq)	#4 HER2 α-DOTA-PRIT (296 kBq)	#5 HER2 α-DOTA-PRIT (296 kBq)	#6 HER2 α-DOTA-PRIT (296 kBq)
WBC# (K/uL)	3.34	4.26	3.87	2.62	3.00	3.64	4.75	2.48	4.75	8.12
NEUT# (K/uL)	0.75	0.85	0.60	0.30	0.86	0.53	0.78	0.36	0.74	0.54
LYMPH# (K/uL)	2.32	3.02	2.99	1.97	2.02	2.55	3.61	1.96	3.50	5.36
MONO# (K/uL)	0.18	0.35	0.21	0.32	0.2	0.5	0.28	0.12	0.45	2.19
EO# (K/uL)	0.08	0.04	0.05	0.02	0.08	0.06	0.07	0.04	0.05	0.02
BASO# (K/uL)	0.01	0.00	0.02	0.01	0.01	0.00	0.01	0.00	0.01	0.01
NEUT (%)	22.4	20.0	15.5	11.4	23.0	14.6	16.4	14.6	15.5	6.7
LYMPH (%)	69.5	70.9	77.3	75.2	67.3	70.1	76.0	79.0	73.7	66.0
MONO (%)	5.4	8.2	5.4	12.2	6.7	13.7	5.9	4.8	9.5	27.0
EO (%)	2.4	0.9	1.3	0.8	2.7	1.6	1.5	1.6	1.1	0.2
BASO (%)	0.3	0.0	0.5	0.4	0.3	0.0	0.2	0.0	0.2	0.1

WBC: White blood cell; NEUT: Neutrophils; LYMPH: Lymphocytes; MONO: Monocytes; EO: Eosinophils; BASO: Basophils

Table S13. Complete manual differential blood counts of BT-474 tumor bearing female athymic nude mice 150 d after systemic administration of either: HER2 α-DOTA-PRIT 296 kBq (*n* = 6) or control treatments (*n* = 4; consisting of: no treatment, injection of BsAb only, or injection of [^{225}Ac]Pr 296 kBq only).

Test Name	#1 No treatment	#2 No treatment	#1 BsAb only	#1 [^{225}Ac]Pr 296 kBq only
NEUT# (K/uL)	0.73	1.15	0.35	0.37
Band# (K/uL)	0.00	0.00	0.00	0.00
LYMPH# (K/uL)	2.34	2.94	3.17	2.10
MONO# (K/uL)	0.07	0.09	0.23	0.16
EO# (K/uL)	0.02	0.04	0.18	0.00
NEUT (%)	22	27	9	14
Band (%)	---	---	---	---
LYMPH (%)	70	69	82	80
MONO (%)	2	2	6	6
EO (%)	1	2	3	---
Morphology	3+ Fragile/smudged WBCs. Due to markedly decreased WBCs on the smear the manual differential was performed with 20 cells.	3+ Fragile/smudged WBCs.	3+ Fragile/smudged WBCs.	3+ Fragile/smudged WBCs.

NEUT: Neutrophils; LYMPH: Lymphocytes; MONO: Monocytes; EO: Eosinophils.

Table S13, cont'd. Complete manual differential blood counts of BT-474 tumor bearing female athymic nude mice 150 d after systemic administration of either: HER2 α-DOTA-PRIT 296 kBq (*n* = 6) or control treatments (*n* = 4; consisting of: no treatment, injection of BsAb only, or injection of [^{225}Ac]Pr 296 kBq only).

Test Name	#1 HER2 α-DOTA-PRIT (296 kBq)	#2 HER2 α-DOTA-PRIT (296 kBq)	#3 HER2 α-DOTA-PRIT (296 kBq)	#4 HER2 α-DOTA-PRIT (296 kBq)	#5 HER2 α-DOTA-PRIT (296 kBq)	#6 HER2 α-DOTA-PRIT (296 kBq)
NEUT# (K/uL)	0.87	0.73	1.00	0.45	0.95	0.73
Band# (K/uL)	0.00	0.00	0.00	0.00	0.00	0.00
LYMPH# (K/uL)	1.86	2.88	3.37	2.01	3.61	7.39
MONO# (K/uL)	0.18	0.04	0.19	0.02	0.19	0.00
EO# (K/uL)	0.18	0.00	0.16	0.00	0.00	0.00
NEUT (%)	29	20	21	18	20	9
Band (%)	---	---	---	---	---	---
LYMPH (%)	62	79	71	81	76	91
MONO (%)	6	1	4	1	4	---
EO (%)	3	---	4	---	---	---
Morphology	3+ Fragile/smudged WBCs.	3+ Fragile/smudged WBCs.	3+ Fragile/smudged WBCs.	3+ Fragile/smudged WBCs.	3+ Fragile/smudged WBCs.	3+ Fragile/smudged WBCs. In the automated differential there is an incomplete separation of neutrophils from lymphocytes, resulting in an artifactually decreased lymphocyte count. It is recommended that the manual differential be referenced.

NEUT: Neutrophils; LYMPH: Lymphocytes; MONO: Monocytes; EO: Eosinophils.

Table S14. Complete blood counts of BT-474 tumor bearing female athymic nude mice 150 d after systemic administration of either: HER2 α-DOTA-PRIT 296 kBq (*n* = 6) or control treatments (*n* = 4; consisting of: no treatment, injection of BsAb only, or injection of [^{225}Ac]Pr 296 kBq only).

Test Name	#1 No treatment	#2 No treatment	#1 BsAb only	#1 [^{225}Ac]Pr 296 kBq only
RBC (M/uL)	8.13	8.30	9.53	8.15
HGB (g/dL)	14.0	14.8	16.5	14.3
HCT (%)	45.2	47.3	53.0	44.7
MCV (fL)	55.6	57.0	55.6	54.8
MCH (pg)	17.2	17.8	17.3	17.5
MCHC (g/dL)	31.0	31.3	31.1	32.0
RDW-SD (fL)	34.9	31.8	31.8	31.0
RDW-CV (%)	22.1	20.2	21.6	20.4
RET# (K/uL)	317.1	253.2	412.6	319.5
RET (%)	3.90	3.05	4.33	3.92
PLT (K/uL)	775.0	471.0	672.0	599.0
PDW (fL)	6.5	6.4	7.1	6.5
MPV (fL)	6.0	6.1	6.3	6.1
Morphology	3+ Polychromasia.	RBC morphology is within normal limits.	3+ Polychromasia.	3+ Polychromasia.

RBC: Red blood cell count; HGB: hemoglobin; HCT: Hematocrit; MCV: Mean corpuscular volume; MCH: Mean Corpuscular Hemoglobin; MCHC: Mean corpuscular hemoglobin concentration; RDW: Red blood cell distribution width; RET: Reticulocyte; PLT: Platelets; PDW: Platelet Distribution Width; MPV: Mean platelet volume

Table S14, cont'd. Complete blood counts of BT-474 tumor bearing female athymic nude mice 150 d after systemic administration of either: HER2 α-DOTA-PRIT 296 kBq (n = 6) or control treatments (n = 4; consisting of: no treatment, injection of BsAb only, or injection of [^{225}Ac]Pr 296 kBq only).

Test Name	#1 HER2 α-DOTA-PRIT (296 kBq)	#2 HER2 α-DOTA-PRIT (296 kBq)	#3 HER2 α-DOTA-PRIT (296 kBq)	#4 HER2 α-DOTA-PRIT (296 kBq)	#5 HER2 α-DOTA-PRIT (296 kBq)	#6 HER2 α-DOTA-PRIT (296 kBq)
RBC (M/uL)	7.77	7.74	8.07	7.96	8.61	8.31
HGB (g/dL)	12.7	12.9	14.4	13.3	14.6	14.9
HCT (%)	41.1	40.5	46.5	42.3	47.0	46.3
MCV (fL)	52.9	52.3	57.6	53.1	54.6	55.7
MCH (pg)	16.3	16.7	17.8	16.7	17.0	17.9
MCHC (g/dL)	30.9	31.9	31.0	31.4	31.1	32.2
RDW-SD (fL)	30.4	28.2	32.1	31.6	29.1	30.0
RDW-CV (%)	20.7	19.3	20.0	21.3	20.1	19.9
RET# (K/uL)	265.0	263.2	396.2	245.2	258.3	329.9
RET (%)	3.41	3.40	4.91	3.08	3.00	3.97
PLT (K/uL)	597.0	905.0	682.0	508.0	677.0	460.0
PDW (fL)	6.4	6.3	6.4	6.4	6.7	7.3
MPV (fL)	6.0	5.9	6.0	6.0	6.2	7.0
Morphology	3+ Polychromasia.	3+ Polychromasia.	RBC morphology is within normal limits.	3+ Polychromasia.	3+ Polychromasia.	3+ Fragile/smudged WBCs.

RBC: Red blood cell count; HGB: hemoglobin; HCT: Hematocrit; MCV: Mean corpuscular volume; MCH: Mean Corpuscular Hemoglobin; MCHC: Mean corpuscular hemoglobin concentration; RDW: Red blood cell distribution width; RET: Reticulocyte; PLT: Platelets; PDW: Platelet Distribution Width; MPV: Mean platelet volume

Table S15. Complete metabolic profiles of BT-474 tumor bearing female athymic nude mice 150 d after systemic administration of either: HER2 α-DOTA-PRIT 296 kBq (*n* = 6) or control treatments (*n* = 4; consisting of: no treatment, injection of BsAb only, or injection of [^{225}Ac]Pr 296 kBq only).

	Sample ID	#1 No treatment	#2 No treatment	#1 BsAb only	#1 [^{225}Ac]Pr 296 kBq only	#1 HER2 α-DOTA-PRIT (296 kBq)	#2 HER2 α-DOTA-PRIT (296 kBq)	#3 HER2 α-DOTA-PRIT (296 kBq)	#4 HER2 α-DOTA-PRIT (296 kBq)	#5 HER2 α-DOTA-PRIT (296 kBq)	#6 HER2 α-DOTA-PRIT (296 kBq)
Renal	Sex	F	F	F	F	F	F	F	F	F	F
	BUN (mg/dL)	27	26	23	31	26	30	31	32	30	31
	CREA (mg/dL)	0.18	0.19	0.22	0.12	0.26	0.25	0.19	0.25	0.20	0.17
	BUN/CREA ratio	150.0	136.8	104.5	258.3	100.0	120.0	163.2	128.0	150.0	182.4
Hepatic Function	ALP (U/L)	49	50	57	167	94	83	48	53	133	108
	ALT (U/L)	16	31	18	30	28	35	19	25	37	59
	AST (U/L)	53	87	58	85	84	89	52	83	77	224
	GGT (U/L)	0	0	0	0	0	0	0	0	0	0
	TBIL (mg/dL)	0.2	0.2	0.2	0.2	0.1	0.1	0.2	0.1	0.2	0.2
	DBIL (mg/dL)	0.0	0.0	0.0	0.0	0.0	0.0	0.0	0	0.0	0.0
	IBIL (mg/dL)	0.2	0.2	0.2	0.2	0.1	0.1	0.2	0.1	0.2	0.2
	TP (g/dL)	5.0	4.7	5.3	4.8	5.7	5.2	5.0	5.5	5.2	5.1
	ALB (g/dL)	2.9	2.7	3.0	2.9	3.1	3.1	2.9	2.8	2.9	2.9
	GLOB (g/dL)	2.1	2	2.3	1.9	2.6	2.1	2.1	2.7	2.3	2.2
	A/G ratio	0.7	0.7	0.8	0.7	0.8	0.7	0.7	1.0	0.8	0.8
Metabolic profile	P (mg/dL)	8.0	7.4	8.8	9.2	8.3	7.6	8.2	7.3	7.1	8.3
	Ca (mg/dL)	8.9	9.0	9.3	8.9	9.3	9.2	8.7	8.6	9.2	9.4
	GLU (mg/dL)	151	150	157	148	147	164	183	156	162	221
	CHOL (mg/dL)	124	101	132	123	116	108	121	134	111	142
	TRIG (mg/dL)	79	89	83	80	73	77	129	96	113	137
	CK (U/L)	70	91	76	84	100	94	28	123	88	701
	TCO2 (mEq/L)	25	27	26	24	23	25	23	27	25	25
	Na (mEq/L)	152	153	156	154	156	154	152	156	155	153
	K (mEq/L)	7.3	7.2	8.8	8.9	7.1	7.5	7.5	6.5	7.5	8.6
	Cl (mEq/L)	110	107	110	112	113	113	111	113	112	110
	Na/K ratio	21	21	18	17	22	21	20	24	21	18
	Anion Gap	24	26	29	27	27	24	26	23	26	27

BUN: Blood urea nitrogen; CREA: Creatinine; ALP: Alkaline phosphatase; ALT: Alanine transaminase; AST: Aspartate transaminase; GGT: Gamma-Glutamyl Transferase; TBIL: Total bilirubin; DBIL: Direct bilirubin; IBIL: Indirect bilirubin; TP: Total protein; ALB: Albumin; GLOB: Globulin; A/G: Albumin/Globulin; P: Phosphate; Ca: Calcium; GLU: Glucose; CHOL: Cholesterol; TRIG: Triglycerides; CK: Creatine Kinase; TCO2: Total amount of carbon dioxide; Na: Sodium; K: Potassium: Cl: Chloride

Table S16. Single-cycle GD2 α-DOTA-PRIT (37 kBq) toxicity testing in IMR-32/luc tumor bearing female athymic nude mice 141-241 d after systemic administration of treatment. A total of 9 mice were evaluated at 141-241 d p.i. of either: GD2 α-DOTA-PRIT 37 kBq (*n* = 4) or tumor-free age-matched littermate controls (*n* = 5). Red text: Significant lesions (probably treatment-related).

Animal ID	Anatomic Pathology	
	Gross Finding(s)	Microscopic Finding(s)
#1 Age-match littermate	• Age is 11.1 Months<br>• Body weight is 25.198 g<br>• Right kidney is spherical (0.7 x 0.6 x 0.4)	**All tissues are normal unless otherwise described.**<br>• Thymus: not present on slide.<br>• Kidneys: tubular dilation (medulla), multifocal, mild, unilateral.<br>• Spleen: lymphoid hyperplasia, diffuse, minimal.<br>• Adrenals: subcapsular cell hyperplasia, multifocal, mild, bilateral.<br>• Parathyroid: not present on slide.<br>• Skin (trunk): Epidermal hyperplasia, multifocal, moderate; Dermal fibrosis, multifocal, minimal; Orthokeratotic hyperkeratosis, multifocal, mild.<br>• Bones (femur, tibia, sternum, vertebrae): degeneration intervertebral discs, multifocal, mild.
#2 Age-match littermate	• Age is 11.1 Months<br>• Body weight is 32.16 g.	**All tissues are normal unless otherwise described.**<br>• Thymus: not present on slide.<br>• Spleen: lymphoid hyperplasia, diffuse, mild<br>• Adrenals: subcapsular cell hyperplasia, multifocal, mild, bilateral.<br>• Skin (trunk): Epidermal hyperplasia, multifocal, mild; Orthokeratotic hyperkeratosis, multifocal, minimal.<br>• Harderian gland: fibrosis, focal, minimal, unilateral.
#3 Age-match littermate	• Age is 7.2 months<br>• Body weight is 27.591 g.<br>• Mild splenomegaly	**All tissues are normal unless otherwise described.**<br>• Thymus: not present on slide.<br>• Spleen: lymphoid hyperplasia, diffuse, mild.<br>• Adrenals: subcapsular cell hyperplasia, diffuse, mild, bilateral.<br>• Parathyroid: not present on slide<br>• Skin: Epidermal hyperplasia, multifocal, moderate; Dermal fibrosis, multifocal, mild; Orthokeratotic hyperkeratosis, multifocal, mild.

Table S16, con. Single-cycle GD2 α-DOTA-PRIT (37 kBq) toxicity testing in IMR-32/luc tumor bearing female athymic nude mice 141-241 d after systemic administration of treatment. A total of 9 mice were evaluated at 141-241 d p.i. of either: GD2 α-DOTA-PRIT 37 kBq (*n* = 4) or tumor-free age-matched littermate controls (*n* = 5).

#4 Age-match littermate	• Age is 7.2 months<br>• Body weight is 27.005 g.	**All tissues are normal unless otherwise described.**<br>• Lungs: peribronchial lymphocytic infiltrate, multifocal, moderate.<br>• Thymus: not present on slide.<br>• Liver: peribiliary lymphocytic infiltrate, multifocal, mild.<br>• Gall Bladder: lymphocytic infiltrate, focal, mild<br>• Mesenteric lymph node: lymphoid hyperplasia and plasmacytosis, diffuse, moderate.<br>• Salivary Glands: lymphoplasmacytic infiltrates, multifocal, moderate.<br>• Submandibular lymph node: lymphoid hyperplasia and plasmacytosis, diffuse, mild.<br>• Adrenals: subcapsular cell hyperplasia, diffuse, mild, bilateral.<br>• Parathyroid: not present on slide.<br>• Skin (trunk): Epidermal hyperplasia, multifocal, moderate; Dermal fibrosis, multifocal, mild; Orthokeratotic hyperkeratosis, multifocal, mild.<br>• Harderian Gland: lymphoplasmacytic infiltrates, multifocal, mild.<br>• Ears: lymphoplamacytic infiltrates middle ear, multifocal, minimal, bilateral.
#5 Age-match littermate	• Age is 7.2 months<br>• Body weight is 27.591 g.	**All tissues are normal unless otherwise described.**<br>• Lungs: peribronchial lymphocytic infiltrate, focal, minimal.<br>• Thymus: not present on slide.<br>• Uterus: Cystic endometrial hyperplasia, diffuse, minimal.<br>• Urinary Bladder: lymphocytic infiltrates multifocal, mild.<br>• Adrenals: subcapsular cell hyperplasia, multifocal, mild, bilateral.<br>• Parathyroid: not present on slide.<br>• Skin (trunk): Epidermal hyperplasia, multifocal, moderate; Dermal fibrosis, multifocal, minimal; Orthokeratotic hyperkeratosis, multifocal, minimal.<br>• Bones (femur, tibia, sternum, vertebrae): Femur and tibia, hyperostosis, mild.<br>• Harderian gland: Atrophy and fibrosis, multifocal, moderate, unilateral.<br>• Ears: Otitis externa, neutrophilic, marked, unilateral.<br>• Submandibular lymph node, parathyroid: not present on slide.

Table S16, con. Single-cycle GD2 α-DOTA-PRIT (37 kBq) toxicity testing in IMR-32/luc tumor bearing female athymic nude mice 141-241 d after systemic administration of treatment. A total of 9 mice were evaluated at 141-241 d p.i. of either: GD2 α-DOTA-PRIT 37 kBq (*n* = 4) or tumor-free age-matched littermate controls (*n* = 5).

#1 GD2 α-DOTA-PRIT 37 kBq	• Age is 11.1 Months<br>• Body weight is 29.058 g.	**All tissues are normal unless otherwise described.**<br>• Lungs: peribronchial lymphocytic infiltrate, focal, mild.<br>• Thymus: not present on slide.<br>• Kidneys: hyaline cast medullary tubules, focal, minima, unilateral.<br>• Uterus: stromal polyp, focal, mild. Cystic endometrial hyperplasia, focal, mild.<br>• Spleen: lymphoid hyperplasia, diffuse, mild.<br>• Skin (trunk): Epidermal hyperplasia, multifocal, moderate; Dermal fibrosis, multifocal, minimal; Orthokeratotic hyperkeratosis, multifocal, minimal.<br>• Bones (femur, tibia, sternum, vertebrae): degeneration intervertebral discs, multifocal, mild.
#2 GD2 α-DOTA-PRIT 37 kBq	• Age is 7.2 months<br>• Body weight is 26.681 g<br>• Mild splenomegaly	**All tissues are normal unless otherwise described.**<br>• Thymus: not present on slide.<br>• Kidneys: only 1 kidney present.<br>• Uterus: Cystic endometrial hyperplasia, diffuse, mild.<br>• Spleen: lymphoid hyperplasia, diffuse, minimal.<br>• Adrenals: subcapsular cell hyperplasia, MF, 2, BL.<br>• Skin (trunk): Epidermal hyperplasia, multifocal, moderate; Dermal fibrosis, multifocal, minimal; Orthokeratotic hyperkeratosis, multifocal, moderate<br>• Bones (femur, tibia, sternum, vertebrae): Femur and tibia, hyperostosis, mild.<br>• Mesenteric lymph node, parathyroid: not present on slide.
#3 GD2 α-DOTA-PRIT 37 kBq	• Age is 7.2 months<br>• Body weight is 28.081 g.	• **All tissues are normal unless otherwise described.**<br>• Thymus: not present on slide.<br>• Uterus: Cystic endometrial hyperplasia, diffuse, moderate.<br>• Adrenals: subcapsular cell hyperplasia, multifocal, mild, bilateral.<br>• Parathyroid: not present on slide.<br>• Skin (trunk): Epidermal hyperplasia, multifocal, moderate; Dermal fibrosis, multifocal, moderate; Orthokeratotic hyperkeratosis, multifocal, moderate.<br>• Bones (femur, tibia, sternum, vertebrae): Femur and tibia, hyperostosis, moderate.<br>• Pituitary: not present on slide.
#4 GD2 α-DOTA-PRIT 37 kBq	• Age is 7.2 months<br>• Body weight is 27.005 g.<br>• Mild splenomegaly and iliac lymphadenomegaly	**All tissues are normal unless otherwise described.**<br>• Lungs: peribronchial lymphocytic infiltrate, focal, minimal.<br>• Thymus: cyst, diffuse, moderate.<br>• Spleen: lymphoid hyperplasia, diffuse, mild.<br>• Adrenals: subcapsular cell hyperplasia, multifocal, mild, unilateral.<br>• Parathyroid: not present on slide.<br>• Skin (trunk): Epidermal hyperplasia, multifocal, moderate; Dermal fibrosis, multifocal, minimal; Orthokeratotic hyperkeratosis, multifocal, mild.<br>• Thymus, parathyroid: not present on slide.

Table S17. Complete automated differential blood counts of IMR-32 tumor bearing female athymic nude mice 141-241 d after systemic administration of either: GD2 α-DOTA-PRIT 37 kBq (n = 4) or age-matched littermate controls (n = 5).

Test Name	#1 Age-match littermate	#2 Age-match littermate	#3 Age-match littermate	#4 Age-match littermate	#5 Age-match littermate	#1 GD2 α-DOTA-PRIT 37 kBq	#2 GD2 α-DOTA-PRIT 37 kBq	#3 GD2 α-DOTA-PRIT 37 kBq	#4 GD2 α-DOTA-PRIT 37 kBq
WBC# (K/uL)	5.61	3.49	7.66	3.96	4.28	6.2	5.64	11.18	10.92
NEUT# (K/uL)	1.06	0.8	2.22	1.02	0.82	1.19	1.18	1.57	1.92
LYMPH# (K/uL)	4.16	2.08	4.84	2.48	3.11	4.64	3.94	9.05	7.11
MONO# (K/uL)	0.27	0.55	0.46	0.31	0.28	0.22	0.4	0.38	1.75
EO# (K/uL)	0.12	0.06	0.13	0.14	0.07	0.15	0.12	0.17	0.13
BASO# (K/uL)	0.00	0.00	0.01	0.01	0.00	0.00	0.00	0.01	0.01
NEUT (%)	18.9	22.9	29	25.8	19.2	19.3	20.9	14.1	17.6
LYMPH (%)	74.2	59.6	63.2	62.6	72.7	74.8	69.9	80.9	65.1
MONO (%)	4.8	15.8	6	7.8	6.5	3.5	7.1	3.4	16
EO (%)	2.1	1.7	1.7	3.5	1.6	2.4	2.1	1.5	1.2
BASO (%)	0.0	0.0	0.1	0.3	0.0	0.0	0.0	0.1	0.1

WBC: White blood cell; NEUT: Neutrophils; LYMPH: Lymphocytes; MONO: Monocytes; EO: Eosinophils; BASO: Basophils

Table S18. Complete manual differential blood counts of IMR-32 tumor bearing female athymic nude mice 141-241 d after systemic administration of either: GD2 α-DOTA-PRIT 37 kBq (*n* = 4) or age-matched littermate controls (*n* = 5).

Test Name	#1 Age-match littermate	#2 Age-match littermate	#3 Age-match littermate	#4 Age-match littermate	#5 Age-match littermate	#1 GD2 α-DOTA-PRIT 37 kBq	#2 GD2 α-DOTA-PRIT 37 kBq	#3 GD2 α-DOTA-PRIT 37 kBq	#4 GD2 α-DOTA-PRIT 37 kBq
NEUT# (K/uL)	2.12	1.42	2.22	1.03	0.77	1.36	1.23	0.98	1.3
Band# (K/uL)	0.00	0.00	0.00	0.00	0.00	0.00	0.00	0.00	0.00
LYMPH# (K/uL)	8.61	9.28	5.21	2.69	3.08	4.53	3.98	2.34	3.95
MONO# (K/uL)	0.45	0.22	0.00	0.24	0.30	0.25	0.34	0.1	0.11
EO# (K/uL)	0.00	0.00	0.00	0.00	0.21	0.04	0.06	0.06	0.1
NEUT (%)	19	13	29	26	18	22	22	28	23
Band (%)	---	---	---	---	---	---	---	---	---
LYMPH (%)	77	85	68	68	72	73	71	67	70
MONO (%)	4	2	---	6	7	4	6	3	2
EO (%)	---	---	3	---	3	1	1	2	5
Morphology	3+ Fragile/ smudged WBCs.	Moderate amount of large lymphocytes seen.	WBC morphology is within normal limits.	WBC morphology is within normal limits.	3+ Fragile/ smudged WBCs.	WBC morphology is within normal limits.	3+ Fragile/ smudged WBCs.	WBC morphology is within normal limits.	WBC morphology is within normal limits.

Legend: <1= rare (to be used for all RBC descriptions EXCEPT for polychromasia); 1+= minimal, 2+= mild, 3+= moderate, 4+= marked.

Table S19. Complete metabolic profiles of IMR-32 tumor bearing female athymic nude mice 141-241 d after systemic administration of either: anti-GD2 ^{225}Ac-DOTA-PRIT 37 kBq (n = 4) or age-matched littermate controls (n = 5).

	Sample ID	#1 Age-match littermate	#2 Age-match littermate	#3 Age-match littermate	#4 Age-match littermate	#5 Age-match littermate	#1 GD2 α-DOTA-PRIT 37 kBq	#2 GD2 α-DOTA-PRIT 37 kBq	#3 GD2 α-DOTA-PRIT 37 kBq	#4 GD2 α-DOTA-PRIT 37 kBq
Renal	Sex	F	F	F	F	F	F	F	F	F
	BUN (mg/dL)	21	36	27	32	37	44	23	25	33
	CREA (mg/dL)	0.21	0.19	0.20	0.12	0.22	0.20	0.18	0.21	0.25
	BUN/CREA ratio	100.0	189.5	135.0	266.7	168.2	220.0	127.8	119.0	132.0
Hepatic Function	ALP (U/L)	72	52	52	47	61	77	68	106	65
	ALT (U/L)	28	28	24	42	34	27	22	25	115
	AST (U/L)	60	70	61	107	106	87	68	64	166
	GGT (U/L)	0	0	0	0	0	0	0	0	0
	TBIL (mg/dL)	0.1	0.2	0.2	0.2	0.2	0.2	0.2	0.1	0.1
	DBIL (mg/dL)	0.0	0.0	0.0	0.0	0.0	0.0	0.0	0.0	0.0
	IBIL (mg/dL)	0.1	0.2	0.2	0.2	0.2	0.2	0.2	0.1	0.1
	TP (g/dL)	5.1	4.7	4.6	4.8	5.2	4.7	4.5	4.8	4.8
	ALB (g/dL)	3.0	3.0	2.8	2.9	3.1	3.0	2.9	3.0	2.9
	GLOB (g/dL)	2.1	1.7	1.8	1.9	2.1	1.7	1.6	1.8	1.9
	A/G ratio	1.4	1.8	1.6	1.5	1.5	1.8	1.8	1.7	1.5
Metabolic profile	P (mg/dL)	5.6	8.2	8.1	8.5	8.0	7.0	7.2	8.1	7.8
	Ca (mg/dL)	9.3	9.2	8.8	8.8	8.6	8.7	9.0	9.2	9.1
	GLU (mg/dL)	209	186	186	164	203	157	184	216	220
	CHOL (mg/dL)	121	96	97	104	108	98	102	128	134
	TRIG (mg/dL)	158	148	148	217	183	152	138	138	147
	CK (U/L)	63	51	60	50	266	90	142	41	224
	TCO2 (mEq/L)	32	27	27	28	30	28	24	29	25
	Na (mEq/L)	152	151	153	156	156	153	153	153	152
	K (mEq/L)	6.6	7.8	7.7	7.8	7.6	7.4	7.7	7.9	7.7
	Cl (mEq/L)	107	110	113	114	111	113	110	110	112
	Na/K ratio	23	19	20	20	21	21	20	19	20
	Anion Gap	20	22	21	22	23	19	27	22	23

BUN: Blood urea nitrogen; CREA: Creatinine; ALP: Alkaline phosphatase; ALT: Alanine transaminase; AST: Aspartate transaminase; GGT: Gamma-Glutamyl Transferase; TBIL: Total bilirubin; DBIL: Direct bilirubin; IBIL: Indirect bilirubin; TP: Total protein; ALB: Albumin; GLOB: Globulin; A/G: Albumin/Globulin; P: Phosphate; Ca: Calcium; GLU: Glucose; CHOL: Cholesterol; TRIG: Triglycerides; CK: Creatine Kinase; TCO2: Total amount of carbon dioxide; Na: Sodium; K: Potassium: Cl: Chloride

References

1. Aime S, Botta M, Fasano M, Marques MP, Geraldes CF, Pubanz D, et al. Conformational and Coordination Equilibria on DOTA Complexes of Lanthanide Metal Ions in Aqueous Solution Studied by (1)H-NMR Spectroscopy. Inorganic chemistry. 1997;36(10):2059-68.
2. Dogdas B, Stout D, Chatziioannou AF, Leahy RM. Digimouse: a 3D whole body mouse atlas from CT and cryosection data. Phys Med Biol. 2007;52(3):577-87.
3. Cheal SM, Fung EK, Patel M, Xu H, Guo HF, Zanzonico PB, et al. Curative Multicycle Radioimmunotherapy Monitored by Quantitative SPECT/CT-Based Theranostics, Using Bispecific Antibody Pretargeting Strategy in Colorectal Cancer. J Nucl Med. 2017;58(11):1735-42.
4. Cheal SM, Xu H, Guo HF, Lee SG, Punzalan B, Chalasani S, et al. Theranostic pretargeted radioimmunotherapy of colorectal cancer xenografts in mice using picomolar affinity (8)(6)Y- or (1)(7)(7)Lu-DOTA-Bn binding scFv C825/GPA33 IgG bispecific immunoconjugates. Eur J Nucl Med Mol Imaging. 2016;43(5):925-37.
